# CUB domain-containing protein 1 and the epidermal growth factor receptor cooperate to induce cell detachment

**DOI:** 10.1186/s13058-016-0741-1

**Published:** 2016-08-05

**Authors:** Mary E. Law, Renan B. Ferreira, Bradley J. Davis, Paul J. Higgins, Jae-Sung Kim, Ronald K. Castellano, Sixue Chen, Hendrik Luesch, Brian K. Law

**Affiliations:** 1 Department of Pharmacology and Therapeutics, University of Florida, Acad. Res. Bldg., Room R5-210, 1200 Newell Drive, P.O. Box 100267, Gainesville, FL 32610 USA; 2 UF-Health Cancer Center, University of Florida, Gainesville, FL 32610 USA; 3 Department of Chemistry, University of Florida, Gainesville, FL 32611 USA; 4 Center for Cell Biology and Cancer Research, Albany Medical College, Albany, NY 12208 USA; 5 Department of Surgery, University of Florida, Gainesville, FL 32610 USA; 6 Department of Biology, Interdisciplinary Center for Biotechnology, University of Florida, Gainesville, FL 32611 USA; 7 Department of Medicinal Chemistry, University of Florida, Gainesville, FL 32610 USA; 8 Center for Natural Products, Drug Discovery and Development (CNPD3), University of Florida, Gainesville, FL 32610 USA

**Keywords:** CDCP1, EGFR, Src, Adhesion, E-cadherin, Breast cancer

## Abstract

**Background:**

While localized malignancies often respond to available therapies, most disseminated cancers are refractory. Novel approaches, therefore, are needed for the treatment of metastatic disease. CUB domain-containing protein1 (CDCP1) plays an important role in metastasis and drug resistance; the mechanism however, is poorly understood.

**Methods:**

Breast cancer cell lines were engineered to stably express EGFR, CDCP1 or phosphorylation site mutants of CDCP1. These cell lines were used for immunoblot analysis or affinity purification followed by immunoblot analysis to assess protein phosphorylation and/or protein complex formation with CDCP1. Kinase activity was evaluated using phosphorylation site-specific antibodies and immunoblot analysis in in vitro kinase assays. Protein band excision and mass spectrometry was utilized to further identify proteins complexed with CDCP1 or ΔCDCP1, which is a mimetic of the cleaved form of CDCP1. Cell detachment was assessed using cell counting.

**Results:**

This paper reports that CDCP1 forms ternary protein complexes with Src and EGFR, facilitating Src activation and Src-dependent EGFR transactivation. Importantly, we have discovered that a class of compounds termed Disulfide bond Disrupting Agents (DDAs) blocks CDCP1/EGFR/Src ternary complex formation and downstream signaling. CDCP1 and EGFR cooperate to induce detachment of breast cancer cells from the substratum and to disrupt adherens junctions. Analysis of CDCP1-containing complexes using proteomics techniques reveals that CDCP1 associates with several proteins involved in cell adhesion, including adherens junction and desmosomal cadherins, and cytoskeletal elements.

**Conclusions:**

Together, these results suggest that CDCP1 may facilitate loss of adhesion by promoting activation of EGFR and Src at sites of cell-cell and cell-substratum contact.

**Electronic supplementary material:**

The online version of this article (doi:10.1186/s13058-016-0741-1) contains supplementary material, which is available to authorized users.

## Background

The CUB domain-containing protein 1 (CDCP1) [1–3], has been implicated in tumor resistance to cytotoxic chemotherapy agents such as gemcitabine [4], and also allows cancer cells to resist cell death induced by targeted therapeutics such as next-generation BCR-ABL inhibitors [5], and the human epidermal growth factor receptor 2 (HER2)-targeted monoclonal antibody trastuzumab (Herceptin) [6]. CDCP1 is a single-pass transmembrane protein with three extracellular CUB domains and a short intracellular tail. Tyrosine phosphorylation of the intracellular domain of CDCP1 results in downstream signaling through Src-family kinases (SFKs), Akt, and PKCδ [7–11]. The mechanisms that regulate CDCP1 tyrosine phosphorylation, however, are incompletely understood.

CDCP1 likely contributes to metastasis, in part, by allowing cancer cells to survive and metastasize in the absence of attachment. In the MDA-MB-468 breast cancer cell line, enforced CDCP1 expression induces cell detachment and growth in suspension even in the presence of a suitable adhesive substrate [12]. CDCP1-mediated cell detachment is not observed universally, and how CDCP1 causes suspension growth in specific circumstances is unknown. Clarification of specific mechanisms by which CDCP1 induces cell detachment could provide valuable insights into how CDCP1 promotes metastasis, highlighting the importance of CDCP1 as a therapeutic target.

This paper reports that CDCP1 forms a ternary complex with Src and the EGFR, and that this complex mediates Src activation and Src-dependent tyrosine phosphorylation of CDCP1 and EGFR (i.e., EGFR transactivation). Furthermore, enforced expression of CDCP1 and EGFR cooperate to induce cell detachment from the substratum, and this effect is enhanced by stimulation of the cells with EGF. Together the results suggest that a novel CDCP1/EGFR/Src ternary complex activates several signaling responses that contribute to metastasis. These mechanisms include Src activation, CDCP1 tyrosine phosphorylation, and EGFR transactivation. Importantly, studies carried out with a new class of anti-cancer agents (i.e., Disulfide bond Disrupting Agents [DDAs]), which target epidermal growth factor receptor (EGFR) and its family members HER2 and HER3 [13], show that DDAs disrupt CDCP1 ternary signaling complexes.

Analysis of CDCP1-containing complexes using proteomics techniques revealed that CDCP1 associates with proteins involved in cell-cell and cell-substratum adhesion. These studies identified Galectin-1 and matrix metalloproteinase 14 (MMP-14) among the repertoire of proteins that preferentially associate with the full length or cleaved forms of CDCP1, respectively. The results suggest that the CDCP1/Src/EGFR complex is a novel, druggable target and that DDAs may be useful in abrogating the pro-metastatic functions of this signaling platform. Results presented here, along with previously published studies [11, 14], reveal that CDCP1 functions as a protein-protein interaction hub that interfaces with the signaling proteins and structural elements that control cell-cell and cell-substratum adhesion in a manner that is regulated by CDCP1 proteolytic processing and tyrosine phosphorylation.

## Methods

### Cell culture, recombinant retroviruses, and construction of stable cell lines

Cell lines were purchased from ATCC (Manassas, VA, USA). EGF (GF001) was obtained from Chemicon International (Temecula, CA, USA). Dasatinib (S1021), lapatinib (sc-202205), and GM6001 (CC1010) were from Selleckchem (Houston, TX, USA), Santa Cruz Biotechnology (Dallas, TX, USA), and EMD Millipore (Billerica, MA, USA), respectively. NSC624192, NSC624197, NSC333839, NSC624203, and NSC624205 were gifts from the National Cancer Institute’s Developmental Therapeutics Program. RBF3 was synthesized as described previously [13].

A retroviral vector encoding EGFR (plasmid 11011, [15]) and an expression vector encoding His_6_-Myc tagged CDCP1 (plasmid 31768 [12]) were from Addgene (Cambridge, MA, USA). Retroviral vectors encoding CDCP1 were prepared using the pMXS-IRES-Blasticidin plasmid (RTV-016) (Cell Biolabs, Inc., San Diego, CA, USA). Recombinant retrovirus was prepared and used to produce stable cell lines as described previously [16]. The Y707F CDCP1 mutant was prepared using the primers:

Forward: CCGCTGTGGGTATCTTCAATGACAACATC; reverse: GATGTTGTCATTGAAGATACCCACAGCGG. The Y734F CDCP1 mutant was prepared using the primers: forward: AATGACTCCCATGTGTTTGCAGTCATCGAGGAC; reverse: GTCCTCGATGACTGCAAACACATGGGAGTCATT. The Y762F mutant of CDCP1 was produced using the primers: forward: CTGCAGCCAGAGGTGGACACCTTCCGGCCGTTCCAGGGCACCATG; reverse: CATGGTGCCCTGGAACGGCCGGAAGGTGTCCACCTCTGGCTGCAG. These primers were also used to generate the 3YF (Y707F, Y734F, and Y762F) triple mutant. The mutant CDCP1 constructs were verified by DNA sequencing.

### Construction of PKC™ adenovirus

The pLTR PKC™ plasmid encoding mouse PKC™ was obtained from Addgene (Addgene plasmid # 8419) and was used as a template for PCR to add a 5′ HindIII site and Myc tag and a 3′ EcoRV site using the following primers: forward: TTTTAAGCTTATGGAACAAAAACTTATTTCCGAAGAAGACCTTGCACCCTTCCTGCGCATCTC and reverse: TTTTGATATCTTAAATGTCCAGGAATTGC. The resultant PCR product was then cloned into the 5′ HindIII and 3′ EcoRV sites of pcDNA3. The PKC™/pcDNA3 vector was subsequently digested with HindIII and EcoRV and the PKC™ insert was cloned into the 5′ HindIII and 3′ EcoRV sites of pAdTrack-CMV. The PKC™/pAdTrack-CMV vector was recombined with pAdEasy1 in BJ5183-AD-1 (Agilent Technologies, Santa Clara, CA, USA) cells using an electroporator set at 200 Ohms, 25 mF and 2.5 kV. Recombinant PKC™/pAdEasy1 was then linearized with Pac I and transfected into HEK 293 A cells using lipofectamine (Invitrogen, Carlsbad, CA, USA), according to manufacturer’s instructions. Adenovirus expressing PKC™ was subsequently amplified in HEK 293A cells.

### Affinity purification and immunoblot

Affinity purification using anti-FLAG agarose (Millipore Sigma, St. Louis, MO, USA), glutathione-agarose (Millipore Sigma), and TALON resin (Clontech, Mountain View, CA, USA) was done as described [16–19]. Immunoblot analysis was performed using antibodies purchased from Santa Cruz Biotechnology [(Myc tag (9E10), sc-40; actin, sc-1616-R; dynein heavy chain, sc-9115; ß-catenin, sc-7199; γ-catenin, sc-7900; Src, sc-18; desmoplakin, sc-33555; c-Myc, sc-764; Enigma, sc-98370; Galectin 1, sc-28248; EGFR, sc-03; phosphotyrosine (PY99), sc-7020], Cell Signaling Technology (Beverly, MA, USA) [EGFR, #4267; c-Met, #3127; HER2, #2165; PKCδ, #2058; phospho-PKCδ[Y311], # 2055; CDCP1, #4115 and #13794; P-CDCP1[Y707], #13111; P-CDCP1[Y734], #9050; P-CDCP1[Y743], #13093; P-CDCP1[Y806], #13024; β1-integrin, #4706; MMP14, #13130; P-EGFR[Y845], #6963; P-EGFR[Y992], #2235; P-Src[Y416], #6943; p62, #5114; α-E-catenin, #3236], BD Transduction Laboratories (San Jose, CA, USA) [E-cadherin, 610182; plasminogen activator inhibtor-1 (PAI-1), 612024], BD Biosciences (San Jose, CA, USA) [p120 catenin, 610133], Neomarkers (Freemont, CA, USA) [cyclin D1, MS-210], EMD Millipore (Temecula, CA, USA) [anti-phosphotyrosine (4G10), 05-321], Invitrogen [desmoglein 2, 32-6100], Qiagen (Valencia, CA, USA) [anti-His_5_, 34660], and Millipore Sigma [anti-FLAG (M2), F3165].

### Surface biotinylation

Labeling of cell surface proteins was performed using Sulfo-NHS-SS-Biotin (Thermo Fisher Scientific, Rockford, IL, USA) essentially as described previously [20]. Briefly, cells treated as described in the figure legends were labeled for 10 minutes at 4 °C with 1.6 mM Sulfo-NHS-SS-Biotin in phosphate-buffered saline (PBS), pH 8.0. The cells were washed with PBS, and extracted in 1.0 % Triton X100 extraction buffer without reducing agents. Biotinylated proteins were isolated using streptavidin-agarose beads (Thermo Fisher Scientific) and non-biotinylated proteins (the flow-through) were retained.

### In vitro kinase assays

Kinase assays were performed as detailed [21] with the exception that γ-^32^P-ATP was omitted and phosphorylation of proteins in the assays was detected using the appropriate phosphorylation site-specific antibodies by immunoblot analysis.

### Imaging intracellular E-cadherin localization

E-cadherin intracellular localization was assessed by confocal microscopy of cells stably expressing an E-cadherin-GFP fusion protein as described previously [16].

### Identification of CDCP1-associated proteins using proteomics techniques

The protein bands were enzymatically digested with trypsin as previously described [22]. The tryptic digest was injected onto a capillary trap (LC Packings PepMap, Thermo Fisher Scientific) and washed for 5 min with a flow rate of 5 μL/min of 0.1 % v/v acetic acid. The samples were loaded onto an LC Packing® C18 Pep Map HPLC column. The elution gradient of the HPLC column started at 97 % solvent A, 3 % solvent B and finished at 40 % solvent A, 60 % solvent B for 60 min for protein identification. Solvent A consisted of 0.1 % v/v acetic acid, 3 % v/v acetonitrile (ACN), and 96.9 % v/v H_2_O. Solvent B consisted of 0.1 % v/v acetic acid, 96.9 % v/v ACN, and 3 % v/v H_2_O. The flow rate was 300 nL/min and LC-MS/MS analysis was carried out on a hybrid quadrupole-TOF mass spectrometer (QSTAR Elite, AB Sciex Inc., Framingham, MA, USA). The focusing potential and ion spray voltage were set to 275 V and 2400 V, respectively. The information-dependent acquisition (IDA) mode of operation was employed in which a survey scan from m/z 400–1200 was acquired followed by collision-induced dissociation (CID) of the four most intense ions.

### Database searching and protein identification

Tandem mass spectra were extracted by AB Sciex Analyst QS 2.0 software. All MS/MS samples were analyzed using Mascot (Matrix Science, London, UK; version 2.2.07). Mascot was configured to search the IPI_bovine database (20120411 version with 30403 entries) for quality control sample of BSA digestion and the IPI_human database (20120411 version with 91464 entries) for experimental samples assuming trypsin digestion. Mascot was searched with a fragment ion mass tolerance of 0.50 Da and a parent ion tolerance of 0.50 Da. Iodoacetamide derivatives of cysteine were specified in Mascot as a fixed modification. S-carbamoylmethylcysteine cyclization of the N-terminus, deamidation of asparagine and glutamine and oxidation of methionine were specified in Mascot as variable modifications.

Scaffold (version Scaffold_3_00_08, Proteome Software Inc., Portland, OR, USA) was used to validate MS/MS-based peptide and protein identifications. Peptide identifications were accepted if they could be established at greater than 90.0 % probability as specified by the Peptide Prophet algorithm [23]. Protein identifications were accepted if they could be established at greater than 99.0 % probability and contained at least two identified peptides. Protein probabilities were assigned by the Protein Prophet algorithm [24]. The false discovery rates at the peptide and protein levels were 1.5 % and 0.1 %, respectively. Proteins that contained similar peptides and could not be differentiated based on MS/MS analysis alone were grouped to satisfy the principles of parsimony.

## Results

**Fig. 1:**
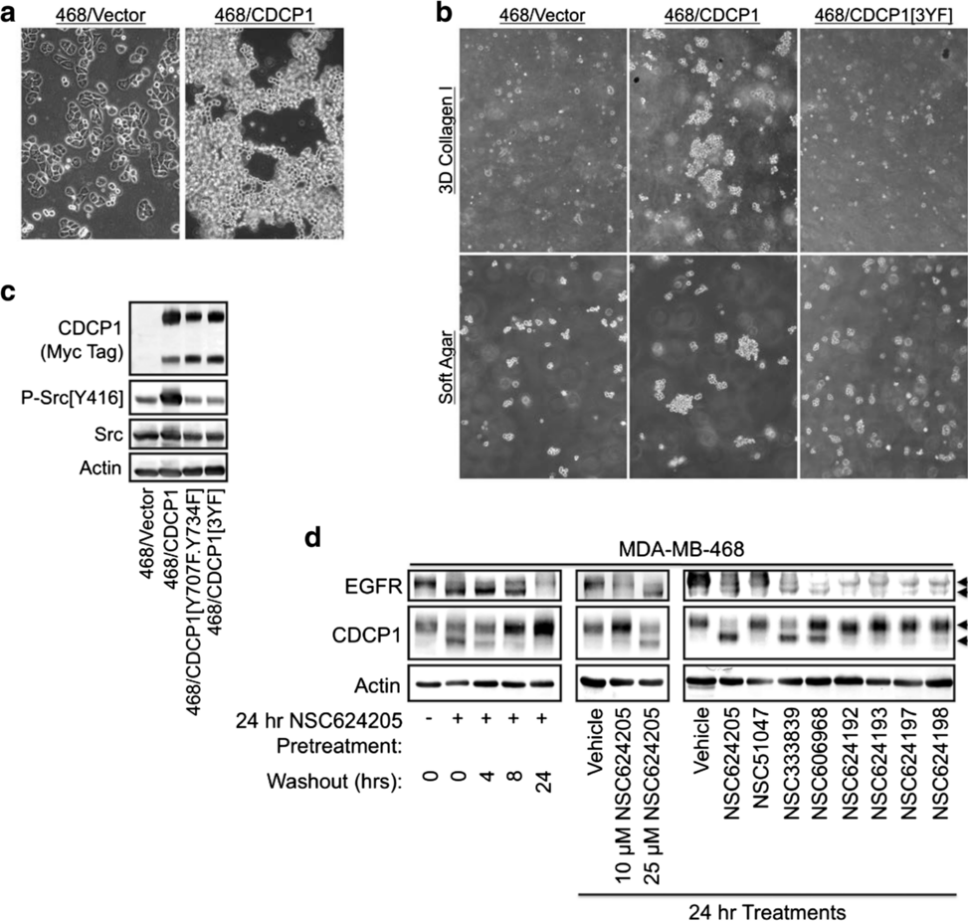
CDCP1 mediates suspension growth of MDA-MB-468 cells. **a** Morphology of the MDA-MB-468 vector control cell line or a line stably overexpressing wild-type CDCP1. CDCP1 induces suspension growth of cell cultures. **b** Comparison of MDA-MB-468 cell lines expressing wild-type CDCP1, or a phospho-defective form of CDCP1 in which tyrosine residues 707, 734, and 762 have been mutated to phenylalanine ([3YF]), grown in either soft agar or collagen I gels. Note that CDCP1-dependent suspension growth requires tyrosine phosphorylation. **c** Immunoblot analysis showing that the MDA-MB-468 cell lines stably express CDCP1 or its mutants at similar levels, but only wild-type CDCP1 increases activating Src phosphorylation above basal levels. **d** Treatment of MDA-MB-468 cells with Disulfide bond Disrupting Agents (DDAs) induces parallel mobility shifts of EGFR and CDCP1 in time- and concentration-dependent manners

Enforced CDCP1 overexpression in the MDA-MB-468 breast cancer cell line induces cell detachment from the substratum, cell proliferation and survival in suspension [12]. Similarly, wild-type CDCP1 induces a detached growth pattern of MDA-MB-468 cells even when they are plated on a substrate suitable for attachment (Fig. 1a). CDCP1 overexpression in this background is also sufficient to drive proliferation and survival of MDA-MB-468 cells in soft agar, as well as collagen I gels (Fig. 1b). Phosphorylation-defective CDCP1 (Y734F) does not induce suspension growth at similar levels of expression (Fig. 1b, c).

This effect of CDCP1 overexpression is not universal, suggesting that MDA-MB-468 cells are particularly susceptible to CDCP1-mediated suspension growth. MDA-MB-468 cells express high EGFR levels in comparison with most other breast cancer cell lines. Thus, crosstalk may exist between EGFR- and CDCP1-dependent signaling. DDAs downregulate EGFR and its family members HER2 and HER3 and prior to EGFR downregulation, a shift in EGFR electrophoretic mobility is evident that parallels EGFR dephosphorylation on the Src kinase phosphorylation site Tyr^845^ [13, 25]. Immunoblot analysis demonstrates that treatment of MDA-MB-468 cells with the DDAs increased the mobility of both EGFR and the full-length form of CDCP1 (Fig. 1d). The CDCP1 and EGFR mobility shifts occurred over a range of concentrations (Fig. 1d, center panel), and were reversible over a similar time course (Fig. 1d, left panel). Further, the patterns of CDCP1 and EGFR mobility were identical across a series of structurally related compounds (Fig. 1d, right panel). Collectively, these results suggest a connection between EGFR and CDCP1 signaling that is suppressed by DDAs, prompting us to investigate potential mechanisms of crosstalk between EGFR and CDCP1.

**Fig. 2:**
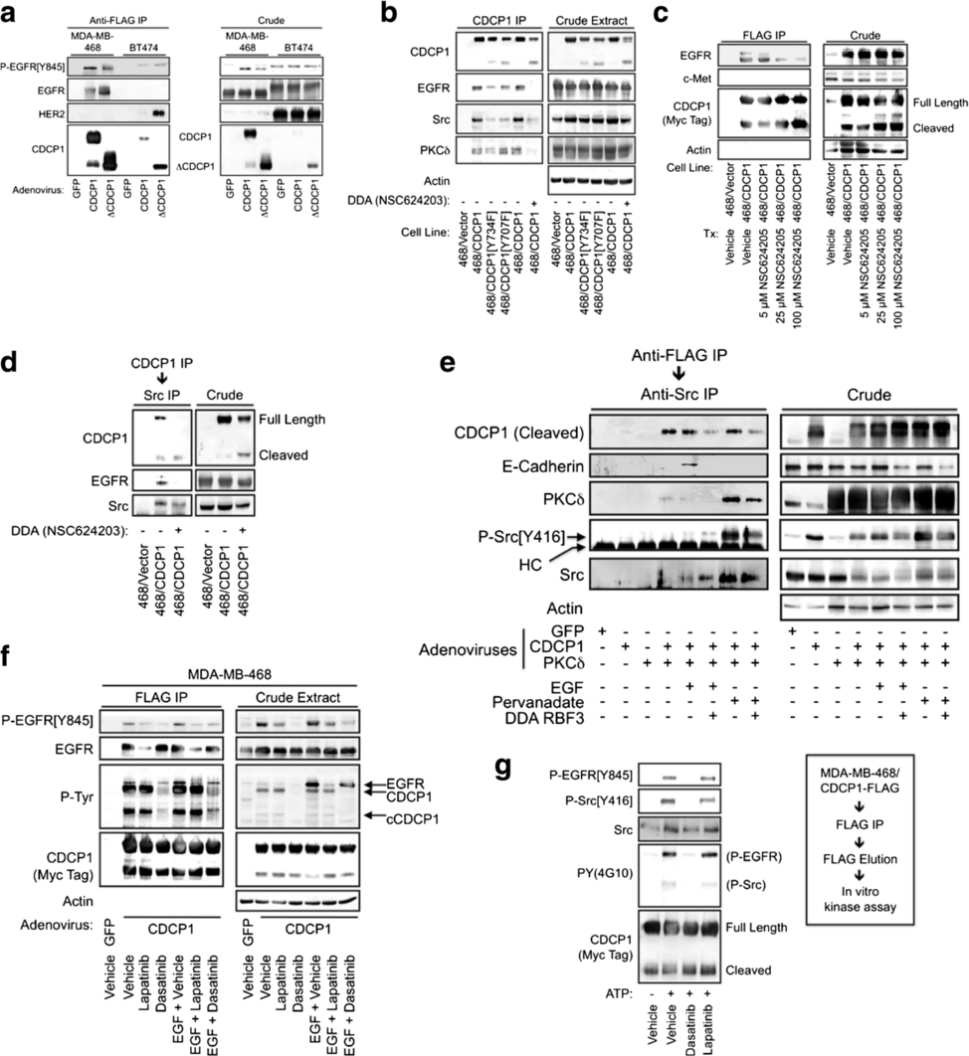
CDCP1 forms ternary complexes with EGFR and Src. **a** Immunoprecipitation of FLAG-tagged CDCP1 from MDA-MB-468 and BT474 cells shows that CDCP1 associates with EGFR and HER2. **b** CDCP1 association with EGFR, Src, and PKCδ are significantly reduced either by mutation of Tyr^734^ to phenylalanine, or by a 24-hour treatment with 20 μM DDA NSC624203. **c** DDA treatment of cells dissociates EGFR/CDCP1 complexes in a concentration-dependent manner. **d** EGFR and Src form ternary complexes, as indicated by sequential CDCP1 and Src co-immunoprecipitation, and complex formation is reduced by treatment of the cells for 24 hours with 20 μM DDA NSC624203. **e** MDA-MB-468 cells transduced with adenoviruses encoding GFP, CDCP1 or PKCδ were stimulated with either 20 μM EGF or 100 μM sodium pervanadate for 20 minutes as indicated. Cell extracts were subjected to sequential anti-FLAG (CDCP1), anti-Src immunoprecipitation. Immunoprecipitates and the corresponding crude cell lysates were analyzed by immunoblot. **f** Lapatinib treatment (20 μM) for 24 hours reduces EGFR association with CDCP1. Dasatinib treatment (100 nM) for 24 hours does not alter EGFR association with CDCP1, but reduces overall tyrosine phosphorylation and EGFR phosphorylation on the Src site, Tyr^845^. In the MDA-MB-468 cell background, complex formation is not significantly altered by stimulation with 10 ng/ml EGF for 20 minutes. **g** Subjection of affinity purified CDCP1-containing complexes to in vitro kinase assays demonstrated ATP-dependent increases in overall tyrosine phosphorylation, EGFR phosphorylation on Tyr^845^ and Src phosphorylation on Tyr^416^. These phosphorylation events were blocked by the addition of 100 nM dasatinib to the kinase assays, but unaffected by 20 μM lapatinib

EGFR co-immunoprecipitated with adenovirally expressed, FLAG-epitope-tagged full-length and cleaved CDCP1 in MDA-MB-468 cells, and the coprecipitated EGFR exhibited tyrosine phosphorylation (Fig. 2a). In contrast, when the HER2-positive BT474 breast cancer cell line was employed in the same experiment, EGFR was barely detectable in association with CDCP1, but HER2 robustly associated with a mimetic of the cleaved form of CDCP1 (ΔCDCP1). DDA NSC624203 disrupted complexes between CDCP1 and EGFR and also dissociated the previously identified CDCP1 binding partners Src [8, 26–28] and PKCδ [7, 9, 26, 29] (Fig. 2b). DDA effects on CDCP1-EGFR complexes occurred in a concentration-dependent manner (Fig. 2c). Endogenous EGFR, but not c-Met, copurified with CDCP1. Since both EGFR and CDCP1 are known substrates for Src-family kinases (SFKs) [8, 26–28, 30–32], we examined whether CDCP1 forms ternary complexes with EGFR and Src. Sequential immunoprecipitations of CDCP1 and Src were performed, followed by immunoblot analysis for all three proteins (Fig. 2d). The results confirmed that these proteins are capable of forming a ternary complex, and that pretreatment of the cells with DDA NSC624203 reduced the association of EGFR, but not Src, with CDCP1. Since Fig. 2b indicated that PKC™ is present in CDCP1-containing complexes, we performed sequential FLAG (CDCP1) and Src immunoprecipitations to determine whether PKC™ is in ternary complexes containing CDCP1 and Src. These experiments demonstrated that in unstimulated cells PKC™ can participate in CDCP1/Src complexes (Fig. 2e). This association was not affected by EGF stimulation, but was reduced by treatment with DDA RBF3. Stimulation of the cells with the tyrosine phosphatase inhibitor pervanadate strongly increased PKC™ association with the complex. This correlated with a dramatic increase in the level of activated Src in the complex, and RBF3 partially overcame the effect of pervanadate. A recent report indicated that PKC™ phosphorylates E-cadherin and blocks E-cadherin mediation of cell-cell adhesion [33]. Interestingly, the presence of E-cadherin in the CDCP1/Src complexes was only apparent upon EGF stimulation, and was not observed with pervanadate stimulation. The observations in Fig. 2e are consistent with a model where CDCP1 blocks cell-cell attachment in part by nucleating complexes containing EGFR, Src, and PKC™ that facilitate PKC™ activation and colocalization with E-cadherin.

Pretreatment of MDA-MB-468 cells with the Src inhibitor dasatinib decreased the degree of EGFR phosphorylation on the Src phosphorylation site Tyr^845^, but did not alter the amount of EGFR that co-immunoprecipitated with CDCP1 (Fig. 2f). Pretreatment of the cells with the EGFR/HER2 inhibitor lapatinib reduced the amount of EGFR that co-immunoprecipitated with CDCP1. Similar results were evident in the presence or absence of cell pretreatment with EGF. Overall, the data in Fig. 2a–f suggest the possibility that CDCP1/EGFR/Src ternary complexes may facilitate CDCP1 and EGFR tyrosine phosphorylation by CDCP1-associated Src. To provide support for this model, CDCP1-containing complexes were immunoprecipitated from MDA-MB-468 cells, eluted with FLAG peptide and the immunoprecipitates split into separate aliquots and subjected to in vitro kinase assays performed in the presence or absence of ATP, the Src inhibitor dasatinib, or the EGFR/HER2 inhibitor lapatinib. CDCP1-associated Src became phosphorylated on activation loop site Tyr^416^; activated Src, and phosphorylated EGFR on the transactivation site Tyr^845^ because both events were blocked by dasatinib, but unaffected by lapatinib (Fig. 2g). Hence, CDCP1 may play a role both in Src activation, as previously shown [8], and also in promoting EGFR transactivation by Src.

**Fig. 3:**
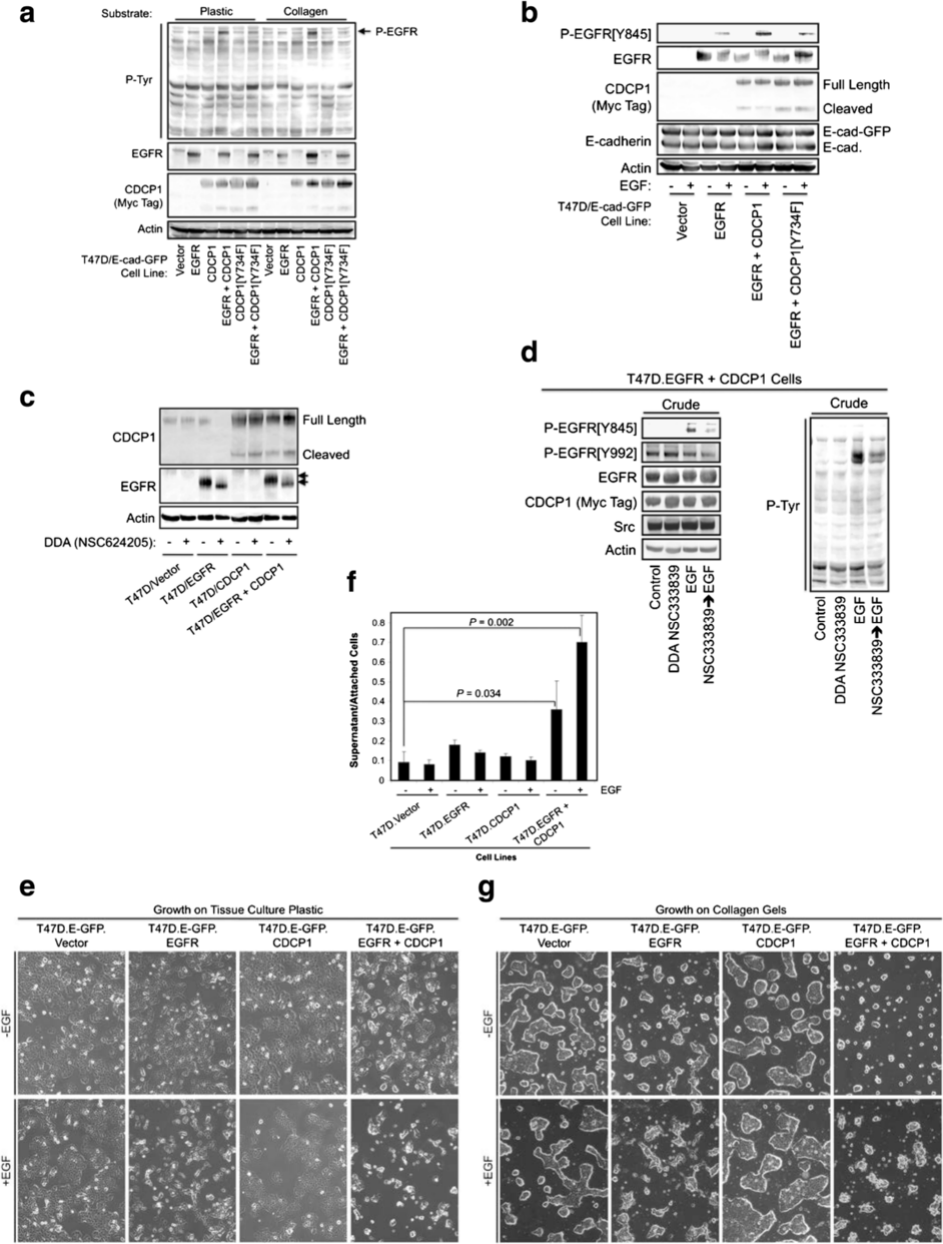
CDCP1 and EGFR cooperate to induce cell detachment from the substratum and to disrupt adherens junctions. **a** Immunoblot analysis of T47D cell lines stably expressing the indicated combinations of EGFR, CDCP1, and the Y734F mutant CDCP1. Coexpression of wild-type CDCP1 and EGFR results in a tyrosine-phosphorylated band that comigrates with EGFR. Similar results were observed whether the cells are plated on tissue culture plastic or type I collagen. **b** Coexpression of CDCP1 and EGFR result in enhanced EGF-dependent EGFR phosphorylation on Tyr^845^. This effect was blunted if wild-type CDCP1 was replaced with the Y734F mutant. **c** Treatment with DDA NSC624205 (20 μM) for 24 hours induces an electrophoretic mobility shift of EGFR, but not CDCP1, in the T47D cell background. **d** T47D cells stably expressing EGFR and CDCP1 were treated for 24 hours with vehicle or 20 μM DDA NSC333839 and stimulated for 20 minutes with or without 10 ng/ml EGF and analyzed by immunoblot. **e** The indicated cell lines were plated onto tissue culture plastic and treated with or without 10 ng/ml EGF for 24 hours and photographed. **f** Cells grown and treated as in Fig. 3b were gently pipetted with the culture medium and the detached suspension cells, and the cells remaining attached to the plates were counted. Results were plotted as the fraction of cells that were detached in triplicate determinations. Error bars represent standard deviation (SD). **g** Photomicrographs of the indicated cell lines treated as in Fig. 3e, but grown on the surface of collagen I gels. Note that EGFR and CDCP1 coexpression and EGF stimulation cooperate to induce suspension growth

These data (Figs. 1 and 2) suggested that the reason that CDCP1 overexpression drives suspension growth of MDA-MB-468 cells is that these cells express high levels of EGFR. This hypothesis was tested by examining whether enforced coexpression of EGFR and CDCP1 in a cell line with low basal levels of EGFR and CDCP1 is sufficient to disrupt cell-cell and cell-substratum attachment. The development and characterization of T47D breast cancer cells engineered to stably express a green fluorescent protein (GFP)-E-cadherin fusion protein for monitoring E-cadherin intracellular localization was previously described [16]. Derivatives of this T47D.E-cad-GFP line were constructed with stable, forced expression of EGFR, CDCP1, or both proteins (Fig. 3a). EGF stimulation of the resulting lines induced EGFR phosphorylation on Tyr^845^ in the EGFR-overexpressing lines (Fig. 3b). Coexpression of wild-type CDCP1 enhanced Tyr^845^ phosphorylation of EGFR, but the CDCP1[Y734F] mutant lacking the Src binding site did not. Treatment of these cell lines with DDA NSC624205 induced an EGFR mobility shift (Fig. 3c) as observed in Fig. 2, and NSC624205 reduced EGF-induced EGFR phosphorylation on Tyr^845^ (Fig. 3d), but a CDCP1 mobility shift was not observed under these conditions. Examination of the T47D.E-cad-GFP lines growing on tissue culture plastic revealed a somewhat similar morphology in the absence of EGF, with the exception that the EGFR/CDCP1 coexpressing line tended to contain loosely attached clusters of cells (Fig. 3e). Treatment of the cell lines with EGF for 24 hours caused the EGFR-expressing line to grow as single attached cells rather than in colonies. Coexpression of CDCP1 with EGFR further enhanced the effects of EGF and resulted in cultures in which most of the cells were either loosely attached or grew in suspension. Gentle pipetting of the cultures and counting the cells in suspension and the cells remaining attached to the plates showed a statistically significant cooperativity between EGFR, CDCP1, and EGF treatment in inducing cell detachment (Fig. 3f). Cells grown on collagen gels rather than on tissue culture plastic exhibited similar trends toward a more detached growth pattern with the expression of EGFR and CDCP1, and with EGF stimulation (Fig. 3g).

**Fig. 4:**
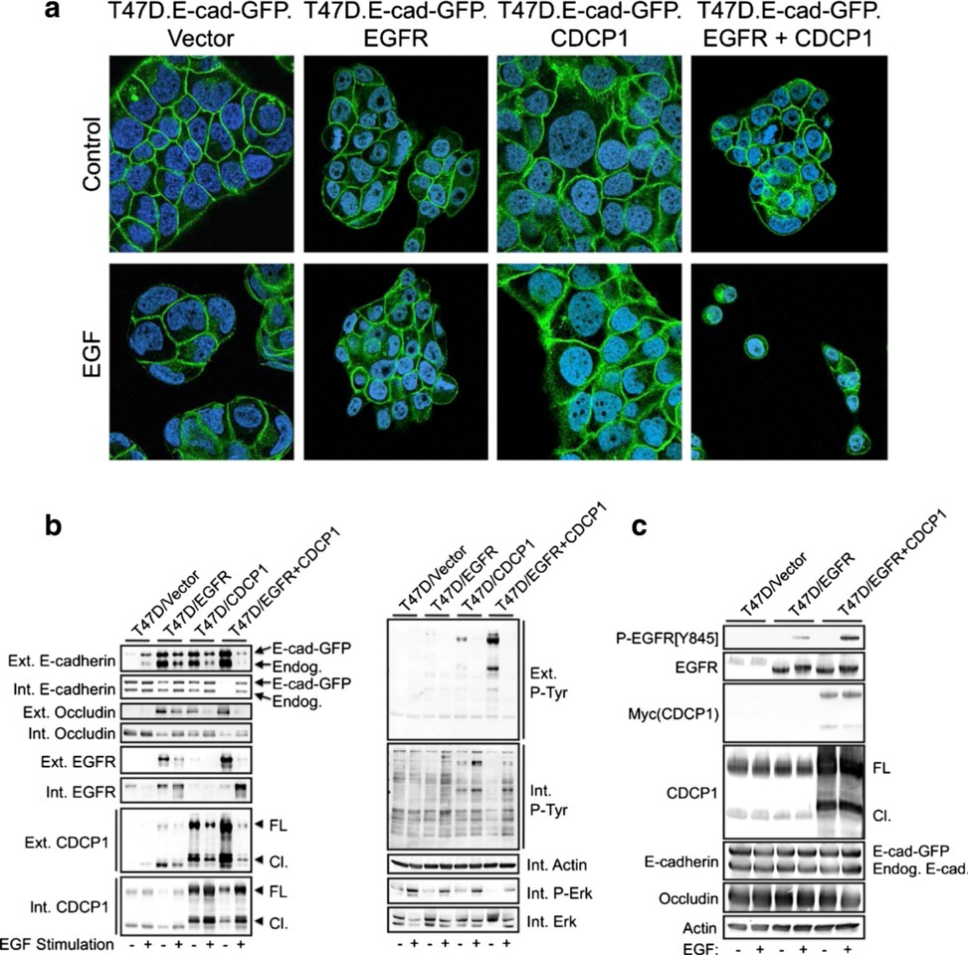
CDCP1, EGFR, and EGF cooperate to induce E-cadherin internalization. **a** T47D cells stably expressing an E-cadherin-GFP fusion protein and either EGFR, CDCP1, or EGFR + CDCP1, or the corresponding vector control line were plated on glass coverslips coated with collagen I and treated for 24 hours with or without 10 ng/ml EGF. Intracellular E-cadherin-GFP localization was imaged by confocal immunofluorescence microscopy. **b** The indicated T47D cell lines were stimulated with or without 20 ng/ml EGF for 24 hours and subjected to cell surface biotinylation. Biotinylated proteins (external, *Ext*.) were isolated by streptavidin-agarose chromatography and unbound proteins were collected as non-biotinylated proteins (internal, *Int*.). Protein fractions were analyzed by immunoblot. **c** The indicated cell lines treated as in Fig. 4b were extracted with 1 % Triton X100 and analyzed by immunoblot

An interesting observation from Fig. 3e and g was that cultures of cells overexpressing EGFR, and to a greater extent EGFR + CDCP1, contained numerous single cells growing either attached or in suspension, suggesting that in addition to suppressing cell-substratum adhesion, CDCP1 and EGFR also cooperate to disrupt cell-cell adhesion. Examining E-cadherin-GFP localization in each line treated with or without EGF showed that the vector control cell line exhibited sharp, well-defined E-cadherin localization at cell-cell junctions (Fig. 4a). E-cadherin was also localized to the cell periphery around the outer edges of each colony. EGFR overexpression, CDCP1 overexpression, and EGF treatment individually increased the diffuseness of E-cadherin localization at cell-cell junctions, caused more cytoplasmic E-cadherin localization, and induced loss of E-cadherin localization at some sites along colony peripheries. The most dramatic alteration in E-cadherin localization and function occurred in cells that overexpressed both EGFR and CDCP1 and were stimulated with EGF. Under these conditions, cultures consisted of single cells or small clusters of cells that were loosely attached to the substratum, and E-cadherin was largely internalized to the cytoplasm in many of these cells.

As biochemical confirmation of the changes in E-cadherin localization observed by fluorescence microscopy, we performed surface biotinylation experiments. E-cadherin labeling was not observed in the control cells and labeling was slightly increased by EGF stimulation (Fig. 4b). Lack of E-cadherin surface labeling while the protein is at cell-cell junctions was previously observed in Madin-Darby Canine Kidney (MDCK) monolayers [34], and it was subsequently shown that stimulation of MDCK cells with hepatocyte growth factor (HGF) increases E-cadherin accessibility [35]. Since T47D cells are relatively well differentiated and have served as a model system for studying tight junction proteins [36, 37], the simplest interpretation is that in T47D cells E-cadherin is at cell-cell junctions, but largely inaccessible from the apical surface.

Enforced expression of either EGFR or CDCP1 dramatically increased E-cadherin surface labeling, while EGF stimulation induced partial E-cadherin internalization in both cell lines. In T47D cells coexpressing EGFR and CDCP1, E-cadherin was almost completely external. EGF treatment induced a striking internalization of E-cadherin. Similar patterns of redistribution were observed with the tight junction protein Occludin, EGFR, and CDCP1. The T47D/EGFR + CDCP1 cells showed high levels of tyrosine phosphorylation of cell surface proteins under control conditions, while after EGF treatment, tyrosine phosphorylated proteins were observed primarily in the internal fraction. These effects are not due to global changes in EGF signaling since EGF stimulated Erk phosphorylation similarly in all four cell lines.

Alternate interpretations are that rather than inducing alterations in protein accessibility, these transmembrane proteins could relocalize to detergent-insoluble domains, or the observations could result from changes in the overall levels of these proteins. However, when whole cells are extracted using similar conditions (1 % Triton X100 whole cell lysates), no differences in E-cadherin levels were observed between cell lines with or without EGF treatment (Fig. 4c). Thus, the simplest interpretation that is consistent with both the E-cadherin fluorescence and surface biotinylation results is that the control T47D cells possess strong cell-cell junctions that limit the access of antibodies or surface biotinylation probes to E-cadherin. However, elevated expression of EGFR or CDCP1 increases E-cadherin accessibility to labeling, but does not induce E-cadherin internalization. EGF treatment in conjunction with EGFR and CDCP1 coexpression induce E-cadherin and Occludin internalization, which coincides with loss of cell-cell adhesion. Together, these observations suggest that changes in E-cadherin localization through cooperative effects between EGFR and CDCP1 may explain how these proteins contribute to decreased cell-cell and cell-substratum adhesion. This may in turn account for the relationship between CDCP1 or EGFR expression in cancer and increased invasiveness, elevated cancer dissemination, and poor patient outcome [38–40].

**Fig. 5:**
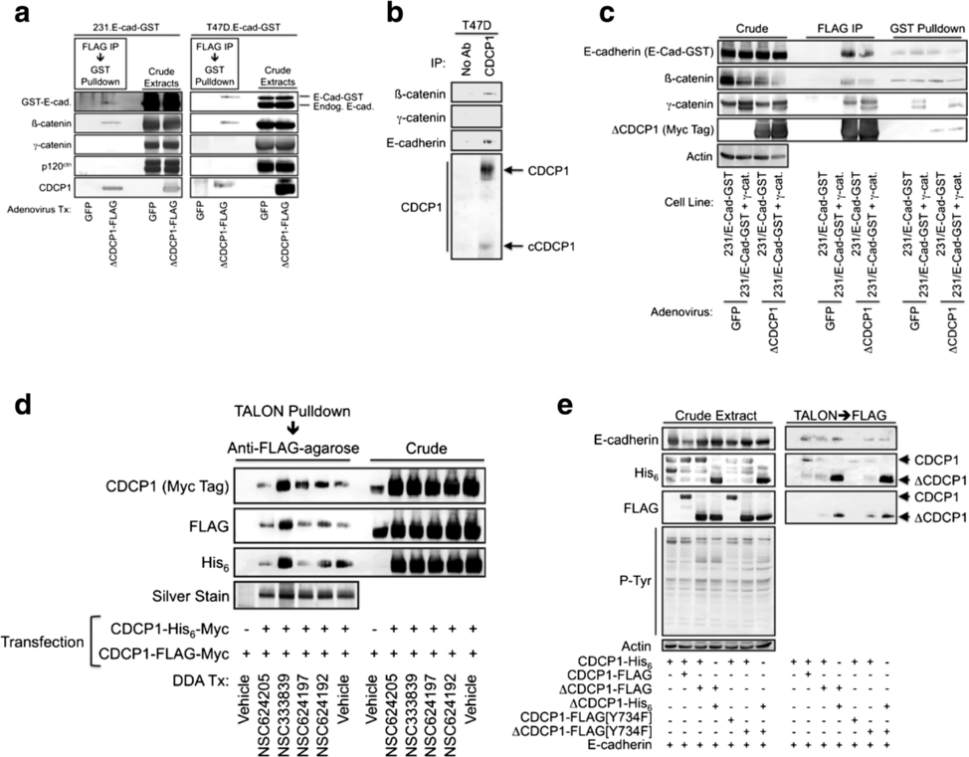
Characterization of E-cadherin/CDCP1-containing protein complexes. **a** MDA-MB-231 or T47D cell lines stably expressing an E-cadherin-glutathione S-transferase (E-cad-GST) fusion protein were transduced with an adenovirus encoding ΔCDCP1-FLAG or a control adenovirus encoding GFP. Cell extracts were subjected to sequential affinity purification using anti-FLAG agarose followed by glutathione-agarose. **b** T47D cell extracts were immunoprecipitated with a CDCP1 antibody and the immunoprecipitates were analyzed by immunoblot with the indicated antibodies. **c** The indicated stable cell lines were transduced with adenoviruses encoding GFP (control) or ΔCDCP1-FLAG and cell extracts were subjected to affinity purification with either anti-FLAG agarose or glutathione agarose. Affinity-purified proteins were analyzed by immunoblot. **d** HEK 293 cells were transiently transfected with the indicated tagged CDCP1 constructs and treated with the specified DDAs or vehicle. CDCP1 dimerization was monitored by TALON pulldown of His_6_-tagged proteins followed by purification with anti-FLAG agarose and immunoblot analysis of the sequentially affinity-purified material. **e** HEK 293 cells were transiently transfected with the indicated plasmid constructs and cell extracts were affinity purified sequentially using TALON resin and anti-FLAG agarose. The affinity-purified material was analyzed by immunoblot

The cleaved form of CDCP1 and a deletion mutant, ΔCDCP1, which mimics the cleaved form of CDCP1, associate with E-cadherin [16]. In order to further explore the composition of these complexes, affinity purification of E-cadherin/ΔCDCP1 complexes employed T47D and MDA-MB-231 breast cancer cells stably expressing an E-cadherin-glutathione S-transferase (GST) fusion protein and an adenoviral vector encoding FLAG-tagged ΔCDCP1. Sequential anti-FLAG agarose and glutathione-agarose purifications revealed that E-cadherin/ΔCDCP1 complexed with the endogenous E-cadherin binding partner β-catenin, but not γ-catenin or p120 catenin (Fig. 5a). Immunoprecipitation of endogenous CDCP1 from T47D cells also showed association of both E-cadherin and β-catenin (Fig. 5b). γ-catenin interacted with E-cadherin in T47D cells, but not in MDA-MB-231 cells, due to lower γ-catenin levels in the latter cell line [16]. One interpretation of this observation is that the apparent lack of γ-catenin in E-cadherin/ΔCDCP1 complexes may be due to γ-catenin levels below the detection limit due to the two-step rather than a single-step affinity purification. Alternately, γ-catenin-containing complexes may be less stable than the corresponding β-catenin complexes. To address this point, FLAG-tagged ΔCDCP1 was expressed in MDA-MB-231-derived cell lines that express an E-cadherin-GST fusion protein in the presence or absence of γ-catenin coexpression using an adenoviral vector. E-cadherin or ΔCDCP1 were isolated using glutathione-agarose or anti-FLAG agarose, respectively. Analysis of the affinity-purified complexes revealed that E-cadherin, β-catenin, and γ-catenin coprecipitate with ΔCDCP1 with or without γ-catenin overexpression (Fig. 5c). In contrast, γ-catenin was detected in E-cadherin-GST pulldowns only in the context of γ-catenin overexpression.

CDCP1 may exist as a dimer [28]. Results obtained using differentially epitope-tagged CDCP1 constructs confirm CDCP1 dimerization (Fig. 5d). Dimer formation was not reduced by DDAs, indicating that the disruption of CDCP1/EGFR/Src complexes observed in Fig. 2b was not due to blockade of CDCP1 dimerization. In transient transfection experiments, the most robust dimerization was observed with ΔCDCP1 as compared with CDCP1/CDCP1 homodimers or CDCP1/ΔCDCP1 heterodimers (Fig. 5e). E-cadherin was detected in association with CDCP1 dimers, and appeared to be present in all three classes of CDCP1 dimers despite previous findings that E-cadherin associates preferentially with cleaved CDCP1 or the cleaved CDCP1 mimetic, ΔCDCP1 [16]. One explanation for this discrepancy is that at least some processed CDCP1 is constitutively present due to cleavage of the full-length form. Consistent with this, the short form of CDCP1 is detectable in the sequentially affinity-purified samples obtained from cells transfected with full-length CDCP1. This effect may be amplified by the apparent enhanced ability of the short form of CDCP1 to dimerize. It was also unexpected that similar levels of E-cadherin would be copurified when very different levels of the various dimers were isolated. This could indicate that E-cadherin associates with CDCP1 dimers indirectly and is limited by the availability of an intermediary protein. Overall, the findings in Fig. 5 are consistent with a model in which CDCP1 exists in one or more protein complexes that contain E-cadherin, and that CDCP1 dimerization may be enhanced by its cleavage.

**Fig. 6:**
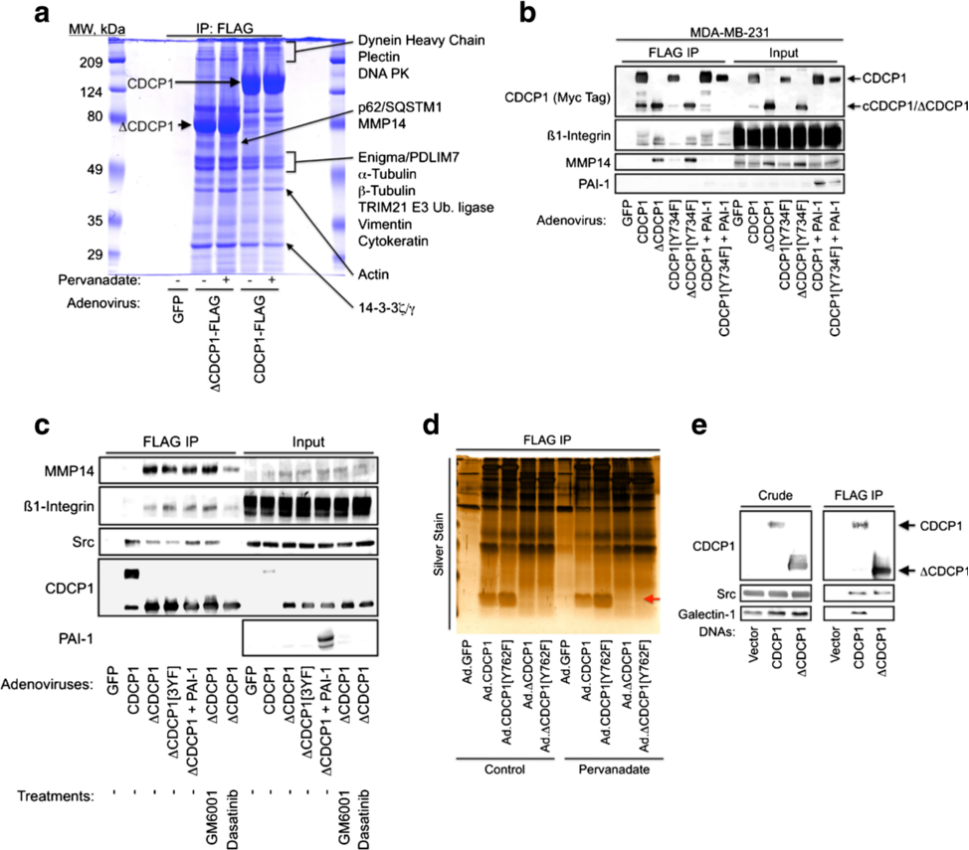
Characterization of CDCP1-containing protein complexes reveals that MMP14 preferentially associates with cleaved CDCP1, while Galectin 1 only binds to full-length CDCP1. **a** Coomassie-stained SDS-PAGE gel of proteins affinity purified from MDA-MB-231 cells transduced with FLAG-tagged ΔCDCP1 or CDCP1 and treated for 15 minutes with or without the tyrosine phosphatase inhibitor sodium pervanadate (100 μM) to elevate overall tyrosine phosphorylation. Protein bands were excised and identified by mass spectrometry as described in the “Methods” section. **b** Extracts from MDA-MB-231 cells transduced with the indicated adenoviral vectors were affinity purified with anti-FLAG agarose to isolate CDCP1-containing complexes and the purified material or corresponding crude lysates were analyzed by immunoblot. **c** Study performed as in Fig. 5b except that cells were treated for 24 hours with either vehicle, 20 μM of the matrix metalloproteinase inhibitor GM6001 or 100 nM of the Src-family kinase inhibitor dasatinib. **d** Silver-stained SDS-PAGE gel of proteins isolated by anti-FLAG affinity purification from MDA-MB-231 cells transduced with the indicated adenoviruses and treated for 15 minutes with or without 100 μM sodium pervanadate. A low-molecular-mass protein was observed (*red arrow*) that was present when full-length forms of CDCP1 were expressed, but not when cleaved forms of CDCP1 (ΔCDCP1) were expressed. **e** HEK 293 cells were transiently transfected with the indicated vectors and CDCP1 or ΔCDCP1-containing complexes were isolated with anti-FLAG agarose and analyzed by immunoblot

The ability of EGFR and CDCP1 to cooperate in Src activation resulting in cell-cell and cell-substratum detachment may reflect the ability of these proteins to induce E-cadherin relocalization from the plasma membrane to cytoplasmic vesicles. This hypothesis is consistent with previous reports demonstrating the ability of EGFR- and Src-dependent signaling to disrupt cadherin function [41–45]. CDCP1 harbors no known catalytic activities and thus mediates its anti-adhesive actions via protein-protein interactions. Therefore, affinity purification of CDCP1 complexes was followed by analysis of the multiprotein assemblies using mass spectrometry to gain insight into how CDCP1 actions are carried out. Under conditions where no proteins were detected in control affinity purifications from cells transduced with an adenovirus encoding GFP, several proteins were identified which copurified with either CDCP1 or ΔCDCP1. A gel representative of multiple experiments shows that many of the same protein bands were observed in the CDCP1 and ΔCDCP1 affinity purifications (Fig. 6a). The proteins listed were identified in the indicated bands or gel slices by mass spectrometry (proteomics results are presented in more detail in Additional file 1: Table S1, Additional file 2: Table S2 and Additional file 3: Figure S1). One band was present selectively in ΔCDCP1 complexes and found to contain the adaptor protein p62/SQSTM1 that is involved in targeted autophagy and proteasomal degradation (reviewed in [46, 47]), and the matrix metalloproteinase MMP14 that promotes cancer metastasis by permitting cancer cell survival and proliferation through extracellular matrix degradation [48–50]. Since CDCP1 is essential for MMP14 trafficking to invadopodia to degrade the extracellular matrix during cell invasion [14], adenoviruses encoding the cleaved and full-length forms of CDCP1 were used to show that MMP14 selectively associates with ΔCDCP1 but not the full-length protein (Fig. 6b). The small amounts of MMP14 observed in association with full-length CDCP1 were apparently a result of CDCP1 cleavage because coexpression of the serine protease inhibitor PAI-1 blocked CDCP1 processing and rendered MMP14 pulldown undetectable in cells expressing almost exclusively full-length CDCP1. Association of MMP14 was not affected by mutating ΔCDCP1 at the major Src binding site, Tyr^734^. E-cadherin [16] and β1-integrin [11] bind selectively with the short form of CDCP1, while Src associates with CDCP1 irrespective of proteolytic processing. As expected, the pattern of MMP14 association with CDCP1 matched that observed with β1-integrin, but not that seen with Src (Fig. 6c).

**Fig. 7:**
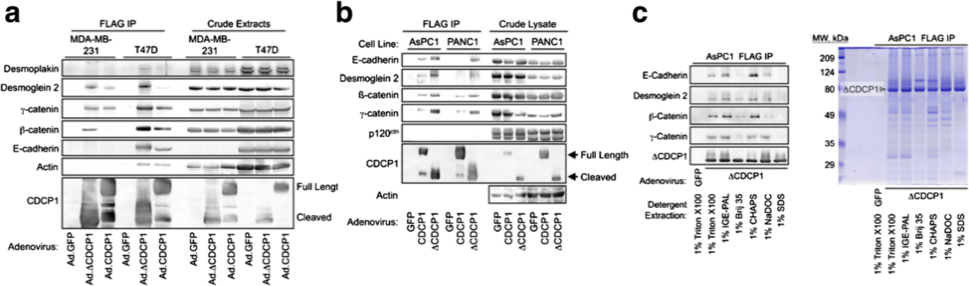
CDCP1 complexes are similar between breast and pancreatic cancer cell lines and are stable in a variety of detergents. **a** MDA-MB-231 or T47D cells were transduced with the indicated adenoviral vectors, CDCP1-containing complexes were isolated with anti-FLAG agarose, and the presence of components of adherens and desmosomal junctions was assessed by immunoblot analysis. **b** This study was carried out similarly to the study in Fig. 6a, except that the pancreatic cancer cell lines AsPC1 or PANC1 were used. **c** AsPC1 cells were transduced with control (GFP) or ΔCDCP1 adenoviruses, the cells were extracted with the indicated detergents, and ΔCDCP1-associated proteins were examined by immunoblot (*left panel*) or total protein levels were assessed by Coomassie staining of the same samples resolved by SDS-PAGE (*right panel*)

Efforts to identify proteins that associate with the full-length, but not cleaved CDCP1 revealed a low-molecular-weight protein with this selectivity (Fig. 6d, red arrow). Analysis of this band by mass spectrometry identified it as the lectin Galectin 1. Galectin 1 specificity for full-length CDCP1 was verified in experiments showing that transiently expressed CDCP1, but not ΔCDCP1, pulled down endogenous Galectin 1, while both forms of CDCP1 pulled down Src equivalently (Fig. 6e). However, the results in Fig. 6a indicate that most CDCP1-associated proteins bind full-length and cleaved forms similarly. Immunoblot analyses of CDCP1/ΔCDCP1 immunoprecipitates were performed to support the identifications made by mass spectrometry and to further characterize these complexes. Interestingly, not only did ΔCDCP1 associate with E-cadherin, β-catenin, γ-catenin, and desmoplakin in cancer cell lines as expected based on the results in Figs. 5 and 6 and a previous study [16], but ΔCDCP1 also associated with the desmosomal cadherin desmoglein 2 (Fig. 7a). Similar results were obtained using AsPC1 and PANC1 pancreatic cancer cell lines (Fig. 7b). The CDCP1-containing multiprotein complexes were evident after cell extraction with several detergents, including 1 % each of Triton X100, IGE-PAL, CHAPS, and sodium deoxycholate, but were less apparent when extractions were performed with 1 % Brij 35 or sodium dodecylsulfate (Fig. 7c). Together, these results suggest that CDCP1 associates either directly or indirectly with numerous proteins present in adherens junctions and desmosomal junctions, consistent with the findings in Figs. 1, 3, and 4, which show that CDCP1 overexpression can contribute to the disruption of cell-cell junctions and cell-substratum contacts.

**Fig. 8:**
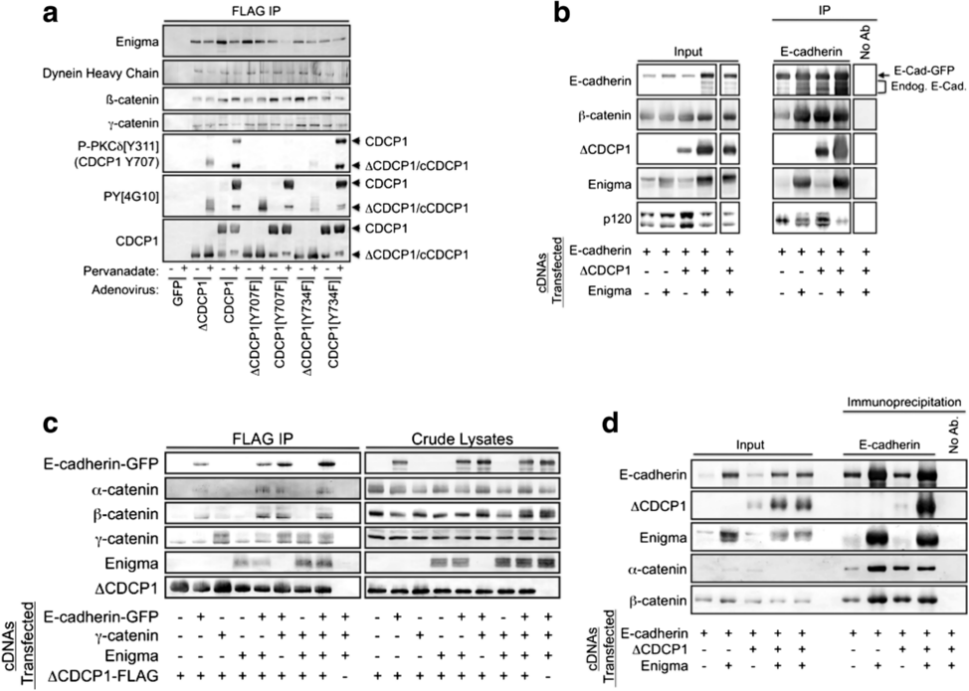
Enigma/PDLIM7 as a novel CDCP1-associated protein. **a** MDA-MB-231 cells were transduced with the indicated recombinant adenoviruses, then treated for 15 minutes with 100 μM sodium pervanadate just before cell lysates were prepared. The various forms of CDCP1 were isolated by anti-FLAG affinity chromatography and CDCP1-associated proteins were identified by immunoblot. Note that the phospho-PKCδ[Y311] antibody cross-reacts with phosphorylated Tyr^707^ of CDCP1 and that staining was absent when the Y707F mutant of CDCP1 was employed. **b** HEK 293 cells were transfected with the indicated constructs, cell lysates were immunoprecipitated with an E-cadherin antibody, and the immunoprecipitates were analyzed by immunoblot. **c** HEK 293 cells were transfected with the indicated constructs, cell lysates were immunoprecipitated with anti-FLAG agarose, and the immunoprecipitates were analyzed by immunoblot. **d** HEK 293 cells were transfected with the indicated constructs, cell lysates were immunoprecipitated with an E-cadherin antibody, and the immunoprecipitates were analyzed by immunoblot

The CDCP1-associated protein Enigma/PDLIM7 identified in our mass spectrometry studies was selected for further study for three reasons. First, the function of Enigma has not been investigated extensively in epithelial cell lines. Second, studies in the heart indicate Enigma plays a key role in regulating cell signaling and the actinomyosin cytoskeleton [51–54]. Third, Enigma contains an N-terminal PDZ domain and three C-terminal LIM domains, and thus may function as a molecular scaffold to coordinate cell-cell and cell-substratum adhesion. Enigma associated with both full-length and cleaved CDCP1 and this association was not strongly modulated by tyrosine phosphorylation (Fig. 8a). Ectopically expressed E-cadherin, Enigma, and ΔCDCP1 mutually elevated their expression levels (Fig. 8b). Enigma increased the levels of β-catenin and ΔCDCP1 in E-cadherin complexes, while reducing the levels of p120 catenin in E-cadherin complexes. These effects are likely due to differences in the overall expression of the respective proteins. E-cadherin was coexpressed with its major binding partners α-catenin, β-catenin, and γ-catenin in various combinations. The resulting complexes with ΔCDCP1 were analyzed by immunoprecipitation followed by immunoblotting and confirmed that Enigma was also present in these complexes, but did not significantly alter the levels of any of the other proteins in the complex (Fig. 8c). This observation suggests that ΔCDCP1 interacts with Enigma and E-cadherin through distinct, non-overlapping sites, or that ΔCDCP1 associates with Enigma and E-cadherin through different protein complexes. Reciprocal immunoprecipitations with E-cadherin antibodies produced similar results (Fig. 8d), although coexpression of Enigma with E-cadherin increased the amounts of E-cadherin, ΔCDCP1, and β-catenin in the immunoprecipitates. Together, the findings in Figs. 7 and 8 show that CDCP1 associates with several proteins involved in cell-cell and cell-substratum adhesion, identify novel CDCP1 binding partners such as Enigma and Galectin 1, illustrate that CDCP1 associates with MMP14 and Galectin 1 in a manner dependent on the state of CDCP1 proteolytic processing, and identify Enigma as a protein present in CDCP1- and E-cadherin-containing protein complexes.

## Discussion

### Cell detachment from the substratum and from neighboring cells as a key event in cancer dissemination

Cancer cell de-adhesion from the primary tumor, either in the form of individual cells or cell clusters, is recognized as a key event in metastatic spread. Clarification of the mechanisms that regulate this process is important for the development of approaches to effectively prevent metastasis by either blocking cell detachment or restoring anoikis. Dissemination of individual epithelial cancer cells necessitates disruption of cell-substratum adhesion, which involves focal contacts and hemi-desmosomes, and dissociation of cell-cell adherens, tight, and desmosomal junctions. CDCP1 associates with proteins involved in cell-cell and cell-substratum adhesion, including N- and P-cadherins and syndecans [3], E-cadherin [16], and β1-integrin [11] and CDCP1 binds to signaling proteins with known roles in the control of cell motility and cell-cell adhesion including EGFR [41, 55], Src-family kinases [3, 8] and Akt [10, 11]. Thus, CDCP1 represents a promising candidate for gaining further insights into the mechanisms that contribute to metastasis. The proteomics results presented here (Additional file 1: Table S1 and Additional file 2: Table S2) expand the number of known CDCP1-associated proteins and indicate that many are involved in cell-cell or cell-substratum adhesion. These results indicate that CDCP1 interacts not only with cadherins that participate in adherens junctions but also with the desmosomal cadherin desmoglein 2 (Fig. 7a-c). CDCP1 complexes also contain β-catenin, characteristic of adherens junctions, and γ-catenin/plakoglobin, a component of desmosomal junctions.

An emerging theme from these studies [16] and those of others [11] is that the state of CDCP1 proteolytic processing influences its spectrum of protein-protein interactions. Cleaved CDCP1 associates preferentially with E-cadherin [16], desmoglein 2 (Fig. 7a-c), MMP14 (Fig. 6a-c), and β1-integrin ([11] and Fig. 6b, c). In contrast, Galectin 1 exclusively associates with the full-length form of CDCP1 (Fig. 6d). Since Galectin 1 is a lectin, this suggests that the N-terminal-most region of CDCP1, which is lost in the cleaved form, contains carbohydrate moieties recognized by Galectin 1. Further studies will be required to assess the biological significance of the CDCP1/Galectin 1 association and how this interaction influences CDCP1-dependent signaling.

### CDCP1 in the transactivation of EGFR by Src

**Fig. 9:**
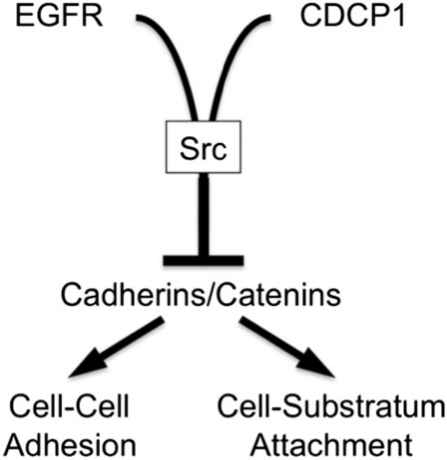
Model for CDCP1 influences on cell-cell and substratum adhesion through a variety of mechanisms. We propose that CDCP1 participates in complexes with EGFR and Src leading to Src activation and EGFR transactivation, and that CDCP1 also participates in complexes that contain cell adhesion proteins such as cadherins and catenins. EGFR and Src have been established to reduce E-cadherin-mediated cell-cell adhesion and to induce E-cadherin internalization into cytoplasmic vesicles. Therefore, CDCP1 may abrogate cadherin adhesive function through the assembly of “anti-adhesive” complexes

EGFR transactivation by Src plays an important role in the cooperation between these two oncogenes during cell transformation by providing an EGFR “gain of function.” For example, Src phosphorylates EGFR on tyrosine residues that are distinct from EGFR autophosphorylation sites, and these transactivation sites couple to downstream PI3K activation more effectively than the EGFR autophosphorylation sites [56]. Overall, these results suggest the simplified model in Fig. 9 that CDCP1 facilitates the formation of a ternary complex involving EGFR and Src and that this complex results in Src activation, and Src-dependent CDCP1 phosphorylation and EGFR transactivation.

### CDCP1 as a protein-protein interaction hub

Proteomics analyses indicate that CDCP1 multiprotein assemblies include proteins associated either directly or indirectly with the microfilament, intermediate filament, or microtubule cytoskeletal networks. These complexes, furthermore, contain proteins that mediate cell-cell and cell-substratum adhesion. Collectively, these results suggest that CDCP1 participates in the formation of protein complexes that control cell adhesion. CDCP1 complexes contain cell adhesion proteins that couple with cytoskeletal proteins [3, 16]. Such CDCP1-associated structural proteins were absent in control samples in which epitope-tagged CDCP1 was not expressed (e.g. Figs. 6a and 7c) indicating they are not the result of nonspecific interactions. CDCP1 also localizes to invadopodia, moreover, invadopodia contain multiple cytoskeletal elements including actin, tubulin, keratin, and vimentin [57–59]. This is consistent with the detection of these proteins and their binding partners in CDCP1 complexes in MDA-MB-231 cells (Additional file 1: Table S1 and Additional file 2: Table S2), which are commonly used to study invadopodia [60–62]. In addition, CDCP1 complexes contain other adaptor proteins including Enigma, dynein, and plectin, which have multiple protein-protein interaction domains and function as molecular scaffolds [53, 63–66].

## Conclusions

These results indicate a new function for CDCP1 in facilitating Src-dependent EGFR transactivation. Ternary complex formation among CDCP1, EGFR, and Src is paralleled by disruption of cell-cell and cell-substratum adhesion in human breast carcinoma cells, coincident with relocalization of E-cadherin from cell-cell junctions to cytoplasmic vesicles. The current findings suggest a mechanism whereby CDCP1, EGFR, and Src cooperate to induce cancer aggressiveness and dissemination, and may provide a rationale for therapeutic targeting of these proteins in combination. Alternatively, since DDAs disrupt the CDCP1/EGFR/Src ternary complex, in addition to effectively inhibiting the growth of HER2-positive malignancies [13], DDAs may be useful for the treatment of breast tumors that overexpress EGFR, or for blocking metastasis driven by signaling via the CDCP1/EGFR/Src ternary complex. These possibilities are currently under investigation.

## Abbreviations

CDCP1(Y734F), CDCP1 with a mutation of tyrosine 734 to phenylalanine; CDCP1, CUB domain-containing protein 1; DDAs, Disulfide bond Disrupting Agents; EGFR, epidermal growth factor receptor; GFP, green fluorescent protein; GST, glutathione S-transferase; HER2, human epidermal growth factor receptor 2; IDA, information-dependent acquisition; MMP14, matrix metalloproteinase 14; PAI-1, plasminogen activator inhibtor-1; SFKs, Src-family kinases; ΔCDCP1, mimetic of the cleaved form of CDCP1

## Additional files

Additional file 1: Report of peptides identified by tandem MS. (PDF 177 kb) Additional file 2: Summary of proteins identified by MS. (PDF 20 kb) Additional file 3: Peptide sequence coverage. (PDF 1263 kb)
