# Supplementary material for: CUB domain-containing protein 1 and the epidermal growth factor receptor cooperate to induce cell detachment
Source: Breast Cancer Res. 2016 Aug 5;18:80. doi: 10.1186/s13058-016-0741-1 (PMC4974783; doi:10.1186/s13058-016-0741-1)
Supplement: Additional file 1: — Report of peptides identified by tandem MS. (PDF 177 kb) [file 13058_2016_741_MOESM1_ESM.pdf]

Supplemental table 1. Proteins identified based on the peptides sequenced using tandem mass spectrometry. The BSA identified was from a quality control sample (25 fmol/ul) to test trypsin digestion and LC-MS/MS system performance.

Peak List Generator: unknown
Version: unknown
Charge States Calculated: unknown
Deisotoped: unknown
Textual Annotation: unknown
Database Set: 2 Databases
Database Name: the IPI bovin 20120411 database
Version: unknown
Taxonomy: All Entries
Number of Proteins: 30403
Database Name: the IPI human 20120411 database
Version: unknown
Taxonomy: All Entries
Number of Proteins: 91464
Does database contain common contaminants?: unknown
Search Engine Set: 1 Search Engine
Search Engine: Mascot
Version: 2.2.07
Samples: All Samples
Fragment Tolerance: 0.50 Da (Monoisotopic)
Parent Tolerance: 0.50 Da (Monoisotopic)
Fixed Modifications: +57 on C (Carbamidomethyl)
Variable Modifications: -17 on n (Pyro-cmC), +1 on NQ (Deamidation), +16 on M (Oxidation)
Database: the IPI bovin 20120411 database (unknown version, 30403 entries) (bsa (F098083)), the IPI human 20120411 database (unknown version, 91464 entries) (law1 (F098062), law2 (F098066), law3 (F098070), law4 (F098074), law5 (F098076), law6 (F098080))
Digestion Enzyme: Trypsin (bsa (F098083)), Trypsin (law1 (F098062), law2 (F098066), law3 (F098070), law4 (F098074), law5 (F098076), law6 (F098080))
Max Missed Cleavages: 1 (bsa (F098083)), 1 (law1 (F098062), law2 (F098066), law3 (F098070), law4 (F098074), law5 (F098076), law6 (F098080))
Scaffold Version: Scaffold 3.6.5
Peptide Thresholds: 90.0% minimum
Protein Thresholds: 99.0% minimum and 2 peptides minimum

Experimen	Biological s	Biological s	MS/MS sar	Protein name	Protein acc	Database s	Protein molecular w	Protein ide	Number of	Number of	Number of	Percentage	Percentage	Peptide sequence	Previous a	Next amin	Best Peptic	Best Masc	Best Masc	Best Masc	Number of	Number of	Number of	Number of	Number of	Calculated	Peptide sta	Peptide sto	Assigned	Other Proteins
20120614l	law	law1	law1 (F098	Keratin, type I cytoskeletal 10	IPI000098	IPI human	58,828.80	50.00%	1	1	1	0.60%	2.05%	ALEESNYELEGK	R	I	95.00%	64.3	38	52.6	0	1	0	0	2	1,381.65	166	177	TRUE	
20120614l	law	law1	law1 (F098	Isoform 1 of CUB domain-containing protein 1	IPI002900	IPI human	92,932.90	100.00%	6	6	6	3.59%	7.06%	AFMIIQEQR	R	T	95.00%	52.6	38	25.7	0	1	0	0	2	1,151.59	604	612	TRUE	
20120614l	law	law1	law1 (F098	Isoform 1 of CUB domain-containing protein 1	IPI002900	IPI human	92,932.90	100.00%	6	6	6	3.59%	7.06%	EEGVFTVTPDTK	K	S	95.00%	66	38.1	54.4	0	1	0	0	2	1,322.65	543	554	TRUE	
20120614l	law	law1	law1 (F098	Isoform 1 of CUB domain-containing protein 1	IPI002900	IPI human	92,932.90	100.00%	6	6	6	3.59%	7.06%	ISFLCDDLTR	K	L	95.00%	51.2	38	33.1	0	1	0	0	2	1,239.60	397	406	TRUE	
20120614l	law	law1	law1 (F098	Isoform 1 of CUB domain-containing protein 1	IPI002900	IPI human	92,932.90	100.00%	6	6	6	3.59%	7.06%	IYVVDLSNER	K	A	95.00%	60.3	38.4	42.4	0	1	0	0	2	1,207.63	344	353	TRUE	
20120614l	law	law1	law1 (F098	Isoform 1 of CUB domain-containing protein 1	IPI002900	IPI human	92,932.90	100.00%	6	6	6	3.59%	7.06%	LATEEPPPR	K	S	95.00%	59.8	38.1	34.2	0	1	0	0	2	1,009.53	788	796	TRUE	
20120614l	law	law1	law1 (F098	Isoform 1 of CUB domain-containing protein 1	IPI002900	IPI human	92,932.90	100.00%	6	6	6	3.59%	7.06%	SGVVCQTGR	R	A	95.00%	57.4	38.7	41.2	0	1	0	0	2	963.4677	595	603	TRUE	
20120614l	law	law1	law1 (F098	Isoform 1 of Plectin	IPI000148	IPI human	531,783.90	100.00%	2	2	2	1.20%	0.56%	LLDAQLSTGGIVDPSK	R	S	95.00%	99.8	38.1	74.1	0	1	0	0	2	1,613.87	3270	3285	TRUE	
20120614l	law	law1	law1 (F098	Isoform 1 of Plectin	IPI000148	IPI human	531,783.90	100.00%	2	2	2	1.20%	0.56%	LSVAAQEAAR	R	L	95.00%	50.8	38.5	29.7	0	1	0	0	2	1,015.55	2420	2429	TRUE	
20120614l	law	law1	law1 (F098	Isoform 1 of DNA-dependent protein kinase catalytic subunit	IPI002963	IPI human	469,095.50	100.00%	14	14	14	8.38%	3.75%	AQEPESGLSEETQVK	R	C	95.00%	71.8	38.1	54	0	1	0	0	2	1,631.78	4091	4105	TRUE	
20120614l	law	law1	law1 (F098	Isoform 1 of DNA-dependent protein kinase catalytic subunit	IPI002963	IPI human	469,095.50	100.00%	14	14	14	8.38%	3.75%	DQNILLGTTYR	R	I	95.00%	81.9	38.3	37.7	0	1	0	0	2	1,293.68	3325	3335	TRUE	
20120614l	law	law1	law1 (F098	Isoform 1 of DNA-dependent protein kinase catalytic subunit	IPI002963	IPI human	469,095.50	100.00%	14	14	14	8.38%	3.75%	LLALNSLYSPK	R	I	95.00%	50.7	38.6	43.8	0	1	0	0	2	1,218.71	2539	2549	TRUE	
20120614l	law	law1	law1 (F098	Isoform 1 of DNA-dependent protein kinase catalytic subunit	IPI002963	IPI human	469,095.50	100.00%	14	14	14	8.38%	3.75%	LLEEALLR	R	L	95.00%	53.8	37.9	19.9	0	1	0	0	2	956.5776	2892	2899	TRUE	
20120614l	law	law1	law1 (F098	Isoform 1 of DNA-dependent protein kinase catalytic subunit	IPI002963	IPI human	469,095.50	100.00%	14	14	14	8.38%	3.75%	LNESTFDTQITK	K	K	95.00%	90.3	38	69.5	0	1	0	0	2	1,396.70	1858	1869	TRUE	
20120614l	law	law1	law1 (F098	Isoform 1 of DNA-dependent protein kinase catalytic subunit	IPI002963	IPI human	469,095.50	100.00%	14	14	14	8.38%	3.75%	LPKDDVHAK	R	E	94.30%	38.2	38	14.9	0	1	0	0	2	1,022.56	1884	1892	TRUE	
20120614l	law	law1	law1 (F098	Isoform 1 of DNA-dependent protein kinase catalytic subunit	IPI002963	IPI human	469,095.50	100.00%	14	14	14	8.38%	3.75%	LQETLSAADR	R	C	95.00%	42.7	38.9	26.8	0	1	0	0	2	1,103.57	15	24	TRUE	
20120614l	law	law1	law1 (F098	Isoform 1 of DNA-dependent protein kinase catalytic subunit	IPI002963	IPI human	469,095.50	100.00%	14	14	14	8.38%	3.75%	NELEIPGQYDGR	R	G	95.00%	52.5	38.4	16.6	0	1	0	0	2	1,390.66	3697	3708	TRUE	
20120614l	law	law1	law1 (F098	Isoform 1 of DNA-dependent protein kinase catalytic subunit	IPI002963	IPI human	469,095.50	100.00%	14	14	14	8.38%	3.75%	QITQSALLAEAR	K	S	95.00%	48.7	37.9	35.6	0	1	0	0	2	1,283.70	2951	2962	TRUE	
20120614l	law	law1	law1 (F098	Isoform 1 of DNA-dependent protein kinase catalytic subunit	IPI002963	IPI human	469,095.50	100.00%	14	14	14	8.38%	3.75%	SLGPPQGEEDSVPR	R	D	95.00%	97.5	37.9	81.9	0	1	0	0	2	1,467.71	2107	2120	TRUE	
20120614l	law	law1	law1 (F098	Isoform 1 of DNA-dependent protein kinase catalytic subunit	IPI002963	IPI human	469,095.50	100.00%	14	14	14	8.38%	3.75%	SQGCSEQVLTVLK	R	T	95.00%	90.6	38.3	81.6	0	1	0	0	2	1,448.74	3290	3302	TRUE	
20120614l	law	law1	law1 (F098	Isoform 1 of DNA-dependent protein kinase catalytic subunit	IPI002963	IPI human	469,095.50	100.00%	14	14	14	8.38%	3.75%	TETVTSFR	R	K	91.10%	34.8	37.5	14.1	0	1	0	0	2	940.4734	3865	3872	TRUE	
20120614l	law	law1	law1 (F098	Isoform 1 of DNA-dependent protein kinase catalytic subunit	IPI002963	IPI human	469,095.50	100.00%	14	14	14	8.38%	3.75%	TSALSDETK	K	N	95.00%	57.5	38.3	41.9	0	1	0	0	2	951.463	802	810	TRUE	
20120614l	law	law1	law1 (F098	Isoform 1 of DNA-dependent protein kinase catalytic subunit	IPI002963	IPI human	469,095.50	100.00%	14	14	14	8.38%	3.75%	YPEETLSLMTK	R	E	95.00%	68.8	38.1	59	0	1	0	0	2	1,311.65	3475	3485	TRUE	
20120614l	law	law1	law1 (F098	Cytoplasmic dynein 1 heavy chain 1	IPI004569	IPI human	532,405.60	100.00%	10	10	10	5.99%	2.56%	ADLAAVEAK	K	V	95.00%	53.7	39.1	29.2	0	1	0	0	2	887.4833	3463	3471	TRUE	
20120614l	law	law1	law1 (F098	Cytoplasmic dynein 1 heavy chain 1	IPI004569	IPI human	532,405.60	100.00%	10	10	10	5.99%	2.56%	GNEIVLSAGSTPR	R	I	95.00%	66.3	38.2	59.8	0	1	0	0	2	1,300.69	3911	3923	TRUE	
20120614l	law	law1	law1 (F098	Cytoplasmic dynein 1 heavy chain 1	IPI004569	IPI human	532,405.60	100.00%	10	10	10	5.99%	2.56%	LGGSPFGPAGTGK	R	T	95.00%	65.6	38.7	52.5	0	1	0	0	2	1,145.60	1900	1912	TRUE	
20120614l	law	law1	law1 (F098	Cytoplasmic dynein 1 heavy chain 1	IPI004569	IPI human	532,405.60	100.00%	10	10	10	5.99%	2.56%	QLQNISLAAASGGAK	K	E	95.00%	48.8	38.2	35.7	0	1	0	0	2	1,428.78	4488	4502	TRUE	
20120614l	law	law1	law1 (F098	Cytoplasmic dynein 1 heavy chain 1	IPI004569	IPI human	532,405.60	100.00%	10	10	10	5.99%	2.56%	SSLQSQCLNEVLK	R	A	95.00%	46.1	38.2	7.98	0	1	0	0	2	1,505.76	3706	3718	TRUE	
20120614l	law	law1	law1 (F098	Cytoplasmic dynein 1 heavy chain 1	IPI004569	IPI human	532,405.60	100.00%	10	10	10	5.99%	2.56%	TAEVLANK	R	I	95.00%	43.7	39.1	30.8	0	1	0	0	2	845.4727	2061	2068	TRUE	
20120614l	law	law1	law1 (F098	Cytoplasmic dynein 1 heavy chain 1	IPI004569	IPI human	532,405.60	100.00%	10	10	10	5.99%	2.56%	VAAPDVVVPTLDTVR	K	H	95.00%	43	38.1	35	0	1	0	0	2	1,551.87	2562	2576	TRUE	
20120614l	law	law1	law1 (F098	Cytoplasmic dynein 1 heavy chain 1	IPI004569	IPI human	532,405.60	100.00%	10	10	10	5.99%	2.56%	VDNEFDQR	R	L	95.00%	42.5	38	24.9	0	1	0	0	2	1,022.45	4256	4263	TRUE	
20120614l	law	law1	law1 (F098	Cytoplasmic dynein 1 heavy chain 1	IPI004569	IPI human	532,405.60	100.00%	10	10	10	5.99%	2.56%	VLLTTQGVDMISK	R	M	90.50%	35	38.2	24.7	0	1	0	0	2	1,420.77	4330	4342	TRUE	
20120614l	law	law1	law1 (F098	Cytoplasmic dynein 1 heavy chain 1	IPI004569	IPI human	532,405.60	100.00%	10	10	10	5.99%	2.56%	WTDENIDTVALK	R	H	95.00%	43	38.2	27.5	0	1	0	0	2	1,404.70	2845	2856	TRUE	
20120614l	law	law2	law2 (F098	60 kDa heat shock protein, mitochondrial	IPI007841	IPI human	61,055.70	99.90%	2	2	2	1.27%	3.66%	TVIIEQSWGSPK	R	V	95.00%	38.7	38.1	25.7	0	1	0	0	2	1,344.72	61	72	TRUE	
20120614l	law	law2	law2 (F098	60 kDa heat shock protein, mitochondrial	IPI007841	IPI human	61,055.70	99.90%	2	2	2	1.27%	3.66%	VTDALNATR	R	A	95.00%	58.1	39	37.6	0	1	0	0	2	960.5109	421	429	TRUE	
20120614l	law	law2	law2 (F098	Calreticulin	IPI000205	IPI human	48,142.90	100.00%	4	4	4	2.53%	10.60%	EQFLDGDGWTSR	K	W	95.00%	68.7	37.9	51	0	1	0	0	2	1,410.63	25	36	TRUE	
20120614l	law	law2	law2 (F098	Calreticulin	IPI000205	IPI human	48,142.90	100.00%	4	4	4	2.53%	10.60%	FYGDEEKDK	K	G	93.50%	34.8	38.4	24.5	0	1	0	0	2	1,130.50	56	64	TRUE	
20120614l	law	law2	law2 (F098	Calreticulin	IPI000205	IPI human	48,142.90	100.00%	4	4	4	2.53%	10.60%	IKDPDASKPEDWDER	K	A	95.00%	55.5	37.7	44.8	0	0	1	0	2	1,800.84	208	222	TRUE	
20120614l	law	law2	law2 (F098	Calreticulin	IPI000205	IPI human	48,142.90	100.00%	4	4	4	2.53%	10.60%	QIDNPDYK	R	G	95.00%	41.6	38.4	11.3	0	1	0	0	2	992.4685	279	286	TRUE	
20120614l	law	law2	law2 (F098	Protein disulfide-isomerase	IPI000107	IPI human	57,118.10	99.50%	2	2	2	1.27%	3.74%	EADDIVNWLK	R	K	90.90%	33.2	38.7	17.9	0	1	0	0	2	1,202.61	121	130	TRUE	
20120614l	law	law2	law2 (F098	Protein disulfide-isomerase	IPI000107	IPI human	57,118.10	99.50%	2	2	2	1.27%	3.74%	LKAEGSEIR	K	L	95.00%	41.7	39	16.8	0	1	0	0	2	1,002.56	70	78	TRUE	
20120614l	law	law2	law2 (F098	Matrix metalloproteinase-14	IPI002183	IPI human	65,895.80	99.50%	2	2	2	1.27%	2.92%	AVDSEYPK	R	N	95.00%	39.9	38.3	24.2	0	1	0	0	2	908.436	444	451	TRUE	
20120614l	law	law2	law2 (F098	Matrix metalloproteinase-14	IPI002183	IPI human	65,895.80	99.50%	2	2	2	1.27%	2.92%	VWESATPLR	R	F	90.30%	33.1	38.9	16.5	0	1	0	0	2	1,058.56	150	158	TRUE	
20120614l	law	law2	law2 (F098	Isoform A of Prelamin-A/C	IPI000214	IPI human	74,140.70	100.00%	4	4	4	2.53%	6.48%	ITESEEVVSR	R	E	95.00%	54	38.3	41.5	0	1	0	0	2	1,148.58	63	72	TRUE	
20120614l	law	law2	law2 (F098	Isoform A of Prelamin-A/C	IPI000214	IPI human	74,140.70	100.00%	4	4	4	2.53%	6.48%	LADALQELR	R	A	95.00%	38	38.1	19.4	0	1	0	0	2	1,028.57	241	249	TRUE	
20120614l	law	law2	law2 (F098	Isoform A of Prelamin-A/C	IPI000214	IPI human	74,140.70	100.00%	4	4	4	2.53%	6.48%	SGAQASSTPLSPTR	R	I	95.00%	62.6	38.5	49	0	1	0	0	2	1,359.69	12	25	TRUE	
20120614l	law	law2	law2 (F098	Isoform A of Prelamin-A/C	IPI000214	IPI human	74,140.70	100.00%	4	4	4	2.53%	6.48%	SLETENAGLR	R	L	95.00%	47.8	38.8	26.1	0	1	0	0	2	1,089.55	51	60	TRUE	
20120614l	law	law2	law2 (F098	Isoform 1 of Sequestosome-1	IPI001794	IPI human	47,687.40	100.00%	4	4	4	2.53%	17.00%	AGEARPGPTAESASGPSEDPSVNFLK	R	N	95.00%	63	36.9	51.4	0	0	1	0	2	2,571.23	213	238	TRUE	
20120614l	law	law2	law2 (F098	Isoform 1 of Sequestosome-1	IPI001794	IPI human	47,687.40	100.00%	4	4	4	2.53%	17.00%	AYLLGKEDAAR	K	E	95.00%	64.9	38.6	46.5	0	1	0	0	2	1,206.65	8	18	TRUE	
20120614l	law	law2	law2 (F098	Isoform 1 of Sequestosome-1	IPI001794	IPI human	47,687.40	100.00%	4	4	4	2.53%	17.00%	FSFCCSPEPEAEAEAAAGPGPCER	R	L	93.00%	46.9	36.6	32.6	0	0	1	0	2	2,626.06	23	46	TRUE	
20120614l	law	law2	law2 (F098	Isoform 1 of Sequestosome-1	IPI001794	IPI human	47,687.40	100.00%	4	4	4	2.53%	17.00%	LTPVSPESSSTEEK	R	S	95.00%	53.2	38.1	36.7	0	1	0	0	2	1,490.72	268	281	TRUE	
20120614l	law	law2	law2 (F098	Isoform 1 of CUB domain-containing protein 1	IPI002900	IPI human	92,932.90	100.00%	11	13	13	8.23%	12.90%	AFMIIQEQR	R	T	95.00%	61	38	25.5	0	2	0	0	2	1,151.59	604	612	TRUE	
20120614l	law	law2	law2 (F098	Isoform 1 of CUB domain-containing protein 1	IPI002900	IPI human	92,932.90	100.00%	11	13	13	8.23%	12.90%	DQVACLTFFK	R	E	95.00%	64.6	38.3	40.5	0	1	0	0	2	1,228.60	583	592	TRUE	
20120614l	law	law2	law2 (F098	Isoform 1 of CUB domain-containing protein 1	IPI002900	IPI human	92,932.90	100.00%	11	13	13	8.23%	12.90%	EEGVFTVTPDTK	K	S	95.00%	63.6	38.1	52.6	0	1	0	0	2	1,322.65	543	554	TRUE	
20120614l	law	law2	law2 (F098	Isoform 1 of CUB domain-containing protein 1	IPI002900	IPI human	92,932.90	100.00%	11	13	13	8.23%	12.90%	GAEAFEIALPR	R	E	95.00%	91.9	38.8	64.1	0	1	0	0	2	1,173.63	26	36	TRUE	
20120614l	law	law2	law2 (F098	Isoform 1 of CUB domain-containing protein 1	IPI002900	IPI human	92,932.90	100.00%	11	13	13	8.23%	12.90%	ISFLCDDLTR	K	L	95.00%	50.1	38	33	0	1	0	0	2	1,239.60	397	406	TRUE	
20120614l	law	law2	law2 (F098	Isoform 1 of CUB domain-containing protein 1	IPI002900	IPI human	92,932.90	100.00%	11	13	13	8.23%	12.90%	LATEEPPPR	K	S	95.00%	44.5	38.1	22.2	0	1	0	0	2	1,009.53	788	796	TRUE	
20120614l	law	law2	law2 (F098	Isoform 1 of CUB domain-containing protein 1	IPI002900	IPI human	92,932.90	100.00%	11	13	13	8.23%	12.90%	LSLVLVPAQK	R	L	95.00%	67.3	37.9	33.4	0	1	0	0	2	1,067.68	457	466	TRUE	
20120614l	law	law2	law2 (F098	Isoform 1 of CUB domain-containing protein 1	IPI002900	IPI human	92,932.90	100.00%	11	13	13	8.23%	12.90%	LWMNVEK	R	T	94.50%	35.8	38.6	13.8	0	2	0	0	2	935.4656	407	413	TRUE	
20120614l	law	law2	law2 (F098	Isoform 1 of CUB domain-containing protein 1	IPI002900	IPI human	92,932.90	100.00%	11	13	13	8.23%	12.90%	QNISVTLR	K	T	95.00%	46.7	38.6	19.1	0	1	0	0	2	930.5367	511	518	TRUE	
20120614l	law	law2	law2 (F098	Isoform 1 of CUB domain-containing protein 1	IPI002900	IPI human	92,932.90	100.00%	11	13	13	8.23%	12.90%	SGVVCQTGR	R	A	95.00%	52.7	38.7	38.5	0	1	0	0	2	963.4677	595	603	TRUE	
20120614l	law	law2	law2 (F098	Isoform 1 of CUB domain-containing protein 1	IPI002900	IPI human	92,932.90	100.00%	11	13	13	8.23%	12.90%	TCSSNLTLTSGSK	R	H	95.00%	82.8	38.2	69.1	0	1	0	0	2	1,355.65	382	394	TRUE	
20120614l	law	law2	law2 (F098	Keratin, type II cytoskeletal 1	IPI002203	IPI human	66,040.30	99.50%	2	2	2	1.27%	3.26%	FLEQQNQVLQTK	R	W	90.60%	32.7	38.3	16.7	0	1	0	0	2	1,475.79	200	211	TRUE	
20120614l	law	law2	law2 (F098	Keratin, type II cytoskeletal 1	IPI002203	IPI human	66,040.30	99.50%	2	2	2	1.27%	3.26%	TLLEGEESR	R	M	95.00%	38.3	38.8	15.4	0	1	0	0	2	1,033.52	484	492	TRUE	
20120614l	law	law3	law3 (F098	ATP synthase subunit alpha, mitochondrial	IPI004404	IPI human	59,752.10	99.80%	2	2	2	0.62%	5.79%	AVDSLVPIGR	K	G	95.00%	39	37.7	22.5	0	1	0	0	2	1,026.59	195	204	TRUE	
20120614l	law	law3	law3 (F098	ATP synthase subunit alpha, mitochondrial	IPI004404	IPI human	59,752.10	99.80%	2	2	2	0.62%	5.79%	EVAAFAQFGSDLDAATQQLLSR	R	G	95.00%	43.5	37.1	36	0	0	1	0	2	2,338.17	442	463	TRUE	
20120614l	law	law3	law3 (F098	Keratin, type II cytoskeletal 7	IPI003069	IPI human	51,387.30	100.00%	4	4	4	1.24%	10.70%	FASFIDK	K	V	95.00%	43.1	37.2	0	0	1	0	0	2	827.4298	102	108	FALSE	IPI00554648,IPI01021414
20120614l	law	law3	law3 (F098	Keratin, type II cytoskeletal 7	IPI003069	IPI human	51,387.30	100.00%	4	4	4	1.24%	10.70%	FASFIDKVR	K	F	95.00%	50	38	0	0	1	0	0	2	1,082.60	102	110	FALSE	IPI00554648,IPI01021414
20120614l	law	law3	law3 (F098	Keratin, type II cytoskeletal 7	IPI003069	IPI human	51,387.30	100.00%	4	4	4	1.24%	10.70%	FETLQAQAGK	K	H	95.00%	40.2	38.5	27.3	0	1	0	0	2	1,092.57	287	296	TRUE	
20120614l	law	law3	law3 (F098	Keratin, type II cytoskeletal 7	IPI003069	IPI human	51,387.30	100.00%	4	4	4	1.24%	10.70%	LDADPSLQR	R	V	94.00%	35.3	38.2	17.1	0	1	0	0	2	1,014.52	78	86	TRUE	
20120614l	law	law3	law3 (F098	Keratin, type II cytoskeletal 7	IPI003069	IPI human	51,387.30	100.00%	4	4	4	1.24%	10.70%	SLDLDGIIAEVK	R	A	95.00%	75.7	38.3	52.2	0	1	0	0	2	1,272.70	254	265	TRUE	
20120614l	law	law3	law3 (F098	Keratin, type II cytoskeletal 7	IPI003069	IPI human	51,387.30	100.00%	4	4	4	1.24%	10.70%	VRQEESEQIK	R	T	95.00%	43.1	38.6	14.8	0	1	0	0	2	1,245.64	87	96	TRUE	
20120614l	law	law3	law3 (F098	Isoform 1 of E3 ubiquitin-protein ligase TRIM21	IPI000189	IPI human	54,169.50	100.00%	8	8	8	2.48%	20.20%	EAWDLGVCR	K	D	93.00%	35	38.7	23.7	0	1	0	0	2	1,105.51	352	360	TRUE	
20120614l	law	law3	law3 (F098	Isoform 1 of E3 ubiquitin-protein ligase TRIM21	IPI000189	IPI human	54,169.50	100.00%	8	8	8	2.48%	20.20%	HYWEVDVTGK	K	E	95.00%	54.4	38.6	41.2	0	1	0	0	2	1,233.59	342	351	TRUE	
20120614l	law	law3	law3 (F098	Isoform 1 of E3 ubiquitin-protein ligase TRIM21	IPI000189	IPI human	54,169.50	100.00%	8	8	8	2.48%	20.20%	LAQQSQALQELISELDRR	K	C	95.00%	70.6	37.6	64.5	0	0	1	0	2	2,098.13	218	235	TRUE	
20120614l	law	law3	law3 (F098	Isoform 1 of E3 ubiquitin-protein ligase TRIM21	IPI000189	IPI human	54,169.50	100.00%	8	8	8	2.48%	20.20%	LGDTQQSIPGNEER	R	F	95.00%	78.3	38.2	59.7	0	1	0	0	2	1,543.73	311	324	TRUE	
20120614l	law	law3	law3 (F098	Isoform 1 of E3 ubiquitin-protein ligase TRIM21	IPI000189	IPI human	54,169.50	100.00%	8	8	8	2.48%	20.20%	LQVALGELR	K	R	95.00%	58.4	37.6	18.5	0	1	0	0	2	998.5994	139	147	TRUE	
20120614l	law	law3	law3 (F098	Isoform 1 of E3 ubiquitin-protein ligase TRIM21	IPI000189	IPI human	54,169.50	100.00%	8	8	8	2.48%	20.20%	NFLVEEEQR	K	Q	95.00%	50.8	38.4	25.3	0	1	0	0	2	1,163.57	187	195	TRUE	
20120614l	law	law3	law3 (F098	Isoform 1 of E3 ubiquitin-protein ligase TRIM21	IPI000189	IPI human	54,169.50	100.00%	8	8	8	2.48%	20.20%	QLQELEKDER	R	E	95.00%	45.7	38.5	9.06	0	1	0	0	2	1,287.65	196	205	TRUE	
20120614l	law	law3	law3 (F098	Isoform 1 of E3 ubiquitin-protein ligase TRIM21	IPI000189	IPI human	54,169.50	100.00%	8	8	8	2.48%	20.20%	SESWNLKDLDITSPELR	R	S	95.00%	37.3	37.6	26	0	0	1	0	2	2,003.01	254	270	TRUE	
20120614l	law	law3	law3 (F098	Tubulin alpha-1A chain	IPI001806	IPI human	50,135.70	99.80%	2	2	2	0.62%	46.80%	AVCMLSNTTAIAEAWAR	R	L	95.00%	149	37.7	129	0	2	0	0	2	1,864.90	374	390	FALSE	IPI00930688
20120614l	law	law3	law3 (F098	Tubulin alpha-1A chain	IPI001806	IPI human	50,135.70	99.80%	2	2	2	0.62%	46.80%	AVFVDLEPTVIDEVR	R	T	95.00%	106	37.8	91.9	0	4	0	0	2	1,701.91	65	79	FALSE	IPI00930688
20120614l	law	law3	law3 (F098	Tubulin alpha-1A chain	IPI001806	IPI human	50,135.70	99.80%	2	2	2	0.62%	46.80%	AYHEQLSVAEITNACFEPANQMVK	K	C	95.00%	88.2	36.4	78.1	0	0	2	0	2	2,750.29	281	304	FALSE	IPI00930688
20120614l	law	law3	law3 (F098	Tubulin alpha-1A chain	IPI001806	IPI human	50,135.70	99.80%	2	2	2	0.62%	46.80%	DVNAAIATIK	K	T	95.00%	46.5	38.5	24.5	0	1	0	0	2	1,015.58	327	336	FALSE	IPI00930688
20120614l	law	law3	law3 (F098	Tubulin alpha-1A chain	IPI001806	IPI human	50,135.70	99.80%	2	2	2	0.62%	46.80%	EDAANNYAR	K	G	95.00%	71.8	38.1	52.2	0	1	0	0	2	1,023.45	97	105	FALSE	IPI00930688
20120614l	law	law3	law3 (F098	Tubulin alpha-1A chain	IPI001806	IPI human	50,135.70	99.80%	2	2	2	0.62%	46.80%	EIIDLVLDR	K	I	95.00%	58.5	38.1	33.5	0	1	0	0	2	1,085.62	113	121	FALSE	IPI00930688

20120614l	law	law3	law3 (F098	Tubulin alpha-1A chain	IPI0018067	IPI	human	50,135.70	99.80%	2	2	2	0.62%	46.80%	FDLMYAK	K	R	94.30%	36.4	39.1	20.7	0	1	0	0	2	887.4332	395	401	FALSE	IPI00930688
20120614l	law	law3	law3 (F098	Tubulin alpha-1A chain	IPI0018067	IPI	human	50,135.70	99.80%	2	2	2	0.62%	46.80%	IHFPLATYAPVISAEK	R	A	95.00%	51.5	37.8	39.5	0	0	1	0	2	1,756.96	265	280	FALSE	IPI00930688
20120614l	law	law3	law3 (F098	Tubulin alpha-1A chain	IPI0018067	IPI	human	50,135.70	99.80%	2	2	2	0.62%	46.80%	LDHKFDLMYAK	R	R	95.00%	59.2	38.1	39.7	0	0	1	0	2	1,380.70	391	401	FALSE	IPI00930688
20120614l	law	law3	law3 (F098	Tubulin alpha-1A chain	IPI0018067	IPI	human	50,135.70	99.80%	2	2	2	0.62%	46.80%	LIGQIVSSITASLR	R	F	95.00%	93.2	38.1	82.1	0	1	0	0	2	1,457.87	230	243	TRUE	
20120614l	law	law3	law3 (F098	Tubulin alpha-1A chain	IPI0018067	IPI	human	50,135.70	99.80%	2	2	2	0.62%	46.80%	NLDIERPTYTNLNR	R	L	95.00%	47.3	38	28.7	0	1	0	0	2	1,718.88	216	229	FALSE	IPI00930688
20120614l	law	law3	law3 (F098	Tubulin alpha-1A chain	IPI0018067	IPI	human	50,135.70	99.80%	2	2	2	0.62%	46.80%	QLFHPEQLITGK	R	E	95.00%	46.5	37.9	0	0	1	0	0	2	1,410.77	85	96	FALSE	IPI00930688
20120614l	law	law3	law3 (F098	Tubulin alpha-1A chain	IPI0018067	IPI	human	50,135.70	99.80%	2	2	2	0.62%	46.80%	QLFHPEQLITGKEDAANNYAR	R	G	95.00%	72.8	37.4	53.4	0	0	2	0	2	2,415.21	85	105	FALSE	IPI00930688
20120614l	law	law3	law3 (F098	Tubulin alpha-1A chain	IPI0018067	IPI	human	50,135.70	99.80%	2	2	2	0.62%	46.80%	TIGGGDDSFNTFFSETGAGK	K	H	95.00%	154	37.4	143	0	1	0	0	2	2,007.89	41	60	FALSE	IPI00930688
20120614l	law	law3	law3 (F098	Tubulin alpha-1A chain	IPI0018067	IPI	human	50,135.70	99.80%	2	2	2	0.62%	46.80%	TIQFVDWCPTGFK	R	V	95.00%	52.7	38	37.8	0	1	0	0	2	1,598.77	340	352	TRUE	
20120614l	law	law3	law3 (F098	Tubulin alpha-1A chain	IPI0018067	IPI	human	50,135.70	99.80%	2	2	2	0.62%	46.80%	VGINYQPPTVVPGGDLAK	K	V	95.00%	73.4	37.7	54.6	0	1	0	0	2	1,824.99	353	370	FALSE	IPI00930688
20120614l	law	law3	law3 (F098	Tubulin alpha-1A chain	IPI0018067	IPI	human	50,135.70	99.80%	2	2	2	0.62%	46.80%	YMACCLLYR	K	G	95.00%	55.6	38.3	51.5	0	2	0	0	2	1,249.55	312	320	FALSE	IPI00930688
20120614l	law	law3	law3 (F098	Keratin, type II cytoskeletal 8	IPI0055464	IPI	human	53,706.20	100.00%	10	10	10	3.11%	20.30%	AQYEDIANR	K	S	95.00%	39.1	38.3	28.6	0	1	0	0	2	1,079.51	265	273	TRUE	
20120614l	law	law3	law3 (F098	Keratin, type II cytoskeletal 8	IPI0055464	IPI	human	53,706.20	100.00%	10	10	10	3.11%	20.30%	ASLEAAIADAEQR	R	G	95.00%	61.8	38.1	48.6	0	1	0	0	2	1,344.68	329	341	TRUE	
20120614l	law	law3	law3 (F098	Keratin, type II cytoskeletal 8	IPI0055464	IPI	human	53,706.20	100.00%	10	10	10	3.11%	20.30%	FASFIDK	K	V	95.00%	43.1	37.2	0	0	1	0	0	2	827.4298	102	108	TRUE	IPI00306959,IPI01021185,IPI00792841
20120614l	law	law3	law3 (F098	Keratin, type II cytoskeletal 8	IPI0055464	IPI	human	53,706.20	100.00%	10	10	10	3.11%	20.30%	FASFIDKVR	K	F	95.00%	50	38	0	0	1	0	0	2	1,082.60	102	110	TRUE	IPI00306959,IPI01021185,IPI00792841
20120614l	law	law3	law3 (F098	Keratin, type II cytoskeletal 8	IPI0055464	IPI	human	53,706.20	100.00%	10	10	10	3.11%	20.30%	LEGLTDEINFLR	R	Q	95.00%	85.4	38.3	55.5	0	1	0	0	2	1,419.75	214	225	TRUE	
20120614l	law	law3	law3 (F098	Keratin, type II cytoskeletal 8	IPI0055464	IPI	human	53,706.20	100.00%	10	10	10	3.11%	20.30%	LQAEIEGLKGQR	R	A	95.00%	46.2	38.2	32.4	0	1	0	0	2	1,341.75	317	328	TRUE	
20120614l	law	law3	law3 (F098	Keratin, type II cytoskeletal 8	IPI0055464	IPI	human	53,706.20	100.00%	10	10	10	3.11%	20.30%	LVSESSDVLPK	K	-	95.00%	52.3	38.8	37.5	0	1	0	0	2	1,173.64	473	483	TRUE	
20120614l	law	law3	law3 (F098	Keratin, type II cytoskeletal 8	IPI0055464	IPI	human	53,706.20	100.00%	10	10	10	3.11%	20.30%	SLDMDSIIAEVK	R	A	95.00%	73.1	38.4	63.5	0	1	0	0	2	1,320.67	253	264	TRUE	
20120614l	law	law3	law3 (F098	Keratin, type II cytoskeletal 8	IPI0055464	IPI	human	53,706.20	100.00%	10	10	10	3.11%	20.30%	TKTEISEMNR	R	N	95.00%	39.2	38	29.5	0	1	0	0	2	1,224.59	303	312	TRUE	
20120614l	law	law3	law3 (F098	Keratin, type II cytoskeletal 8	IPI0055464	IPI	human	53,706.20	100.00%	10	10	10	3.11%	20.30%	YEELQSLAGK	K	H	95.00%	36.6	38.3	12.5	0	1	0	0	2	1,137.58	286	295	TRUE	
20120614l	law	law3	law3 (F098	Fatty acyl-CoA reductase 1	IPI0047883	IPI	human	59,358.20	100.00%	3	3	3	0.93%	6.41%	ALAEYVVQQEGAK	K	L	95.00%	96.7	38.2	77.1	0	1	0	0	2	1,405.73	209	221	TRUE	
20120614l	law	law3	law3 (F098	Fatty acyl-CoA reductase 1	IPI0047883	IPI	human	59,358.20	100.00%	3	3	3	0.93%	6.41%	NVLLTGATGFLGK	K	V	95.00%	87.2	38.3	64	0	1	0	0	2	1,290.74	12	24	TRUE	
20120614l	law	law3	law3 (F098	Fatty acyl-CoA reductase 1	IPI0047883	IPI	human	59,358.20	100.00%	3	3	3	0.93%	6.41%	TFNIDVR	K	Q	94.90%	36.7	38.9	27.6	0	1	0	0	2	864.4574	420	426	TRUE	
20120614l	law	law3	law3 (F098	Isoform 1 of PDZ and LIM domain protein 7	IPI0002312	IPI	human	49,844.40	100.00%	4	4	4	1.24%	9.85%	ASAPAADPPR	K	Y	95.00%	38.3	37.7	25.5	0	1	0	0	2	952.4847	94	103	TRUE	
20120614l	law	law3	law3 (F098	Isoform 1 of PDZ and LIM domain protein 7	IPI0002312	IPI	human	49,844.40	100.00%	4	4	4	1.24%	9.85%	HSQPATPTPLQSR	R	T	95.00%	54.5	38.3	45.2	0	1	0	0	2	1,419.73	246	258	TRUE	
20120614l	law	law3	law3 (F098	Isoform 1 of PDZ and LIM domain protein 7	IPI0002312	IPI	human	49,844.40	100.00%	4	4	4	1.24%	9.85%	VLEEGGFFEEK	K	G	95.00%	44.7	37.9	25.7	0	1	0	0	2	1,283.62	314	324	TRUE	
20120614l	law	law3	law3 (F098	Isoform 1 of PDZ and LIM domain protein 7	IPI0002312	IPI	human	49,844.40	100.00%	4	4	4	1.24%	9.85%	YTFAPSVSLNK	R	T	95.00%	39.5	37.8	31.4	0	1	0	0	2	1,226.64	104	114	TRUE	
20120614l	law	law3	law3 (F098	Tubulin beta chain	IPI0101911	IPI	human	49,670.60	100.00%	23	32	33	10.20%	56.10%	AILVDLEPGTMDSVR	R	S	95.00%	69.7	38.1	56.7	0	1	0	0	2	1,631.83	63	77	TRUE	
20120614l	law	law3	law3 (F098	Tubulin beta chain	IPI0101911	IPI	human	49,670.60	100.00%	23	32	33	10.20%	56.10%	ALTVPELTQQVFDAK	R	N	95.00%	75.5	38	58.5	0	1	0	0	2	1,659.90	283	297	TRUE	
20120614l	law	law3	law3 (F098	Tubulin beta chain	IPI0101911	IPI	human	49,670.60	100.00%	23	32	33	10.20%	56.10%	EIVHIQAGQCGNQIGAK	R	F	95.00%	96.1	37.9	0	0	1	0	0	2	1,822.92	3	19	TRUE	IPI00007752
20120614l	law	law3	law3 (F098	Tubulin beta chain	IPI0101911	IPI	human	49,670.60	100.00%	23	32	33	10.20%	56.10%	EVDEQMLNVQNK	K	N	95.00%	90.2	38.3	66.7	0	2	0	0	2	1,462.68	325	336	TRUE	IPI00007752
20120614l	law	law3	law3 (F098	Tubulin beta chain	IPI0101911	IPI	human	49,670.60	100.00%	23	32	33	10.20%	56.10%	FPGQLNADLR	R	K	95.00%	67.7	38.4	46.2	0	1	0	0	2	1,130.60	242	251	TRUE	IPI00007752
20120614l	law	law3	law3 (F098	Tubulin beta chain	IPI0101911	IPI	human	49,670.60	100.00%	23	32	33	10.20%	56.10%	FPGQLNADLRK	R	L	95.00%	48.8	38.5	33.7	0	1	0	0	2	1,258.69	242	252	TRUE	IPI00007752
20120614l	law	law3	law3 (F098	Tubulin beta chain	IPI0101911	IPI	human	49,670.60	100.00%	23	32	33	10.20%	56.10%	GHYTEGAELVDSVLDVVR	K	K	95.00%	76.6	37.5	65.5	0	0	1	0	2	1,958.98	104	121	TRUE	IPI00007752
20120614l	law	law3	law3 (F098	Tubulin beta chain	IPI0101911	IPI	human	49,670.60	100.00%	23	32	33	10.20%	56.10%	GHYTEGAELVDSVLDVVRK	K	E	95.00%	64.8	37.6	51.4	0	0	1	0	2	2,087.08	104	122	TRUE	IPI00007752
20120614l	law	law3	law3 (F098	Tubulin beta chain	IPI0101911	IPI	human	49,670.60	100.00%	23	32	33	10.20%	56.10%	IMNTFSVVPSPK	R	V	95.00%	85.4	38.4	73.2	0	2	0	0	2	1,319.70	163	174	TRUE	IPI00007752
20120614l	law	law3	law3 (F098	Tubulin beta chain	IPI0101911	IPI	human	49,670.60	100.00%	23	32	33	10.20%	56.10%	ISEQFTAMFR	R	R	95.00%	55.4	38.6	37.5	0	2	0	0	2	1,245.59	381	390	TRUE	IPI00007752
20120614l	law	law3	law3 (F098	Tubulin beta chain	IPI0101911	IPI	human	49,670.60	100.00%	23	32	33	10.20%	56.10%	ISVYYNEATGGK	R	Y	95.00%	73	38.3	60.3	0	1	0	0	2	1,301.64	47	58	TRUE	
20120614l	law	law3	law3 (F098	Tubulin beta chain	IPI0101911	IPI	human	49,670.60	100.00%	23	32	33	10.20%	56.10%	KLAVNMVPFPR	R	L	95.00%	50.8	38.3	39.3	0	1	0	0	2	1,271.73	252	262	TRUE	IPI00007752
20120614l	law	law3	law3 (F098	Tubulin beta chain	IPI0101911	IPI	human	49,670.60	100.00%	23	32	33	10.20%	56.10%	LAVNMVPFPR	K	L	95.00%	65.7	38.7	55.5	0	2	0	0	2	1,159.63	253	262	TRUE	IPI00007752
20120614l	law	law3	law3 (F098	Tubulin beta chain	IPI0101911	IPI	human	49,670.60	100.00%	23	32	33	10.20%	56.10%	LHFFMPGFAPLTSR	R	G	95.00%	57.5	37.8	45.1	0	2	0	0	2	1,636.83	263	276	TRUE	IPI00007752
20120614l	law	law3	law3 (F098	Tubulin beta chain	IPI0101911	IPI	human	49,670.60	100.00%	23	32	33	10.20%	56.10%	LTTPTYGDLNHLVSATMSGVTTCLR	K	F	95.00%	84.4	36.8	68	0	0	2	0	2	2,708.34	217	241	TRUE	IPI00007752
20120614l	law	law3	law3 (F098	Tubulin beta chain	IPI0101911	IPI	human	49,670.60	100.00%	23	32	33	10.20%	56.10%	MAVTFIGNSTAIQELFK	K	R	95.00%	143	37.7	107	0	2	0	0	2	1,885.97	363	379	TRUE	
20120614l	law	law3	law3 (F098	Tubulin beta chain	IPI0101911	IPI	human	49,670.60	100.00%	23	32	33	10.20%	56.10%	MAVTFIGNSTAIQELFKR	K	I	95.00%	72.9	37.7	59.7	0	0	1	0	2	2,042.07	363	380	TRUE	
20120614l	law	law3	law3 (F098	Tubulin beta chain	IPI0101911	IPI	human	49,670.60	100.00%	23	32	33	10.20%	56.10%	MREIVHIQAGQCGNQIGAK	-	F	95.00%	68.9	37.7	0	0	0	2	0	2	2,110.06	1	19	TRUE	IPI00007752
20120614l	law	law3	law3 (F098	Tubulin beta chain	IPI0101911	IPI	human	49,670.60	100.00%	23	32	33	10.20%	56.10%	MSMKEVDEQMLNVQNK	R	N	95.00%	37.7	37.5	30.8	0	0	1	0	2	1,971.88	321	336	TRUE	IPI00007752
20120614l	law	law3	law3 (F098	Tubulin beta chain	IPI0101911	IPI	human	49,670.60	100.00%	23	32	33	10.20%	56.10%	NMMAACDPR	K	H	95.00%	42.9	37.9	1.18	0	3	0	0	2	1,081.42	298	306	TRUE	IPI00007752
20120614l	law	law3	law3 (F098	Tubulin beta chain	IPI0101911	IPI	human	49,670.60	100.00%	23	32	33	10.20%	56.10%	SGPFGQIFRPDNFVFGQSGAGNNWAK	R	G	95.00%	103	36.7	81.8	0	0	1	0	2	2,798.34	78	103	TRUE	IPI00007752
20120614l	law	law3	law3 (F098	Tubulin beta chain	IPI0101911	IPI	human	49,670.60	100.00%	23	32	33	10.20%	56.10%	TAVCDIPPR	K	G	95.00%	66.1	38.1	33.5	0	1	0	0	2	1,028.52	351	359	TRUE	IPI00007752
20120614l	law	law3	law3 (F098	Tubulin beta chain	IPI0101911	IPI	human	49,670.60	100.00%	23	32	33	10.20%	56.10%	YLTVAAVFR	R	G	95.00%	61.6	38	42.1	0	1	0	0	2	1,039.59	310	318	TRUE	IPI00007752
20120614l	law	law3	law3 (F098	Tubulin alpha-1B chain	IPI0093068	IPI	human	50,151.70	100.00%	17	21	24	7.45%	46.80%	AVCMLSNTTAIAEAWAR	R	L	95.00%	149	37.7	129	0	2	0	0	2	1,864.90	374	390	TRUE	IPI00180675,IPI01022358
20120614l	law	law3	law3 (F098	Tubulin alpha-1B chain	IPI0093068	IPI	human	50,151.70	100.00%	17	21	24	7.45%	46.80%	AVFVDLEPTVIDEVR	R	T	95.00%	106	37.8	91.9	0	4	0	0	2	1,701.91	65	79	TRUE	IPI00180675,IPI01022358
20120614l	law	law3	law3 (F098	Tubulin alpha-1B chain	IPI0093068	IPI	human	50,151.70	100.00%	17	21	24	7.45%	46.80%	AYHEQLSVAEITNACFEPANQMVK	K	C	95.00%	88.2	36.4	78.1	0	0	2	0	2	2,750.29	281	304	TRUE	IPI00180675,IPI01022358
20120614l	law	law3	law3 (F098	Tubulin alpha-1B chain	IPI0093068	IPI	human	50,151.70	100.00%	17	21	24	7.45%	46.80%	DVNAAIATIK	K	T	95.00%	46.5	38.5	24.5	0	1	0	0	2	1,015.58	327	336	TRUE	IPI00180675,IPI01022358
20120614l	law	law3	law3 (F098	Tubulin alpha-1B chain	IPI0093068	IPI	human	50,151.70	100.00%	17	21	24	7.45%	46.80%	EDAANNYAR	K	G	95.00%	71.8	38.1	52.2	0	1	0	0	2	1,023.45	97	105	TRUE	IPI00180675,IPI01022358
20120614l	law	law3	law3 (F098	Tubulin alpha-1B chain	IPI0093068	IPI	human	50,151.70	100.00%	17	21	24	7.45%	46.80%	EIIDLVLDR	K	I	95.00%	58.5	38.1	33.5	0	1	0	0	2	1,085.62	113	121	TRUE	IPI00180675,IPI01022358
20120614l	law	law3	law3 (F098	Tubulin alpha-1B chain	IPI0093068	IPI	human	50,151.70	100.00%	17	21	24	7.45%	46.80%	FDLMYAK	K	R	94.30%	36.4	39.1	20.7	0	1	0	0	2	887.4332	395	401	TRUE	IPI00180675,IPI01022358
20120614l	law	law3	law3 (F098	Tubulin alpha-1B chain	IPI0093068	IPI	human	50,151.70	100.00%	17	21	24	7.45%	46.80%	IHFPLATYAPVISAEK	R	A	95.00%	51.5	37.8	39.5	0	0	1	0	2	1,756.96	265	280	TRUE	IPI00180675,IPI01022358
20120614l	law	law3	law3 (F098	Tubulin alpha-1B chain	IPI0093068	IPI	human	50,151.70	100.00%	17	21	24	7.45%	46.80%	LDHKFDLMYAK	R	R	95.00%	59.2	38.1	39.7	0	0	1	0	2	1,380.70	391	401	TRUE	IPI00180675,IPI01022358
20120614l	law	law3	law3 (F098	Tubulin alpha-1B chain	IPI0093068	IPI	human	50,151.70	100.00%	17	21	24	7.45%	46.80%	LISQIVSSITASLR	R	F	95.00%	103	38.3	88.7	0	1	0	0	2	1,487.88	230	243	TRUE	
20120614l	law	law3	law3 (F098	Tubulin alpha-1B chain	IPI0093068	IPI	human	50,151.70	100.00%	17	21	24	7.45%	46.80%	NLDIERPTYTNLNR	R	L	95.00%	47.3	38	28.7	0	1	0	0	2	1,718.88	216	229	TRUE	IPI00180675,IPI01022358
20120614l	law	law3	law3 (F098	Tubulin alpha-1B chain	IPI0093068	IPI	human	50,151.70	100.00%	17	21	24	7.45%	46.80%	QLFHPEQLITGK	R	E	95.00%	46.5	37.9	0	0	1	0	0	2	1,410.77	85	96	TRUE	IPI00180675,IPI01022358
20120614l	law	law3	law3 (F098	Tubulin alpha-1B chain	IPI0093068	IPI	human	50,151.70	100.00%	17	21	24	7.45%	46.80%	QLFHPEQLITGKEDAANNYAR	R	G	95.00%	72.8	37.4	53.4	0	0	2	0	2	2,415.21	85	105	TRUE	IPI00180675,IPI01022358
20120614l	law	law3	law3 (F098	Tubulin alpha-1B chain	IPI0093068	IPI	human	50,151.70	100.00%	17	21	24	7.45%	46.80%	SIQFVDWCPTGFK	R	V	95.00%	67.6	38	48	0	1	0	0	2	1,584.75	340	352	TRUE	
20120614l	law	law3	law3 (F098	Tubulin alpha-1B chain	IPI0093068	IPI	human	50,151.70	100.00%	17	21	24	7.45%	46.80%	TIGGGDDSFNTFFSETGAGK	K	H	95.00%	154	37.4	143	0	1	0	0	2	2,007.89	41	60	TRUE	IPI00180675,IPI01022358
20120614l	law	law3	law3 (F098	Tubulin alpha-1B chain	IPI0093068	IPI	human	50,151.70	100.00%	17	21	24	7.45%	46.80%	VGINYQPPTVVPGGDLAK	K	V	95.00%	73.4	37.7	54.6	0	1	0	0	2	1,824.99	353	370	TRUE	IPI00180675,IPI01022358
20120614l	law	law3	law3 (F098	Tubulin alpha-1B chain	IPI0093068	IPI	human	50,151.70	100.00%	17	21	24	7.45%	46.80%	YMACCLLYR	K	G	95.00%	55.6	38.3	51.5	0	2	0	0	2	1,249.55	312	320	TRUE	IPI00180675,IPI01022358
20120614l	law	law3	law3 (F098	Tubulin beta-2C chain	IPI0000775	IPI	human	49,830.70	100.00%	4	6	6	1.86%	55.70%	ALTVPELTQQMFDAK	R	N	95.00%	67.1	37.8	32	0	1	0	0	2	1,707.86	283	297	TRUE	
20120614l	law	law3	law3 (F098	Tubulin beta-2C chain	IPI0000775	IPI	human	49,830.70	100.00%	4	6	6	1.86%	55.70%	AVLVDLEPGTMDSVR	R	S	95.00%	46.9	38	35.5	0	2	0	0	2	1,617.82	63	77	TRUE	
20120614l	law	law3	law3 (F098	Tubulin beta-2C chain	IPI0000775	IPI	human	49,830.70	100.00%	4	6	6	1.86%	55.70%	EIVHLQAGQCGNQIGAK	R	F	95.00%	96.1	37.9	0	0	1	0	0	2	1,822.92	3	19	FALSE	IPI01020686,IPI01022164,IPI01019113
20120614l	law	law3	law3 (F098	Tubulin beta-2C chain	IPI0000775	IPI	human	49,830.70	100.00%	4	6	6	1.86%	55.70%	EVDEQMLNVQNK	K	N	95.00%	90.2	38.3	66.7	0	2	0	0	2	1,462.68	325	336	FALSE	IPI01020686,IPI01022164,IPI01019113
20120614l	law	law3	law3 (F098	Tubulin beta-2C chain	IPI0000775	IPI	human	49,830.70	100.00%	4	6	6	1.86%	55.70%	FPGQLNADLR	R	K	95.00%	67.7	38.4	46.2	0	1	0	0	2	1,130.60	242	251	FALSE	IPI01020686,IPI01022164,IPI01019113
20120614l	law	law3	law3 (F098	Tubulin beta-2C chain	IPI0000775	IPI	human	49,830.70	100.00%	4	6	6	1.86%	55.70%	FPGQLNADLRK	R	L	95.00%	48.8	38.5	33.7	0	1	0	0	2	1,258.69	242	252	FALSE	IPI01020686,IPI01022164,IPI01019113
20120614l	law	law3	law3 (F098	Tubulin beta-2C chain	IPI0000775	IPI	human	49,830.70	100.00%	4	6	6	1.86%	55.70%	GHYTEGAELVDSVLDVVR	K	K	95.00%	76.6	37.5	65.5	0	0	1	0	2	1,958.98	104	121	FALSE	IPI01020686,IPI01022164,IPI01019113
20120614l	law	law3	law3 (F098	Tubulin beta-2C chain	IPI0000775	IPI	human	49,830.70	100.00%	4	6	6	1.86%	55.70%	GHYTEGAELVDSVLDVVRK	K	E	95.00%	64.8	37.6	51.4	0	0	1	0	2	2,087.08	104	122	FALSE	IPI01020686,IPI01022164,IPI01019113
20120614l	law	law3	law3 (F098	Tubulin beta-2C chain	IPI0000775	IPI	human	49,830.70	100.00%	4	6	6	1.86%	55.70%	IMNTFSVVPSPK	R	V	95.00%	85.4	38.4	73.2	0	2	0	0	2	1,319.70	163	174	FALSE	IPI01020686,IPI01022164,IPI01019113
20120614l	law	law3	law3 (F098	Tubulin beta-2C chain	IPI0000775	IPI	human	49,830.70	100.00%	4	6	6	1.86%	55.70%	INVYYNEATGGK	R	Y	95.00%	50.7	38.2	41.8	0	1	0	0	2	1,328.65	47	58	TRUE	
20120614l	law	law3	law3 (F098	Tubulin beta-2C chain	IPI0000775	IPI	human	49,830.70	100.00%	4	6	6	1.86%	55.70%	ISEQFTAMFR	R	R	95.00%	55.4	38.6	37.5	0	2	0	0	2	1,245.59	381	390	FALSE	IPI01020686,IPI01022164,IPI01019113
20120614l	law	law3	law3 (F098	Tubulin beta-2C chain	IPI0000775	IPI	human	49,830.70	100.00%	4	6	6	1.86%	55.70%	KLAVNMVPFPR	R	L	95.00%	50.8	38.3	39.3	0	1	0	0	2	1,271.73	252	262	FALSE	IPI01020686,IPI01022164,IPI01019113
20120614l	law	law3	law3 (F098	Tubulin beta-2C chain	IPI0000775	IPI	human	49,830.70	100.00%	4	6	6	1.86%	55.70%	LAVNMVPFPR	K	L	95.00%	65.7	38.7	55.5	0	2	0	0	2	1,159.63	253	262	FALSE	IPI01020686,IPI01022164,IPI01019113
20120614l	law	law3	law3 (F098	Tubulin beta-2C chain	IPI0000775	IPI	human	49,830.70	100.00%	4	6	6	1.86%	55.70%	LHFFMPGFAPLTSR	R	G	95.00%	57.5	37.8	45.1	0	2	0	0	2	1,636.83	263	276	FALSE	IPI01020686,IPI01022164,IPI01019113
20120614l	law	law3	law3 (F098	Tubulin beta-2C chain	IPI0000775	IPI	human	49,830.70	100.00%	4	6	6	1.86%	55.70%	LTTPTYGDLNHLVSATMSGVTTCLR	K	F	95.00%	84.4	36.8	68	0	0	2	0	2	2,708.34	217	241	FALSE	IPI01020686,IPI01022164,IPI01019113
20120614l	law	law3	law3 (F098	Tubulin beta-2C chain	IPI0000775	IPI	human	49,830.70	100.00%	4	6	6	1.86%	55.70%	MREIVHLQAGQCGNQIGAK	-	F	95.00%	68.9	37.7	0	0	0	2	0	2	2,110.06	1	19	FALSE	IPI01020686,IPI01022164,IPI01019113
20120614l	law	law3	law3 (F098	Tubulin beta-2C chain	IPI0000775	IPI	human	49,830.70	100.00%	4	6	6	1.86%	55.70%	MSATFIGNSTAIQELFK	K	R	95.00%	111	37.7	1.82	0	2	0	0	2	1,857.94	363	379	TRUE	
20120614l	law	law3	law3 (F098	Tubulin beta-2C chain	IPI0000775	IPI	human	49,830.70	100.00%	4	6	6	1.86%	55.70%	MSMKEVDEQMLNVQNK	R	N	95.00%	37.7	37.5	30.8	0	0	1	0	2	1,971.88	321	336	FALSE	IPI01020686,IPI01022164,IPI01019113
20120614l	law	law3	law3 (F098	Tubulin beta-2C chain	IPI0000775	IPI	human	49,830.70	100.00%	4	6	6	1.86%	55.70%	NMMAACDPR	K	H	95.00%	42.9	37.9	1.18	0	3	0	0	2	1,081.42	298	306	FALSE	IPI01020686,IPI01022164,IPI01019113
20120614l	law	law3	law3 (F098	Tubulin beta-2C chain	IPI0000775	IPI	human	49,830.70	100.00%	4	6	6	1.86%	55.70%	SGPFGQIFRPDNFVFGQSGAGNNWAK	R	G	95.00%	103	36.7	81.8	0	0	1	0	2	2,798.34	78	103	FALSE	IPI01020686,IPI01022164,IPI01019113
20120614l	law	law3	law3 (F098	Tubulin beta-2C chain	IPI0000775	IPI	human	49,830.70	100.00%	4	6	6	1.86%	55.70%	TAVCDIPPR	K	G	95.00%	66.1	38.1	33.5	0	1	0	0	2	1,028.52	351	359	FALSE	IPI01020686,IPI01022164,IPI01019113
20120614l	law	law3	law3 (F098	Tubulin beta-2C chain	IPI0000775	IPI	human	49,830.70	100.00%	4	6	6	1.86%	55.70%	YLTVAAVFR	R	G	95.00%	61.6	38	42.1	0	1	0	0	2	1,039.59	310	318	FALSE	IPI01020686,IPI01022164,IPI01019113
20120614l	law	law3	law3 (F098	Vimentin	IPI0041847	IPI	human	53,652.70	100.00%	8	8	8	2.48%	17.80%	ILLAELEQLK	K	G	95.00%	59.2	38.1	11.3	0	1	0	0	2	1,169.71	130	139	TRUE	
20120614l	law	law3	law3 (F098	Vimentin	IPI0041847	IPI	human	53,652.70	100.00%	8	8	8	2.48%	17.80%	ILLAELEQLKGQGK	K	S	95.00%	42.3	37.8	30	0	0	1	0	2	1,539.91	130	143	TRUE	
20120614l	law	law3	law3 (F098	Vimentin	IPI0041847	IPI	human	53,652.70	100.00%	8	8	8	2.48%	17.80%	ISLPLPNFSSLNLR	R	E	95.00%	90.6	38.2	75.2	0	1	0	0	2	1,570.90	411	424	TRUE	
20120614l	law	law3	law3 (F098	Vimentin	IPI0041847	IPI	human	53,652.70	100.00%	8	8	8	2.48%	17.80%	KVESLQEEIAFLK	R	K	95.00%	37.8	38.1	26	0	1	0	0	2	1,533.85	223	235	TRUE	
20120614l	law	law3	law3 (F098	Vimentin	IPI0041847	IPI	human	53,652.70	100.00%	8	8	8	2.48%	17.80%	NLQEAEEWYK	K	S	93.60%	34.9	38.1	27.5	0	1	0	0	2	1,309.61	283	292	TRUE	
20120614l	law	law3	law3 (F098	Vimentin	IPI0041847	IPI	human	53,652.70	100.00%	8	8	8	2.48%	17.80%	QDVDNASLAR	R	L	95.00%	79.8	38.9	56.4	0	1	0	0	2	1,088.53	208	217	TRUE	
20120614l	law	law3	law3 (F098	Vimentin	IPI0041847	IPI	human	53,652.70	100.00%	8	8	8	2.48%	17.80%	SLYASSPGGVYATR	R	S	95.00%	46.2	38.2	40.7	0	1	0	0	2	1,428.71	51	64	TRUE	
20120614l	law	law3	law3 (F098	Vimentin	IPI0041847	IPI	human	53,652.70	100.00%	8	8	8	2.48%	17.80%	SYVTTSTR	R	T	94.00%	34.8	37.8	29.4	0	1	0	0	2	914.4577	29	36	TRUE	
20120614l	law	law3	law3 (F098	Isoform 1 of CUB domain-containing protein 1	IPI0029003	IPI	human	92,932.90	100.00%	8	8	8	2.48%	9.09%	AFMIIQEQR	R	T	95.00%	44.1	38.4	25	0	1	0	0	2	1,135.59	604	612	TRUE	
20120614l	law	law3	law3 (F098	Isoform 1 of CUB domain-containing protein 1	IPI0029003	IPI	human	92,932.90	100.00%	8	8	8	2.48%	9.09%	ISFLCDDLTR	K	L	95.00%	50.9	38	28.2	0	1	0	0	2	1,239.60	397	406	TRUE	
20120614l	law	law3	law3 (F098	Isoform 1 of CUB domain-containing protein 1	IPI0029003	IPI	human	92,932.90	100.00%	8	8	8	2.48%	9.09%	IYVVDLSNER	K	A	92.30%	34.3	38.4	26	0	1	0	0	2	1,207.63	344	353	TRUE	
20120614l	law	law3	law3 (F098	Isoform 1 of CUB domain-containing protein 1	IPI0029003	IPI	human	92,932.90	100.00%	8	8	8	2.48%	9.09%	LATEEPPPR	K	S	95.00%	40.1	38.1	24.2	0	1	0	0	2	1,009.53	788	796	TRUE	
20120614l	law	law3	law3 (F098	Isoform 1 of CUB domain-containing protein 1	IPI0029003	IPI	human	92,932.90	100.00%	8	8	8	2.48%	9.09%	LSLVLVPAQK	R	L	95.00%	64.6	37.9	31.2	0	1	0	0	2	1,067.68	457	466	TRUE	
20120614l	law	law3	law3 (F098	Isoform 1 of CUB domain-containing protein 1	IPI0029003	IPI	human	92,932.90	100.00%	8	8	8	2.48%	9.09%	QGLTVSFIPYFK	R	E	95.00%	57.6	38.1	50.3	0	1	0	0	2	1,399.76	531	542	TRUE	
20120614l	law	law3	law3 (F098	Isoform 1 of CUB domain-containing protein 1	IPI0029003	IPI	human	92,932.90	100.00%	8	8	8	2.48%	9.09%	SGVVCQTGR	R	A	95.00%	67.5	38.7	44.2	0	1	0	0	2	963.4677	595	603	TRUE	
20120614l	law	law3	law3 (F098	Isoform 1 of CUB domain-containing protein 1	IPI0029003	IPI	human	92,932.90	100.00%	8	8	8	2.48%	9.09%	TFIWDVK	R	A	93.20%	34.7	38.3	11.6	0	1	0	0	2	908.4877	124	130	TRUE	
20120614l	law	law4	law4 (F098	Isoform 1 of CUB domain-containing protein 1	IPI0029003	IPI	human	92,932.90	100.00%	7	7	7	3.47%	8.25%	AFMIIQEQR	R	T	95.00%	51.8	38.4	31.3	0	1	0	0	2	1,135.59	604	612	TRUE	
20120614l	law	law4	law4 (F098	Isoform 1 of CUB domain-containing protein 1	IPI0029003	IPI	human	92,932.90	100.00%	7	7	7	3.47%	8.25%	DQVACLTFFK	R	E	95.00%	76.1	38.3	48.1	0	1	0	0	2	1,228.60	583	592	TRUE	
20120614l	law	law4	law4 (F098	Isoform 1 of CUB domain-containing protein 1	IPI0029003	IPI	human	92,932.90	100.00%	7	7	7	3.47%	8.25%	EEGVFTVTPDTK	K	S	95.00%	60.5	38.1	48.1	0	1	0	0	2	1,322.65	543	554	TRUE	
20120614l	law	law4	law4 (F098	Isoform 1 of CUB domain-containing protein 1	IPI0029003	IPI	human	92,932.90	100.00%	7	7	7	3.47%	8.25%	ISFLCDDLTR	K	L	95.00%	43.6	38	36.1	0	1	0	0	2	1,239.60	397	406	TRUE	
20120614l	law	law4	law4 (F098	Isoform 1 of CUB domain-containing protein 1	IPI0029003	IPI	human	92,932.90	100.00%	7	7	7	3.47%	8.25%	LATEEPPPR	K	S	95.00%	48.4	38.1	29.1	0	1	0	0	2	1,009.53	788	796	TRUE	
20120614l	law	law4	law4 (F098	Isoform 1 of CUB domain-containing protein 1	IPI0029003	IPI	human	92,932.90	100.00%	7	7	7	3.47%	8.25%	LSLVLVPAQK	R	L	95.00%	60.2	37.9	26.3	0	1	0	0	2	1,067.68	457	466	TRUE	
20120614l	law	law4	law4 (F098	Isoform 1 of CUB domain-containing protein 1	IPI0029003	IPI	human	92,932.90	100.00%	7	7	7	3.47%	8.25%	SGVVCQTGR	R	A	95.00%	51.6	38.7	37.5	0	1	0	0	2	963.4677	595	603	TRUE	
20120614l	law	law4	law4 (F098	Tubulin alpha-1B chain	IPI0093068	IPI	human	50,151.70	99.80%	2	2	2	0.99%	6.43%	AVFVDLEPTVIDEVR	R	T	95.00%	52	37.8	37.2	0	1	0	0	2	1,701.91	65	79	TRUE	IPI00180675,IPI01022358
20120614l	law	law4	law4 (F098	Tubulin alpha-1B chain	IPI0093068	IPI	human	50,151.70	99.80%	2	2	2	0.99%	6.43%	LISQIVSSITASLR	R	F	95.00%	43.1	38.3	32.5	0	1	0	0	2	1,487.88	230	243	TRUE	
20120614l	law	law4	law4 (F098	Keratin, type II cytoskeletal 1	IPI0022032	IPI	human	66,040.30	100.00%	3	3	3	1.49%	5.28%	FLEQQNQVLQTK	R	W	95.00%	112	38.3	75.1	0	1	0	0	2	1,475.79	200	211	TRUE	
20120614l	law	law4	law4 (F098	Keratin, type II cytoskeletal 1	IPI0022032	IPI	human	66,040.30	100.00%	3	3	3	1.49%	5.28%	SLNNQFASFIDK	K	V	95.00%	70.4	38.1	53.7	0	1	0	0	2	1,383.69	186	197	TRUE	
20120614l	law	law4	law4 (F098	Keratin, type II cytoskeletal 1	IPI0022032	IPI	human	66,040.30	100.00%	3	3	3	1.49%	5.28%	YEELQITAGR	K	H	95.00%	44.9	38.4	0	0	1	0	0	2	1,179.60	377	386	TRUE	
20120614l	law	law4	law4 (F098	Heterogeneous nuclear ribonucleoprotein F	IPI0000388	IPI	human	45,671.90	99.90%	2	2	2	0.99%	6.27%	QSGEAFVELGSEDDVK	R	M	95.00%	75.2	37.9	63.8	0	1	0	0	2	1,709.79	53	68	TRUE	

20120614l	law	law4	law4 (F098	Heterogeneous nuclear ribonucleoprotein F	IPI0000388	IPI	human	45,671.90	99.90%	2	2	2	0.99%	6.27%	VHIEIGPDGR	R	V	95.00%	44.7	38.5	36.6	0	1	0	0	2	1,092.58	317	326	TRUE	
20120614l	law	law4	law4 (F098	Actin, cytoplasmic 1	IPI000214	IPI	human	41,737.80	100.00%	4	4	4	1.98%	12.50%	AGFAGDDAPR	K	A	94.90%	40.2	38.8	19.7	0	1	0	0	2	976.4484	19	28	TRUE	
20120614l	law	law4	law4 (F098	Actin, cytoplasmic 1	IPI000214	IPI	human	41,737.80	100.00%	4	4	4	1.98%	12.50%	DSYVGDEAQSK	K	R	95.00%	68.8	38	49.4	0	1	0	0	2	1,198.52	51	61	TRUE	
20120614l	law	law4	law4 (F098	Actin, cytoplasmic 1	IPI000214	IPI	human	41,737.80	100.00%	4	4	4	1.98%	12.50%	GYSFTTTAER	R	E	95.00%	42.3	38.6	33.5	0	1	0	0	2	1,132.53	197	206	TRUE	
20120614l	law	law4	law4 (F098	Actin, cytoplasmic 1	IPI000214	IPI	human	41,737.80	100.00%	4	4	4	1.98%	12.50%	SYELPDGQVITIGNER	K	F	95.00%	62	37.7	49.8	0	1	0	0	2	1,790.89	239	254	TRUE	
20120614l	law	law4	law4 (F098	Keratin, type I cytoskeletal 10	IPI000098	IPI	human	58,828.80	100.00%	4	4	4	1.98%	7.02%	ALEESNYELEGK	R	I	95.00%	76.7	38	60.4	0	1	0	0	2	1,381.65	166	177	TRUE	
20120614l	law	law4	law4 (F098	Keratin, type I cytoskeletal 10	IPI000098	IPI	human	58,828.80	100.00%	4	4	4	1.98%	7.02%	DAEAWFNEK	K	S	93.20%	37.3	37.8	20.9	0	1	0	0	2	1,109.49	335	343	TRUE	
20120614l	law	law4	law4 (F098	Keratin, type I cytoskeletal 10	IPI000098	IPI	human	58,828.80	100.00%	4	4	4	1.98%	7.02%	SQYEQLAEQNR	R	K	95.00%	65.8	38.2	52.6	0	1	0	0	2	1,365.64	323	333	TRUE	
20120614l	law	law4	law4 (F098	Keratin, type I cytoskeletal 10	IPI000098	IPI	human	58,828.80	100.00%	4	4	4	1.98%	7.02%	VLDELTLTK	R	A	95.00%	49.1	38.8	34.9	0	1	0	0	2	1,031.60	258	266	TRUE	
20120614l	law	law4	law4 (F098	Keratin, type I cytoskeletal 18	IPI005547	IPI	human	48,059.00	100.00%	7	7	7	3.47%	15.10%	AQIFANTVDNAR	R	I	95.00%	93.4	38.4	77.5	0	1	0	0	2	1,319.67	138	149	TRUE	
20120614l	law	law4	law4 (F098	Keratin, type I cytoskeletal 18	IPI005547	IPI	human	48,059.00	100.00%	7	7	7	3.47%	15.10%	AQYDELAR	R	K	95.00%	43.3	38.3	15.4	0	1	0	0	2	965.4687	254	261	TRUE	
20120614l	law	law4	law4 (F098	Keratin, type I cytoskeletal 18	IPI005547	IPI	human	48,059.00	100.00%	7	7	7	3.47%	15.10%	ASLENSLR	K	E	95.00%	52.9	39.2	30.8	0	1	0	0	2	889.4737	318	325	TRUE	
20120614l	law	law4	law4 (F098	Keratin, type I cytoskeletal 18	IPI005547	IPI	human	48,059.00	100.00%	7	7	7	3.47%	15.10%	ETMQSLNDR	K	L	95.00%	44.1	37.8	28.2	0	1	0	0	2	1,109.49	82	90	TRUE	
20120614l	law	law4	law4 (F098	Keratin, type I cytoskeletal 18	IPI005547	IPI	human	48,059.00	100.00%	7	7	7	3.47%	15.10%	IREHLEK	K	K	95.00%	39.7	37.7	6.63	0	1	0	0	2	924.5262	112	118	TRUE	
20120614l	law	law4	law4 (F098	Keratin, type I cytoskeletal 18	IPI005547	IPI	human	48,059.00	100.00%	7	7	7	3.47%	15.10%	IVLQIDNAR	R	L	95.00%	53.6	37.9	28.3	0	1	0	0	2	1,041.61	150	158	TRUE	
20120614l	law	law4	law4 (F098	Keratin, type I cytoskeletal 18	IPI005547	IPI	human	48,059.00	100.00%	7	7	7	3.47%	15.10%	QAQEYEALLNIK	R	V	95.00%	86.2	38.4	66.7	0	1	0	0	2	1,402.72	359	370	TRUE	
20120614l	law	law4	law4 (F098	Elongation factor 1-alpha 1	IPI003964	IPI	human	50,141.20	100.00%	9	9	9	4.46%	18.00%	EHALLAYTLGVK	R	Q	95.00%	65.9	38.1	52.1	0	1	0	0	2	1,314.74	135	146	TRUE	
20120614l	law	law4	law4 (F098	Elongation factor 1-alpha 1	IPI003964	IPI	human	50,141.20	100.00%	9	9	9	4.46%	18.00%	EVSTYIK	K	K	95.00%	41.2	37.4	21.5	0	1	0	0	2	839.4509	173	179	TRUE	
20120614l	law	law4	law4 (F098	Elongation factor 1-alpha 1	IPI003964	IPI	human	50,141.20	100.00%	9	9	9	4.46%	18.00%	IGGIGTVPVGR	K	V	95.00%	67.2	38	45.5	0	1	0	0	2	1,025.61	256	266	TRUE	
20120614l	law	law4	law4 (F098	Elongation factor 1-alpha 1	IPI003964	IPI	human	50,141.20	100.00%	9	9	9	4.46%	18.00%	LPLQDVYK	R	I	95.00%	42	39.2	1.47	0	1	0	0	2	975.5511	248	255	TRUE	
20120614l	law	law4	law4 (F098	Elongation factor 1-alpha 1	IPI003964	IPI	human	50,141.20	100.00%	9	9	9	4.46%	18.00%	QTVAVGVIK	R	A	95.00%	41.7	37.8	26.7	0	1	0	0	2	914.567	431	439	TRUE	
20120614l	law	law4	law4 (F098	Elongation factor 1-alpha 1	IPI003964	IPI	human	50,141.20	100.00%	9	9	9	4.46%	18.00%	RYEEIVK	K	E	95.00%	46.6	38.1	15.7	0	1	0	0	2	936.5149	166	172	TRUE	
20120614l	law	law4	law4 (F098	Elongation factor 1-alpha 1	IPI003964	IPI	human	50,141.20	100.00%	9	9	9	4.46%	18.00%	STTTGHLIYK	K	C	95.00%	45.4	38.9	30.8	0	1	0	0	2	1,120.60	21	30	TRUE	
20120614l	law	law4	law4 (F098	Elongation factor 1-alpha 1	IPI003964	IPI	human	50,141.20	100.00%	9	9	9	4.46%	18.00%	TIEKFEK	R	E	95.00%	41.1	38.3	0	0	1	0	0	2	894.4932	38	44	TRUE	
20120614l	law	law4	law4 (F098	Elongation factor 1-alpha 1	IPI003964	IPI	human	50,141.20	100.00%	9	9	9	4.46%	18.00%	YYVTIIDAPGHR	K	D	95.00%	62.8	38.2	45.6	0	0	1	0	2	1,404.73	85	96	TRUE	
20120614l	law	law4	law4 (F098	DnaJ homolog subfamily A member 1	IPI000125	IPI	human	44,868.60	100.00%	4	5	5	2.48%	12.80%	QISQAYEVLSDAK	K	K	95.00%	106	38	91.9	0	2	0	0	2	1,451.74	47	59	TRUE	
20120614l	law	law4	law4 (F098	DnaJ homolog subfamily A member 1	IPI000125	IPI	human	44,868.60	100.00%	4	5	5	2.48%	12.80%	TIVITSHPGQIVK	R	H	95.00%	59.6	38.1	42.1	0	1	0	0	2	1,392.82	284	296	TRUE	
20120614l	law	law4	law4 (F098	DnaJ homolog subfamily A member 1	IPI000125	IPI	human	44,868.60	100.00%	4	5	5	2.48%	12.80%	VNFPENGFLSPDK	K	L	95.00%	48.1	38.3	38	0	1	0	0	2	1,463.72	326	338	TRUE	
20120614l	law	law4	law4 (F098	DnaJ homolog subfamily A member 1	IPI000125	IPI	human	44,868.60	100.00%	4	5	5	2.48%	12.80%	YHPDKNPNEGEK	K	F	95.00%	35.6	38.3	19.1	0	0	1	0	2	1,427.66	33	44	TRUE	
20120614l	law	law4	law4 (F098	elongation factor Tu, mitochondrial precursor	IPI000271	IPI	human	49,875.80	100.00%	5	5	5	2.48%	13.40%	AEAGDNLGALVR	R	G	95.00%	98.8	38.4	53.1	0	1	0	0	2	1,185.62	319	330	TRUE	
20120614l	law	law4	law4 (F098	elongation factor Tu, mitochondrial precursor	IPI000271	IPI	human	49,875.80	100.00%	5	5	5	2.48%	13.40%	ELLTEFGYK	R	G	95.00%	58.4	38.1	44.7	0	1	0	0	2	1,099.57	204	212	TRUE	
20120614l	law	law4	law4 (F098	elongation factor Tu, mitochondrial precursor	IPI000271	IPI	human	49,875.80	100.00%	5	5	5	2.48%	13.40%	GITINAAHVEYSTAAR	R	H	95.00%	38.8	37.9	27	0	0	1	0	2	1,673.86	108	123	TRUE	
20120614l	law	law4	law4 (F098	elongation factor Tu, mitochondrial precursor	IPI000271	IPI	human	49,875.80	100.00%	5	5	5	2.48%	13.40%	GTVVTGTLER	R	G	93.40%	38.6	39	14.1	0	1	0	0	2	1,032.57	275	284	TRUE	
20120614l	law	law4	law4 (F098	elongation factor Tu, mitochondrial precursor	IPI000271	IPI	human	49,875.80	100.00%	5	5	5	2.48%	13.40%	LLDAVDTYIPVPAR	K	D	95.00%	94.7	38.2	77.5	0	1	0	0	2	1,542.85	242	255	TRUE	
20120614l	law	law5	law5 (F098	Isoform 1 of CUB domain-containing protein 1	IPI002900	IPI	human	92,932.90	99.90%	2	2	2	1.12%	2.27%	LATEEPPPR	K	S	95.00%	47.3	38.1	30.4	0	1	0	0	2	1,009.53	788	796	TRUE	
20120614l	law	law5	law5 (F098	Isoform 1 of CUB domain-containing protein 1	IPI002900	IPI	human	92,932.90	99.90%	2	2	2	1.12%	2.27%	LSLVLVPAQK	R	L	95.00%	66.3	37.9	32.2	0	1	0	0	2	1,067.68	457	466	TRUE	
20120614l	law	law5	law5 (F098	Tubulin beta chain	IPI010191	IPI	human	49,670.60	66.70%	1	1	1	0.56%	2.03%	YLTVAAVFR	R	G	94.00%	37	38	17.6	0	1	0	0	2	1,039.59	310	318	TRUE	IPI00007752
20120614l	law	law5	law5 (F098	14-3-3 protein zeta/delta	IPI000212	IPI	human	27,745.90	99.80%	2	2	2	1.12%	10.60%	DICNDVLSLLEK	R	F	95.00%	52.5	38.5	35.2	0	1	0	0	2	1,418.72	92	103	TRUE	
20120614l	law	law5	law5 (F098	14-3-3 protein zeta/delta	IPI000212	IPI	human	27,745.90	99.80%	2	2	2	1.12%	10.60%	SVTEQGAELSNEER	K	N	95.00%	107	38.2	95.2	0	1	0	0	2	1,548.71	28	41	TRUE	
20120614l	law	law5	law5 (F098	40S ribosomal protein S3	IPI000112	IPI	human	26,688.60	99.80%	2	2	2	1.12%	9.05%	AELNEFLTR	K	E	95.00%	54.7	38.5	36.4	0	1	0	0	2	1,092.57	19	27	TRUE	
20120614l	law	law5	law5 (F098	40S ribosomal protein S3	IPI000112	IPI	human	26,688.60	99.80%	2	2	2	1.12%	9.05%	ELAEDGYSGVEVR	R	V	95.00%	78.2	38	65.3	0	1	0	0	2	1,423.67	28	40	TRUE	
20120614l	law	law5	law5 (F098	Mitochondrial 2-oxoglutarate/malate carrier protein	IPI002197	IPI	human	34,063.20	99.90%	2	2	2	1.12%	7.01%	LTGADGTPPGFLLK	R	A	95.00%	55.3	38.3	38.8	0	1	0	0	2	1,386.76	109	122	TRUE	
20120614l	law	law5	law5 (F098	Mitochondrial 2-oxoglutarate/malate carrier protein	IPI002197	IPI	human	34,063.20	99.90%	2	2	2	1.12%	7.01%	NGLDVLFK	K	V	95.00%	45.6	38.7	26	0	1	0	0	2	906.4933	261	268	TRUE	
20120614l	law	law5	law5 (F098	ADP/ATP translocase 2	IPI000071	IPI	human	32,853.50	100.00%	9	10	10	5.62%	25.50%	AAYFGIYDTAK	R	G	95.00%	79	38.5	71.1	0	1	0	0	2	1,219.60	189	199	TRUE	
20120614l	law	law5	law5 (F098	ADP/ATP translocase 2	IPI000071	IPI	human	32,853.50	100.00%	9	10	10	5.62%	25.50%	EQGVLSFWR	K	G	95.00%	57.8	38.6	45	0	1	0	0	2	1,121.57	64	72	TRUE	IPI00291467
20120614l	law	law5	law5 (F098	ADP/ATP translocase 2	IPI000071	IPI	human	32,853.50	100.00%	9	10	10	5.62%	25.50%	GAWSNVLR	K	G	95.00%	45.2	39.4	16.8	0	1	0	0	2	902.4842	273	280	TRUE	IPI00291467
20120614l	law	law5	law5 (F098	ADP/ATP translocase 2	IPI000071	IPI	human	32,853.50	100.00%	9	10	10	5.62%	25.50%	GNLANVIR	R	Y	95.00%	46.7	37.5	26.8	0	1	0	0	2	856.4999	73	80	TRUE	IPI00291467
20120614l	law	law5	law5 (F098	ADP/ATP translocase 2	IPI000071	IPI	human	32,853.50	100.00%	9	10	10	5.62%	25.50%	LLLQVQHASK	K	Q	95.00%	56.8	38.3	38.5	0	1	0	0	2	1,136.68	34	43	TRUE	IPI00291467
20120614l	law	law5	law5 (F098	ADP/ATP translocase 2	IPI000071	IPI	human	32,853.50	100.00%	9	10	10	5.62%	25.50%	QIFLGGVDK	K	R	95.00%	67	38.8	34.4	0	1	0	0	2	976.5464	97	105	TRUE	IPI00291467
20120614l	law	law5	law5 (F098	ADP/ATP translocase 2	IPI000071	IPI	human	32,853.50	100.00%	9	10	10	5.62%	25.50%	QIFLGGVDKR	K	T	95.00%	64.7	38.4	34.5	0	2	0	0	2	1,115.62	97	106	TRUE	
20120614l	law	law5	law5 (F098	ADP/ATP translocase 2	IPI000071	IPI	human	32,853.50	100.00%	9	10	10	5.62%	25.50%	TAVAPIER	K	V	95.00%	58.1	37.5	32.8	0	1	0	0	2	856.4887	24	31	TRUE	IPI00291467
20120614l	law	law5	law5 (F098	ADP/ATP translocase 2	IPI000071	IPI	human	32,853.50	100.00%	9	10	10	5.62%	25.50%	YFPTQALNFAFK	R	D	95.00%	54.7	38.4	37.6	0	1	0	0	2	1,446.74	81	92	TRUE	IPI00291467
20120614l	law	law5	law5 (F098	Isoform A of Phosphate carrier protein, mitochondrial	IPI000222	IPI	human	40,095.90	100.00%	7	8	8	4.49%	14.10%	EKGSSASLVLK	K	R	89.60%	34.7	38.7	21.9	0	1	0	0	2	1,118.64	294	304	TRUE	
20120614l	law	law5	law5 (F098	Isoform A of Phosphate carrier protein, mitochondrial	IPI000222	IPI	human	40,095.90	100.00%	7	8	8	4.49%	14.10%	FGFYEVFK	K	V	95.00%	48	38.3	37.1	0	1	0	0	2	1,036.51	138	145	TRUE	
20120614l	law	law5	law5 (F098	Isoform A of Phosphate carrier protein, mitochondrial	IPI000222	IPI	human	40,095.90	100.00%	7	8	8	4.49%	14.10%	GIFNGFSVTLK	K	E	90.00%	34.1	38	24.8	0	1	0	0	2	1,183.64	102	112	TRUE	
20120614l	law	law5	law5 (F098	Isoform A of Phosphate carrier protein, mitochondrial	IPI000222	IPI	human	40,095.90	100.00%	7	8	8	4.49%	14.10%	GSSASLVLK	K	R	95.00%	47.6	39.1	22.7	0	1	0	0	2	861.504	296	304	TRUE	
20120614l	law	law5	law5 (F098	Isoform A of Phosphate carrier protein, mitochondrial	IPI000222	IPI	human	40,095.90	100.00%	7	8	8	4.49%	14.10%	GSSASLVLKR	K	L	95.00%	75.6	39.2	52.1	0	1	0	0	2	1,017.61	296	305	TRUE	
20120614l	law	law5	law5 (F098	Isoform A of Phosphate carrier protein, mitochondrial	IPI000222	IPI	human	40,095.90	100.00%	7	8	8	4.49%	14.10%	IQTQPGYANTLR	R	D	95.00%	68.1	38.6	48	0	1	0	0	2	1,361.72	190	201	TRUE	
20120614l	law	law5	law5 (F098	Isoform A of Phosphate carrier protein, mitochondrial	IPI000222	IPI	human	40,095.90	100.00%	7	8	8	4.49%	14.10%	MYKEEGLK	K	A	95.00%	41.9	37.7	20.3	0	2	0	0	2	997.5024	207	214	TRUE	
20120614l	law	law5	law5 (F098	Keratin, type II cytoskeletal 1	IPI002203	IPI	human	66,040.30	70.80%	1	1	1	0.56%	1.86%	SLDLDSIIAEVK	R	A	95.00%	80.2	38.3	56.2	0	1	0	0	2	1,302.72	344	355	TRUE	
20120614l	law	law5	law5 (F098	Isoform 1 of Trans-2,3-enoyl-CoA reductase	IPI001006	IPI	human	36,036.00	100.00%	2	2	2	1.12%	7.14%	LPVGTTATLYFR	K	D	94.90%	38.1	38.1	32.4	0	1	0	0	2	1,338.74	68	79	TRUE	
20120614l	law	law5	law5 (F098	Isoform 1 of Trans-2,3-enoyl-CoA reductase	IPI001006	IPI	human	36,036.00	100.00%	2	2	2	1.12%	7.14%	SLKDEDVLQK	K	L	95.00%	56.5	38.7	26.6	0	1	0	0	2	1,174.63	58	67	TRUE	
20120614l	law	law5	law5 (F098	ADP/ATP translocase 3	IPI002914	IPI	human	32,867.10	99.80%	2	2	2	1.12%	27.90%	AAYFGVYDTAK	R	G	95.00%	85.1	38.7	77	0	1	0	0	2	1,205.58	189	199	TRUE	
20120614l	law	law5	law5 (F098	ADP/ATP translocase 3	IPI002914	IPI	human	32,867.10	99.80%	2	2	2	1.12%	27.90%	EQGVLSFWR	K	G	95.00%	57.8	38.6	45	0	1	0	0	2	1,121.57	64	72	FALSE	IPI00007188
20120614l	law	law5	law5 (F098	ADP/ATP translocase 3	IPI002914	IPI	human	32,867.10	99.80%	2	2	2	1.12%	27.90%	GAWSNVLR	K	G	95.00%	45.2	39.4	16.8	0	1	0	0	2	902.4842	273	280	FALSE	IPI00007188
20120614l	law	law5	law5 (F098	ADP/ATP translocase 3	IPI002914	IPI	human	32,867.10	99.80%	2	2	2	1.12%	27.90%	GNLANVIR	R	Y	95.00%	46.7	37.5	26.8	0	1	0	0	2	856.4999	73	80	FALSE	IPI00007188
20120614l	law	law5	law5 (F098	ADP/ATP translocase 3	IPI002914	IPI	human	32,867.10	99.80%	2	2	2	1.12%	27.90%	IFRDEGGK	K	A	95.00%	39.4	38.1	16.7	0	1	0	0	2	921.479	261	268	TRUE	
20120614l	law	law5	law5 (F098	ADP/ATP translocase 3	IPI002914	IPI	human	32,867.10	99.80%	2	2	2	1.12%	27.90%	LLLQVQHASK	K	Q	95.00%	56.8	38.3	38.5	0	1	0	0	2	1,136.68	34	43	FALSE	IPI00007188
20120614l	law	law5	law5 (F098	ADP/ATP translocase 3	IPI002914	IPI	human	32,867.10	99.80%	2	2	2	1.12%	27.90%	QIFLGGVDK	K	H	95.00%	67	38.8	34.4	0	1	0	0	2	976.5464	97	105	FALSE	IPI00007188
20120614l	law	law5	law5 (F098	ADP/ATP translocase 3	IPI002914	IPI	human	32,867.10	99.80%	2	2	2	1.12%	27.90%	TAVAPIER	K	V	95.00%	58.1	37.5	32.8	0	1	0	0	2	856.4887	24	31	FALSE	IPI00007188
20120614l	law	law5	law5 (F098	ADP/ATP translocase 3	IPI002914	IPI	human	32,867.10	99.80%	2	2	2	1.12%	27.90%	YFPTQALNFAFK	R	D	95.00%	54.7	38.4	37.6	0	1	0	0	2	1,446.74	81	92	FALSE	IPI00007188
20120614l	law	law5	law5 (F098	Tubulin alpha-1B chain	IPI009306	IPI	human	50,151.70	70.80%	1	1	1	0.56%	2.00%	EIIDLVLDR	K	I	95.00%	39.1	38.1	15.4	0	1	0	0	2	1,085.62	113	121	TRUE	IPI00180675,IPI01022358
20120614l	law	law6	law6 (F098	Tubulin alpha-1B chain	IPI009306	IPI	human	50,151.70	95.00%	1	1	1	5.26%	2.00%	EIIDLVLDR	K	I	95.00%	71.8	38.1	36.8	0	1	0	0	2	1,085.62	113	121	TRUE	IPI00180675,IPI01022358
20120614l	law	bsa	bsa (F0980	Serum albumin	IPI010284	IPI	bovin	69,294.20	100.00%	32	36	36	24.80%	51.40%	AEFVEVTK	K	L	95.00%	53.3	34.6	27.5	0	1	0	0	2	922.488	249	256	TRUE	
20120614l	law	bsa	bsa (F0980	Serum albumin	IPI010284	IPI	bovin	69,294.20	100.00%	32	36	36	24.80%	51.40%	ALKAWSVAR	R	L	95.00%	43.2	35.1	13	0	1	0	0	2	1,001.59	233	241	TRUE	
20120614l	law	bsa	bsa (F0980	Serum albumin	IPI010284	IPI	bovin	69,294.20	100.00%	32	36	36	24.80%	51.40%	CCAADDKEACFAVEGPK	K	L	95.00%	72.9	33.9	61.2	0	0	1	0	2	1,927.80	581	597	TRUE	
20120614l	law	bsa	bsa (F0980	Serum albumin	IPI010284	IPI	bovin	69,294.20	100.00%	32	36	36	24.80%	51.40%	CCTESLVNR	K	R	95.00%	73.2	34.2	61.3	0	1	0	0	2	1,138.50	499	507	TRUE	
20120614l	law	bsa	bsa (F0980	Serum albumin	IPI010284	IPI	bovin	69,294.20	100.00%	32	36	36	24.80%	51.40%	DAFLGSFLYEYSR	K	R	95.00%	93.1	34.2	84.7	0	1	0	0	2	1,567.74	347	359	TRUE	
20120614l	law	bsa	bsa (F0980	Serum albumin	IPI010284	IPI	bovin	69,294.20	100.00%	32	36	36	24.80%	51.40%	ETYGDMADCCEK	R	Q	95.00%	60.3	34.8	52.8	0	1	0	0	2	1,478.52	106	117	TRUE	
20120614l	law	bsa	bsa (F0980	Serum albumin	IPI010284	IPI	bovin	69,294.20	100.00%	32	36	36	24.80%	51.40%	EYEATLEECCAK	K	D	95.00%	70.2	34.7	59	0	1	0	0	2	1,502.61	375	386	TRUE	
20120614l	law	bsa	bsa (F0980	Serum albumin	IPI010284	IPI	bovin	69,294.20	100.00%	32	36	36	24.80%	51.40%	FKDLGEEHFK	R	G	95.00%	52.9	34.8	24.1	0	0	1	0	2	1,249.62	35	44	TRUE	
20120614l	law	bsa	bsa (F0980	Serum albumin	IPI010284	IPI	bovin	69,294.20	100.00%	32	36	36	24.80%	51.40%	HLVDEPQNLIK	K	Q	95.00%	67.1	34.4	57	0	1	0	0	2	1,305.72	402	412	TRUE	
20120614l	law	bsa	bsa (F0980	Serum albumin	IPI010284	IPI	bovin	69,294.20	100.00%	32	36	36	24.80%	51.40%	HPEYAVSVLLR	R	L	91.40%	33.6	34.1	15	0	1	0	0	2	1,283.71	361	371	TRUE	
20120614l	law	bsa	bsa (F0980	Serum albumin	IPI010284	IPI	bovin	69,294.20	100.00%	32	36	36	24.80%	51.40%	KQTALVELLK	K	H	93.60%	36	34.9	16.5	0	1	0	0	2	1,143.70	548	557	TRUE	
20120614l	law	bsa	bsa (F0980	Serum albumin	IPI010284	IPI	bovin	69,294.20	100.00%	32	36	36	24.80%	51.40%	KVPQVSTPTLVEVSR	R	S	95.00%	93.9	34.3	89.7	0	1	0	0	2	1,639.94	437	451	TRUE	
20120614l	law	bsa	bsa (F0980	Serum albumin	IPI010284	IPI	bovin	69,294.20	100.00%	32	36	36	24.80%	51.40%	LAKEYEATLEECCAK	R	D	95.00%	59.3	34.1	47	0	0	1	0	2	1,814.83	372	386	TRUE	
20120614l	law	bsa	bsa (F0980	Serum albumin	IPI010284	IPI	bovin	69,294.20	100.00%	32	36	36	24.80%	51.40%	LCVLHEKTPVSEK	R	V	95.00%	65.7	34.4	48.8	0	0	1	0	2	1,539.82	483	495	TRUE	
20120614l	law	bsa	bsa (F0980	Serum albumin	IPI010284	IPI	bovin	69,294.20	100.00%	32	36	36	24.80%	51.40%	LGEYGFQNALIVR	K	Y	95.00%	111	34.6	89.8	0	1	0	0	2	1,479.80	421	433	TRUE	
20120614l	law	bsa	bsa (F0980	Serum albumin	IPI010284	IPI	bovin	69,294.20	100.00%	32	36	36	24.80%	51.40%	LKECCDKPLLEK	K	S	95.00%	50.1	34.6	36.4	0	0	1	0	2	1,532.78	298	309	TRUE	
20120614l	law	bsa	bsa (F0980	Serum albumin	IPI010284	IPI	bovin	69,294.20	100.00%	32	36	36	24.80%	51.40%	LKPDPNTLCDEFK	K	A	93.00%	38	34.5	24.3	0	0	1	0	2	1,576.77	139	151	TRUE	
20120614l	law	bsa	bsa (F0980	Serum albumin	IPI010284	IPI	bovin	69,294.20	100.00%	32	36	36	24.80%	51.40%	LKPDPNTLCDEFKADEK	K	K	95.00%	52.7	33.7	42.7	0	0	1	0	2	2,019.97	139	155	TRUE	
20120614l	law	bsa	bsa (F0980	Serum albumin	IPI010284	IPI	bovin	69,294.20	100.00%	32	36	36	24.80%	51.40%	LSQKFPK	R	A	95.00%	41.3	35.4	18.9	0	1	0	0	2	847.5037	242	248	TRUE	
20120614l	law	bsa	bsa (F0980	Serum albumin	IPI010284	IPI	bovin	69,294.20	100.00%	32	36	36	24.80%	51.40%	LVNELTEFAK	K	T	95.00%	68	34.8	31.5	0	1	0	0	2	1,163.63	66	75	TRUE	
20120614l	law	bsa	bsa (F0980	Serum albumin	IPI010284	IPI	bovin	69,294.20	100.00%	32	36	36	24.80%	51.40%	LVVSTQTALA	K	-	95.00%	45.7	35.2	35.5	0	1	0	0	2	1,002.58	598	607	TRUE	
20120614l	law	bsa	bsa (F0980	Serum albumin	IPI010284	IPI	bovin	69,294.20	100.00%	32	36	36	24.80%	51.40%	QNCDQFEK	K	L	95.00%	55.2	34	28.2	0	2	0	0	2	1,068.44	413	420	TRUE	
20120614l	law	bsa	bsa (F0980	Serum albumin	IPI010284	IPI	bovin	69,294.20	100.00%	32	36	36	24.80%	51.40%	QTALVELLK	K	H	95.00%	65.5	34	28.8	0	2	0	0	2	997.593	549	557	TRUE	
20120614l	law	bsa	bsa (F0980	Serum albumin	IPI010284	IPI	bovin	69,294.20	100.00%	32	36	36	24.80%	51.40%	RHPEYAVSVLLR	R	L	95.00%	54.2	34.2	38.5	0	0	1	0	2	1,439.81	360	371	TRUE	
20120614l	law	bsa	bsa (F0980	Serum albumin	IPI010284	IPI	bovin	69,294.20	100.00%	32	36	36	24.80%	51.40%	RPCFSALTPDETYVPK	R	A	95.00%	45.1	34	24.9	0	0	1	0	2	1,880.92	508	523	TRUE	
20120614l	law	bsa	bsa (F0980	Serum albumin	IPI010284	IPI	bovin	69,294.20	100.00%	32	36	36	24.80%	51.40%	SHCIAEVEK	K	D	95.00%	48.3	35.4	27.3	0	1	0	0	2	1,072.51	310	318	TRUE	
20120614l	law	bsa	bsa (F0980	Serum albumin	IPI010284	IPI	bovin	69,294.20	100.00%	32	36	36	24.80%	51.40%	SLHTLFGDELCK	K	V	95.00%	67.6	34.7	55.4	0	1	1	0	2	1,419.69	89	100	TRUE	
20120614l	law	bsa	bsa (F0980	Serum albumin	IPI010284	IPI	bovin	69,294.20	100.00%	32	36	36	24.80%	51.40%	TCVADESHAGCEK	K	S	95.00%	54.7	34.6	24.3	0	0	1	0	2	1,463.59	76	88	TRUE	
20120614l	law	bsa	bsa (F0980	Serum albumin	IPI010284	IPI	bovin	69,294.20	100.00%	32	36	36	24.80%	51.40%	TVMENFVAFVDK	K	C	95.00%	83.2	34.5	71.7	0	2	0	0	2	1,415.69	569	580	TRUE	
20120614l	law	bsa	bsa (F0980	Serum albumin	IPI010284	IPI	bovin	69,294.20	100.00%	32	36	36	24.80%	51.40%	VPQVSTPTLVEVSR	K	S	95.00%	83.8	34.3	78.4	0	1	0	0	2	1,511.84	438	451	TRUE	
20120614l	law	bsa	bsa (F0980	Serum albumin	IPI010284	IPI	bovin	69,294.20	100.00%	32	36	36	24.80%	51.40%	YICDNQDTISSK	K	L	95.00%	96.9	34.6	81.2	0	1	0	0	2	1,443.64	286	297	TRUE	
20120614l	law	bsa	bsa (F0980	Serum albumin	IPI010284	IPI	bovin	69,294.20	100.00%	32	36	36	24.80%	51.40%	YLYEIAR	K	R	95.00%	43.7	33.7	0	0	1	0	0	2	927.4934	161	167	TRUE	

END OF FILE
