# Supplementary material for: CUB domain-containing protein 1 and the epidermal growth factor receptor cooperate to induce cell detachment
Source: Breast Cancer Res. 2016 Aug 5;18:80. doi: 10.1186/s13058-016-0741-1 (PMC4974783; doi:10.1186/s13058-016-0741-1)
Supplement: Additional file 2: — Summary of proteins identified by MS. (PDF 20 kb) [file 13058_2016_741_MOESM2_ESM.pdf]

	Accession Number	Molecular Mass	Total #of Unique Peptides	%Coverage
Tubulin beta chain	IPI01019113	50 kDa	24	56
Tubulin alpha-1B chain	IPI00930688	50 kDa	21	47
Isoform 1 of DNA-dependent protein kinase catalytic subunit	IPI00296337	469 kDa	14	4
Keratin, type II cytoskeletal 8	IPI00554648	54 kDa	10	20
Cytoplasmic dynein 1 heavy chain 1	IPI00456969	532 kDa	10	3
Keratin, type II cytoskeletal 1	IPI00220327	66 kDa	6	3
Keratin, type I cytoskeletal 18	IPI00554788	48 kDa	7	15
Isoform 1 of E3 ubiquitin-protein ligase TRIM21	IPI00018971	54 kDa	8	20
Vimentin	IPI00418471	54 kDa	8	18
Tubulin beta-2C chain	IPI00007752	50 kDa	4	56
Keratin, type I cytoskeletal 10	IPI00009865	59 kDa	5	7
Isoform 1 of Plectin	IPI00014898	532 kDa	2	1
p62/Isoform 1 of Sequestosome-1	IPI00179473	48 kDa	4	17
Enigma/Isoform 1 of PDZ and LIM domain protein 7	IPI00023122	50 kDa	4	10
Actin, cytoplasmic 1	IPI00021439	42 kDa	4	13
Keratin, type II cytoskeletal 7	IPI00306959	51 kDa	4	11
Matrix metalloproteinase-14	IPI00218398	66 kDa	2	13
14-3-3 protein zeta/delta	IPI00021263	28 kDa	2	11
Tubulin alpha-1A chain	IPI00180675	50 kDa	2	47
