# Supplementary material for: CUB domain-containing protein 1 and the epidermal growth factor receptor cooperate to induce cell detachment
Source: Breast Cancer Res. 2016 Aug 5;18:80. doi: 10.1186/s13058-016-0741-1 (PMC4974783; doi:10.1186/s13058-016-0741-1)
Supplement: Additional file 3: — Peptide sequence coverage. (PDF 1263 kb) [file 13058_2016_741_MOESM3_ESM.pdf]

IPI00296337 (100%), 469,095.5 Da
Isoform 1 of DNA-dependent protein kinase catalytic subunit
14 unique peptides, 14 unique spectra, 14 total spectra, 155/4128 amino acids (4% coverage)

MAGSGAGVRC	SLLR**LQETLS**	**AADR**CGAALA	GHQLIRGLGQ	ECVLSSSPAV	LALQTSLVFS	RDFGLLVFVR
KSLNSIEFRE	CREEILKFLC	IFLEKMGQKI	APYSVEIKNT	CTSVYTKDRA	AKCKIPALDL	LIKLLQTFRS
SRLMDEFKIG	ELFSKFYGEL	ALKKKIPDTV	LEKVYELLGL	LGEVHPSEMI	NNAENLFRAF	LGELKTQMTS
AVREPKLPVL	AGCLKGLSSL	LCNFTKSMEE	DPQTSREIFN	FVLKAIRPQI	DLKRYAVPSA	GLRLFALHAS
QFSTCLLDNY	VSLFEVLLKW	CAHTNVELKK	AALSALESFL	KQVSNMVAKN	AEMHKNKLQY	FMEQFYGIIR
NVDSNNKELS	IAIRGYGLFA	GPCKVINAKD	VDFMYVELIQ	RCKQMFLTQT	DTGDDRVYQM	PSFLQSVASV
LLYLDTVPEV	YTPVLEHLVV	MQIDSFPQYS	PKMQLVCCRA	IVKVFLALAA	KGPVLRNCIS	TVVHQGLIRI
CSKPVVLPKG	PESESEDHRA	SGEVRTGKWK	VPTYKDYVDL	FRHLLSSDQM	MDSILADEAF	FSVNSSSESL
NHLLYDEFVK	SVLKIVEKLD	LTLEIQTVGE	QENGDEAPGV	WMIPTSDPAA	NLHPAKPKDF	SAFINLVEFC
REILPEKQAE	FFEPWVYSFS	YELILQSTRL	PLISGFYKLL	SITVRNAKKI	KYFEGVSPKS	LKHSPEDPEK
YSCFALFVKF	GKEVAVKMKQ	YKDELLASCL	TFLLSLPHNI	IELDVRAYVP	ALQMAFKLGL	SYTPLAEVGL
NALEEWSIYI	DRHVMQPYYK	DILPCLDGYL	K**TSALSDETK**	NNWEVSALSR	AAQKGFNKVV	LKHLKKTKNL
SSNEAISLEE	IRIRVVQMLG	SLGGQINKNL	LTVTSSDEMM	KSYVAWDREK	RLSFAVPFRE	MKPVIFLDVF
LPRVTELALT	ASDRQTKVAA	CELLHSMVMF	MLGKATQMPE	GGQGAPPMYQ	LYKRTFPVLL	RLACDVDQVT
RQLYEPLVMQ	LIHWFTNNKK	FESQDTVALL	EAILDGIVDP	VDSTLRDFCG	RCIREFLKWS	IKQITPQQQE
KSPVNTKSLF	KRLYSLALHP	NAFKRLGASL	AFNNIYREFR	EEESLVEQFV	FEALVIYMES	LALAHADEKS
LGTIQQCCDA	IDHLCRIIEK	KHVSLNKAKK	RRLPRGFPPS	ASLCLLDLVK	WLLAHCGRPQ	TECRHKSIEL
FYKFVPLLPG	NRSPNLWLKD	VLKEEGVSFL	INTFEGGGCG	QPSGILAQPT	LLYLRGPFSL	QATLCWLDLL
LAALECYNTF	IGERTVGALQ	VLGTEAQSSL	LKAVAFFLES	IAMHDIIAAE	KCFGTGAAGN	RTSPQEGERY
NYSKCTVVVR	IMEFTTTLLN	TSPEGWKLLK	KDLCNTHLMR	VLVQTLCEPA	SIGFNIGDVQ	VMAHLPDVCV
NLMKALKMSP	YKDILETHLR	EKITAQSIEE	LCAVNLYGPD	AQVDRSRLAA	VVSACKQLHR	AGLLHNILPS
QSTDLHHSVG	TELLSLVYKG	IAPGDERQCL	PSLDLSCKQL	ASGLLELAFA	FGGLCERLVS	LLLNPAVLST
ASLGSSQGSV	IHFSHGEYFY	SLFSETINTE	LLKNLDLAVL	ELMQSSVDNT	KMVSAVLNGM	LDQSFRERAN
QKHQGLKLAT	TILQHWKKCD	SWWAKDSPLE	TKMAVLALLA	KILQIDSSVS	FNTSHGSFPE	VFTTYISLLA
DTKLDLHLKG	QAVTLLPFFT	SLTGGSLEEL	RRVLEQLIVA	HFPMQSREFP	PGTPRFNNYV	DCMKKFLDAL
ELSQSPMLLE	LMTEVLCREQ	QHVMEELFQS	SFRRIARRGS	CVTQVGLLES	VYEMFRKDDP	RLSFTRQSFV
DRSLLTLLWH	CSLDALREFF	STIVVDAIDV	LKSRFTK**LNE**	**STFDTQITK**K	MGYYKILDVM	YSR**LPKDDVH**
**AK**ESKINQVF	HGSCITEGNE	LTKTLIKLCY	DAFTENMAGE	NQLLERRRLY	HCAAYNCAIS	VICCVFNELK
FYQGFLFSEK	PEKNLLIFEN	LIDLKRRYNF	PVEVEVPMER	KKKYIEIRKE	AREAANGDSD	GPSYMSSLSY
LADSTLSEEM	SQFDFSTGVQ	SYSYSSQDPR	PATGRFRRRE	QRDPTVHDDV	LELEMDELNR	HECMAPLTAL
VKHMHR**SLGP**	**PQGEEDSVPR**	DLPSWMKFLH	GKLGNPIVPL	NIRLFLAKLV	INTEEVFRPY	AKHWLSPLLQ
LAASENNGGE	GIHYMVVEIV	ATILSWTGLA	TPTGVPKDEV	LANRLLNFLM	KHVFHPKRAV	FRHNLEIIKT
LVECWKDCLS	IPYRLIFEKF	SGKDPNSKDN	SVGIQLLGIV	MANDLPPYDP	QCGIQSSEYF	QALVNNMSFV
RYKEVYAAAA	EVLGLILRYV	MERKNILEES	LCELVAKQLK	QHQNTMEDKF	IVCLNKVTKS	FPPLADRFMN
AVFFLLPKFH	GVLKTLCLEV	VLCRVEGMTE	LYFQLKSKDF	VQVMRHRDDE	RQKVCLDIIY	KMMPKLKPVE
LRELLNPVVE	FVSHPSTTCR	EQMYNILMWI	HDNYRDPESE	TDNDSQEIFK	LAKDVLIQGL	IDENPGLQLI
IRNFWSHETR	LPSNTLDR**LL**	**ALNSLYSPK**I	EVHFLSLATN	FLLEMTSMSP	DYPNPMFEHP	LSECEFQEYT
IDSDWRFRST	VLTPMFVETQ	ASQGTLQTRT	QEGSLSARWP	VAGQIRATQQ	QHDFTLTQTA	DGRSSFDWLT
GSSTDPLVDH	TSPSSDSLLF	AHKRSERLQR	APLKSVGPDF	GKKRLGLPGD	EVDNKVKGAA	GRTDLLRLRR
RFMRDQEKLS	LMYARKGVAE	QKREKEIKSE	LKMKQDAQVV	LYRSYRHGDL	PDIQIKHSSL	ITPLQAVAQR
DPIIAKQLFS	SLFSGILKEM	DKFKTLSEKN	NITQKLLQDF	NRFLNTTFSF	FPPFVSCIQD	ISCQHAALLS
LDPAAVSAGC	LASLQQPVGI	R**LLEEALLR**L	LPAELPAKRV	RGKARLPPDV	LRWVELAKLY	RSIGEYDVLR
GIFTSEIGTK	**QITQSALLAE**	**AR**SDYSEAAK	QYDEALNKQD	WVDGEPTEAE	KDFWELASLD	CYNHLAEWKS
LEYCSTASID	SENPPDLNKI	WSEPFYQETY	LPYMIRSKLK	LLLQGEADQS	LLTFIDKAMH	GELQKAILEL
HYSQELSLLY	LLQDDVDRAK	YYIQNGIQSF	MQNYSSIDVL	LHQSRLTKLQ	SVQALTEIQE	FISFISKQGN
LSSQVPLKRL	LNTWTNRYPD	AKMDPMNIWD	DIITNRCFFL	SKIEEKLTPL	PEDNSMNVDQ	DGDPSDRMEV
QEQEEDISSL	IRSCKFSMKM	KMIDSARKQN	NFSLAMKLLK	ELHKESKTRD	DWLVSWVQSY	CRLSHCRSR**S**
**QGCSEQVLTV**	**LK**TVSLLDEN	NVSSYLSKNI	LAFR**DQNILL**	**GTTYR**IIANA	LSSEPACLAE	IEEDKARRIL
ELSGSSSEDS	EKVIAGLYQR	AFQHLSEAVQ	AAEEEAQPPS	WSCGPAAGVI	DAYMTLADFC	DQQLRKEEEN
ASVIDSAELQ	AYPALVVEKM	LKALKLNSNE	ARLKFPRLLQ	IIER**YPEETL**	**SLMTK**EISSV	PCWQFISWIS
HMVALLDKDQ	AVAVQHSVEE	ITDNYPQAIV	YPFIISSESY	SFKDTSTGHK	NKEFVARIKS	KLDQGGVIQD
FINALDQLSN	PELLFKDWSN	DVRAELAKTP	VNKKNIEKMY	ERMYAALGDP	KAPGLGAFRR	KFIQTFGKEF
DKHFGKGGSK	LLRMKLSDFN	DITNMLLLKM	NKDSKPPGNL	KECSPWMSDF	KVEFLR**NELE**	**IPGQYDGR**GK
PLPEYHVRIA	GFDERVTVMA	SLRRPKRIII	RGHDEREHPF	LVKGGEDLRQ	DQRVEQLFQV	MNGILAQDSA
CSQRALQLRT	YSVVPMTSRL	GLIEWLENTV	TLKDLLLNTM	SQEEKAAYLS	DPRAPPCEYK	DWLTKMSGKH
DVGAYMLMYK	GANR**TETVTS**	**FR**KRESKVPA	DLLKRAFVRM	STSPEAFLAL	RSHFASSHAL	ICISHWILGI
GDRHLNNFMV	AMETGGVIGI	DFGHAFGSAT	QFLPVPELMP	FRLTRQFINL	MLPMKETGLM	YSIMVHALRA
FRSDPGLLTN	TMDVFVKEPS	FDWKNFEQKM	LKKGGSWIQE	INVAEKNWYP	RQKICYAKRK	LAGANPAVIT
CDELLLGHEK	APAFRDYVAV	ARGSKDHNIR	**AQEPESGLSE**	**ETQVK**CLMDQ	ATDPNILGRT	WEGWEPWM

IPI00456969 (100%), 532,405.6 Da
Cytoplasmic dynein 1 heavy chain 1
10 unique peptides, 10 unique spectra, 10 total spectra, 119/4646 amino acids (3% coverage)

```
MSEPGGGGGE DGSAGLEVSA VQNVADVSVL QKHLRKLVPL LLEDGGEAPA ALEAALEEKS ALEQMRKFLS
DPQVHTVLVE RSTLKEDVGD EGEEEKEFIS YNINIDIHYG VKSNSLAFIK RTPVIDADKP VSSQLRVLTL
SEDSPYETLH SFISNAVAPF FKSYIRESGK ADRDGDKMAP SVEKKIAELE MGLLHLQQNI EIPEISLPIH
PMITNVAKQC YERGEKPKVT DFGDKVEDPT FLNQLQSGVN RWIREIQKVT KLDRDPASGT ALQEISFWLN
LERALYRIQE KRESPEVLLT LDILKHGKRF HATVSFDTDT GLKQALETVN DYNPLMKDFP LNDLLSATEL
DKIRQALVAI FTHLRKIRNT KYPIQRALRL VEAISRDLSS QLLKVLGTRK LMHVAYEEFE KVMVACFEVF
QTWDDEYEKL QVLLRDIVKR KREENLKMVW RINPAHRKLQ ARLDQMRKFR RQHEQLRAVI VRVLRPQVTA
VAQQNQGEVP EPQDMKVAEV LFDAADANAI EEVNLAYENV KEVDGLDVSK EGTEAWEAAM KRYDERIDRV
ETRITARLRD QLGTAKNANE MFRIFSRFNA LFVRPHIRGA IREYQTQLIQ RVKDDIESLH DKFKVQYPQS
QACKMSHVRD LPPVSGSIIW AKQIDRQLTA YMKRVEDVLG KGWENHVEGQ KLKQDGDSFR MKLNTQEIFD
DWARKVQQRN LGVSGRIFTI ESTRVRGRTG NVLKLKVNFL PEIITLSKEV RNLKWLGFRV PLAIVNKAHQ
ANQLYPFAIS LIESVRTYER TCEKVEERNT ISLLVAGLKK EVQALIAEGI ALVWESYKLD PYVQRLAETV
FNFQEKVDDL LIIEEKIDLE VRSLETCMYD HKTFSEILNR VQKAVDDLNL HSYSNLPIWV NKLDMEIERI
LGVRLQAGLR AWTQVLLGQA EDKAEVDMDT DAPQVSHKPG GEPKIKNVVH ELRITNQVIY LNPPIEECRY
KLYQEMFAWK MVVLSLPRIQ SQRYQVGVHY ELTEEEKFYR NALTRMPDGP VALEESYSAV MGIVSEVEQY
VKVWLQYQCL WDMQAENIYN RLGEDLNKWQ ALLVQIRKAR GTFDNAETKK EFGPVVIDYG KVQSKVNLKY
DSWHKEVLSK FGQMLGSNMT EFHSQISKSR QELEQHSVDT ASTSDAVTFI TYVQSLKRKI KQFEKQVELY
RNGQRLLEKQ RFQFPPSWLY IDNIEGEWGA FNDIMRRKDS AIQQQVANLQ MKIVQEDRAV ESRTTDLLTD
WEKTKPVTGN LRPEEALQAL TIYEGKFGRL KDDREKCAKA KEALELTDTG LLSGSEERVQ VALEELQDLK
GVWSELSKVW EQIDQMKEQP WVSVQPRKLR QNLDALLNQL KSFPARLRQY ASYEFVQRLL KGYMKINMLV
IELKSEALKD RHWKQLMKRL HVNWVVSELT LGQIWDVDLQ KNEAIVKDVL LVAQGEMALE EFLKQIREVW
NTYELDLVNY QNKCRLIRGW DDLFNKVKEH INSVSAMKLS PYYKVFEEDA LSWEDKLNRI MALFDVWIDV
QRRWVYLEGI FTGSADIKHL LPVETQRFQS ISTEFLALMK KVSKSPLVMD VLNIQGVQRS LERLADLLGK
IQKALGEYLE RERSSFPRFY FVGDEDLLEI IGNSKNVAKL QKHFKKMFAG VSSIILNEDN SVVLGISSRE
GEEVMFKTPV SITEHPKINE WLTLVEKEMR VTLAKLLAES VTEVEIFGKA TSIDPNTYIT WIDKYQAQLV
VLSAQIAWSE NVETALSSMG GGGDAAPLHS VLSNVEVTLN VLADSVLMEQ PPLRRRKLEH LITELVHQRD
VTRSLIKSKI DNAKSFEWLS QMRFYFDPKQ TDVLQQLSIQ MANAKFNYGF EYLGVQDKLV QTPLTDRCYL
TMTQALEARL GGSPFGPAGT GKTESVKALG HQLGRFVLVF NCDETFDFQA MGRIFVGLCQ VGAWGCFDEF
NRLEERMLSA VSQQVQCIQE ALREHSNPNY DKTSAPITCE LLNKQVKVSP DMAIFITMNP GYAGRSNLPD
NLKKLFRSLA MTKPDRQLIA QVMLYSQGFR TAEVLANKIV PFFKLCDEQL SSQSHYDFGL RALKSVLVSA
GNVKRERIQK IKREKEERGE AVDEGEIAEN LPEQEILIQS VCETMVPKLV AEDIPLLFSL LSDVFPGVQY
HRGEMTALRE ELKKVCQEMY LTYGDGEEVG GMWVEKVLQL YQITQINHGL MMVGPSGSGK SMAWRVLLKA
LERLEGVEGV AHIIDPKAIS KDHLYGTLDP NTREWTDGLF THVLRKIIDS VRGELQKRQW IVFDGDVDPE
WVENLNSVLD DNKLLTLPNG ERLSLPPNVR IMFEVQDLKY ATLATVSRCG MVWFSEDVLS TDMIFNNFLA
RLRSIPLDEG EDEAQRRRKG KEDEGEEAAS PMLQIQRDAA TIMQPYFTSN GLVTKALEHA FQLEHIMDLT
RLRCLGSLFS MLHQACRNVA QYNANHPDFP MQIEQLERYI QRYLVYAILW SLSGDSRLKM RAELGEYIRR
ITTVPLPTAP NIPIIDYEVS ISGEWSPWQA KVPQIEVETH KVAAPDVVVP TLDTVRHEAL LYTWLAEHKP
LVLCGPPGSG KTMTLFSALR ALPDMEVVGL NFSSATTPEL LLKTFDHYCE YRRTPNGVVL APVQLGKWLV
LFCDEINLPD MDKYGTQRVI SFIRQMVEHG GFYRTSDQTW VKLERIQFVG ACNPPTDPGR KPLSHRFLRH
VPVVYVDYPG PASLTQIYGT FNRAMLRLIP SLRTYAEPLT AAMVEFYTMS QERFTQDTQP HYIYSPREMT
RWVRGIFEAL RPLETLPVEG LIRIWAHEAL RLFQDRLVED EERRWTDENI DTVALKHFPN IDREKAMSRP
ILYSNWLSKD YIPVDQEELR DYVKARLKVF YEEELDVPLV LFNEVLDHVL RIDRIFRQPQ GHLLLIGVSG
AGKTTLSRFV AWMNGLSVYQ IKVHRKYTGE DFDEDLRTVL RRSGCKNEKI AFIMDESNVL DSGFLERMNT
LLANGEVPGL FEGDEYATLM TQCKEGAQKE GLMLDSHEEL YKWFTSQVIR NLHVVFTMNP SSEGLKDRAA
TSPALFNRCV LNWFGDWSTE ALYQVGKEFT SKMDLEKPNY IVPDYMPVVY DKLPQPPSHR EAIVNSCVFV
HQTLHQANAR LAKRGGRTMA ITPRHYLDFI NHYANLFHEK RSELEEQQMH LNVGLRKIKE TVDQVEELRR
DLRIKSQELE VKNAAANDKL KKMVKDQQEA EKKKVMSQEI QEQLHKQQEV IADKQMSVKE DLDKVEPAVI
EAQNAVKSIK KQHLVEVRSM ANPPAAVKLA LESICLLLGE STTDWKQIRS IIMRENFIPT IVNFSAEEIS
DAIREKMKKN YMSNPSYNYE IVNRASLACG PMVKWAIAQL NYADMLKRVE PLRNELQKLE DDAKDNQQKA
NEVEQMIRDL EASIARYKEE YAVLISEAQA IKADLAAVEA KVNRSTALLK SLSAERERWE KTSETFKNQM
STIAGDCLLS AAFIAYAGYF DQQMRQNLFT TWSHHLQQAN IQFRTDIART EYLSNADERL RWQASSLPAD
DLCTENAIML KRFNRYPLII DPSGQATEFI MNEYKDRKIT RTSFLDDAFR KNLESALRFG NPLLVQDVES
YDPVLNPVLN REVRRTGGRV LITLGDQDID LSPSFVIFLS TRDPTVEFPP DLCSRVTFVN FTVTRSSLQS
QCLNEVLKAE RPDVDEKRSD LLKLQGEFQL RLRQLEKSLL QALNEVKGRI LDDDTIITTL ENLKREAAEV
TRKVEETDIV MQEVETVSQQ YLPLSTACSS IYFTMESLKQ IHFLYQYSLQ FFLDIYHNVL YENPNLKGVT
DHTQRLSIIT KDLFQVAFNR VARGMLHQDH ITFAMLLARI KLKGTVGEPT YDAEFQHFLR GNEIVLSAGS
TPRIQGLTVE QAEAVVRLSC LPAFKDLIAK VQADEQFGIW LDSSSPEQTV PYLWSEETPA TPIGQAIHRL
LLIQAFRPDR LLAMAHMFVS TNLGESFMSI MEQPLDLTHI VGTEVKPNTP VLMCSVPGYD ASGHVEDLAA
EQNTQITSIA IGSAEGFNQA DKAINTAVKS GRWVMLKNVH LAPGWLMQLE KKLHSLQPHA CFRLFLTMEI
```

IPI00014898 (100%), 531,783.9 Da
Isoform 1 of Plectin
2 unique peptides, 2 unique spectra, 2 total spectra, 26/4684 amino acids (1% coverage)

MVAGMLMPRD QLRAIYEVLF REGVMVAKKD RRPRSLHPHV PGVTNLQVMR AMASLRARGL VRETFAWCHF
YWYLTNEGIA HLRQYLHLPP EIVPASLQRV RRPVAMVMPA RRTPHVQAVQ GPLGSPPKRG PLPTEEQRVY
RRKELEEVSP ETPVVPATTQ RTLARPGPEP APATDERDRV QKKTFTKWVN KHLIKAQRHI SDLYEDLRDG
HNLISLLEVL SGDSLPREKG RMRFHKLQNV QIALDYLRHR QVKLVNIRND DIADGNPKLT LGLIWTIILH
FQISDIQVSG QSEDMTAKEK LLLWSQRMVE GYQGLRCDNF TSSWRDGRLF NAIIHRHKPL LIDMNKVYRQ
TNLENLDQAF SVAERDLGVT RLLDPEDVDV PQPDEKSIIT YVSSLYDAMP RVPDVQDGVR ANELQLRWQE
YRELVLLLLQ WMRHHTAAFE ERRFPSSFEE IEILWSQFLK FKEMELPAKE ADKNRSKGIY QSLEGAVQAG
QLKVPPGYHP LDVEKEWGKL HVAILEREKQ LRSEFERLEC LQRIVTKLQM EAGLCEEQLN QADALLQSDV
RLLAAGKVPQ RAGEVERDLD KADSMIRLLF NDVQTLKDGR HPQGEQMYRR VYRLHERLVA IRTEYNLRLK
AGVAAPATQV AQVTLQSVQR RPELEDSTLR YLQDLLAWVE ENQHRVDGAE WGVDLPSVEA QLGSHRGLHQ
SIEEFRAKIE RARSDEGQLS PATRGAYRDC LGRLDLQYAK LLNSSKARLR SLESLHSFVA AATKELMWLN
EKEEEEVGFD WSDRNTNMTA KKESYSALMR ELELKEKKIK ELQNAGDRLL REDHPARPTV ESFQAALQTQ
WSWMLQLCCC IEAHLKENAA YFQFFSDVRE AEGQLQKLQE ALRRKYSCDR SATVTRLEDL LQDAQDEKEQ
LNEYKGHLSG LAKRAKAVVQ LKPRHPAHPM RGRLPLLAVC DYKQVEVTVH KGDECQLVGP AQPSHWKVLS
SSGSEAAVPS VCFLVPPPNQ EAQEAVTRLE AQHQALVTLW HQLHVDMKSL LAWQSLRRDV QLIRSWSLAT
FRTLKPEEQR QALHSLELHY QAFLRDSQDA GGFGPEDRLM AEREYGSCSH HYQQLLQSLE QGAQEESRCQ
RCISELKDIR LQLEACETRT VHRLRLPLDK EPARECAQRI AEQQKAQAEV EGLGKGVARL SAEAEKVLAL
PEPSPAAPTL RSELELTLGK LEQVRSLSAI YLEKLKTISL VIRGTQGAEE VLRAHEEQLK EAQAVPATLP
ELEATKASLK KLRAQAEAQQ PTFDALRDEL RGAQEVGERL QQRHGERDVE VERWRERVAQ LLERWQAVLA
QTDVRQRELE QLGRQLRYYR ESADPLGAWL QDARRRQEQI QAMPLADSQA VREQLRQEQA LLEEIERHGE
KVEECQRFAK QYINAIKDYE LQLVTYKAQL EPVASPAKKP KVQSGSESVI QEYVDLRTHY SELTTLTSQY
IKFISETLRR MEEEERLAEQ QRAEERERLA EVEAALEKQR QLAEAHAQAK AQAEREAKEL QQRMQEEVVR
REEAAVDAQQ QKRSIQEELQ QLRQSSEAEI QAKARQAEAA ERSRLRIEEE IRVVRLQLEA TERQRGGAEG
ELQALRARAE EAEAQKRQAQ EEAERLRRQV QDESQRKRQA EVELASRVKA EAEAAREKQR ALQALEELRL
QAEEAERRLR QAEVERARQV QVALETAQRS AEAELQSKRA SFAEKTAQLE RSLQEEHVAV AQLREEAERR
AQQQAEAERA REEAERELER WQLKANEALR LRLQAEEVAQ QKSLAQAEAE KQKEEAEREA RRRGKAEEQA
VRQRELAEQE LEKQRQLAEG TAQQRLAAEQ ELIRLRAETE QGEQQRQLLE EELARLQREA AAATQKRQEL
EAELAKVRAE MEVLLASKAR AEEESRSTSE KSKQRLEAEA GRFRELAEEA ARLRALAEEA KRQRQLAEED
AARQRAEAER VLAEKLAAIG EATRLKTEAE IALKEKEAEN ERLRRLAEDE AFQRRRLEEQ AAQHKADIEE
RLAQLRKASD SELERQKGLV EDTLRQRRQV EEEILALKAS FEKAAAGKAE LELELGRIRS NAEDTLRSKE
QAELEAARQR QLAAEEERRR REAEERVQKS LAAEEEAARQ RKAALEEVER LKAKVEEARR LRERAEQESA
RQLQLAQEAA QKRLQAEEKA HAFAVQQKEQ ELQQTLQQEQ SVLDQLRGEA EAARRAAEEA EEARVQAERE
AAQSRRQVEE AERLKQSAEE QAQARAQAQA AAEKLRKEAE QEAARRAQAE QAALRQKQAA DAEMEKHKKF
AEQTLRQKAQ VEQELTTLRL QLEETDHQKN LLDEELQRLK AEATEAARQR SQVEEELFSV RVQMEELSKL
KARIEAENRA LILRDKDNTQ RFLQEEAEKM KQVAEEAAR**L** **SVAAQEAAR**L RQLAEEDLAQ QRALAEKMLK
EKMQAVQEAT RLKAEAELLQ QQKELAQEQA RRLQEDKEQM AQQLAEETQG FQRTLEAERQ RQLEMSAEAE
RLKLRVAEMS RAQARAEEDA QRFRKQAEEI GEKLHRTELA TQEKVTLVQT LEIQRQQSDH DAERLREAIA
ELEREKEKLQ QEAKLLQLKS EEMQTVQQEQ LLQETQALQQ SFLSEKDSLL QRERFIEQEK AKLEQLFQDE
VAKAQQLREE QQRQQQQMEQ ERQRLVASME EARRRQHEAE EGVRRKQEEL QQLEQQRRQQ EELLAEENQR
LREQLQLLEE QHRAALAHSE EVTASQVAAT KTLPNGRDAL DGPAAEAEPE HSFDGLRRKV SAQRLQEAGI
LSAEELQRLA QGHTTVDELA RREDVRHYLQ GRSSIAGLLL KATNEKLSVY AALQRQLLSP GTALILLEAQ
AASGFLLDPV RNRRLTVNEA VKEGVVGPEL HHKLLSAERA VTGYKDPYTG QQISLFQAMQ KGLIVREHGI
RLLEAQIATG GVIDPVHSHR VPVDVAYRRG YFDEEMNRVL ADPSDDTKGF FDPNTHENLT YLQLLERCVE
DPETGLCLLP LTDKAAKGGE LVYTDSEARD VFEKATVSAP FGKFQGKTVT IWEIINSEYF TAEQRRDLLR
QFRTGRITVE KIIKIIITVV EEQEQKGRLC FEGLRSLVPA AELLESRVID RELYQQLQRG ERSVRDVAEV
DTVRRALRGA NVIAGVWLEE AGQKLSIYNA LKKDLLPSDM AVALLEAQAG TGHIIDPATS ARLTVDEAVR
AGLVGPEFHE KLLSAEKAVT GYRDPYTGQS VSLFQALKKG LIPREQGLR**L** **LDAQLSTGGI** **VDPSK**SHRVP
LDVACARGCL DEETSRALSA PRADAKAYSD PSTGEPATYG ELQQRCRPDQ LTGLSLLPLS EKAARARQEE
LYSELQARET FEKTPVEVPV GGFKGRTVTV WELISSEYFT AEQRQELLRQ FRTGKVTVEK VIKILITIVE
EVETLRQERL SFSGLRAPVP ASELLASGVL SRAQFEQLKD GKTTVKDLSE LGSVRTLLQG SGCLAGIYLE
DTKEKVSIYE AMRRGLLRAT TAALLLEAQA ATGFLVDPVR NQRLYVHEAV KAGVVGPELH EQLLSAEKAV
TGYRDPYSGS TISLFQAMQK GLVLRQHGIR LLEAQIATGG IIDPVHSHRV PVDVAYQRGY FSEEMNRVLA
DPSDDTKGFF DPNTHENLTY RQLLERCVED PETGLRLLPL KGAEKAEVVE TTQVYTEEET RRAFEETQID
IPGGGSHGGS TMSLWEVMQS DLIPEEQRAQ LMADFQAGRV TKERMIIIII EIIEKTEIIR QQGLASYDYV
RRRLTAEDLF EARIISLETY NLLREGTRSL REALEAESAW CYLYGTGSVA GVYLPGSRQT LSIYQALKKG
LLSAEVARLL LEAQAATGFL LDPVKGERLT VDEAVRKGLV GPELHDRLLS AERAVTGYRD PYTEQTISLF
QAMKKELIPT EEALRLLDAQ LATGGIVDPR LGFHLPLEVA YQRGYLNKDT HDQLSEPSEV RSYVDPSTDE
RLSYTQLLRR CRRDDGTGQL LLPLSDARKL TFRGLRKQIT MEELVRSQVM DEATALQLRE GLTSIEEVTK
NLQKFLEGTS CIAGVFVDAT KERLSVYQAM KKGIIRPGTA FELLEAQAAT GYVIDPIKGL KLTVEEAVRM
GIVGPEFKDK LLSAERAVTG YKDPYSGKLI SLFQAMKKGL ILKDHGIRLL EAQIATGGII DPEESHRLPV
EVAYKRGLFD EEMNEILTDP SDDTKGFFDP NTEENLTYLQ LMERCITDPQ TGLCLLPLKE KKRERKTSSK
SSVRKRRVVI VDPETGKEMS VYEAYRKGLI DHQTYLELSE QECEWEEITI SSSDGVVKSM IIDRRSGRQY
DIDDAIAKNL IDRSALDQYR AGTLSITEFA DMLSGNAGGF RSRSSSVGSS SSYPISPAVS RTQLASWSDP
TEETGPVAGI LDTETLEKVS ITEAMHRNLV DNITGQRLLE AQACTGGIID PSTGERFPVT DAVNKGLVDK
IMVDRINLAQ KAFCGFEDPR TKTKMSAAQA LKKGWLYYEA GQRFLEVQYL TGGLIEPDTP GRVPLDEALQ
RGTVDARTAQ KLRDVGAYSK YLTCPKTKLK ISYKDALDRS MVEEGTGLRL LEAAAQSTKG YYSPYSVSGS
GSTAGSRTGS RTGSRAGSRR GSFDATGSGF SMTFSSSSYS SSGYGRRYAS GSSASLGGPE SAVA

IPI00018971 (100%), 54,169.5 Da
Isoform 1 of E3 ubiquitin-protein ligase TRIM21
8 unique peptides, 8 unique spectra, 8 total spectra, 96/475 amino acids (20% coverage)

MASAARLTMM	WEEVTCPICL	DPFVEPVSIE	CGHSFCQECI	SQVGKGGGSV	CPVCRQRFLL	KNLRPNRQLA
NMVNNLKEIS	QEAREGTQGE	RCAVHGERLH	LFCEKDGKAL	CWVCAQSRKH	RDHAMVPLEE	AAQEYQEKLQ
VALGELRRKQ	ELAEKLEVEI	AIKRADWKKT	VETQKSRIHA	EFVQQKNFLV	EEEQRQLQEL	EKDEREQLRI
LGEKEAKLAQ	QSQALQELIS	ELDRRCHSSA	LELLQEVIIV	LERSESWNLK	DLDITSPELR	SVCHVPGLKK
MLRTCAVHIT	LDPDTANPWL	ILSEDRRQVR	LGDTQQSIPG	NEERFDSYPM	VLGAQHFHSG	KHYWEVDVTG
KEAWDLGVCR	DSVRRKGHFL	LSSKSGFWTI	WLWNKQKYEA	GTYPQTPLHL	QVPPCQVGIF	LDYEAGMVSF
YNITDHGSLI	YSFSECAFTG	PLRPFFSPGF	NDGGKNTAPL	TLCPLNIGSQ	GSTDY	

IPI00179473 (100%), 47,687.4 Da
Isoform 1 of Sequestosome-1
4 unique peptides, 4 unique spectra, 4 total spectra, 75/440 amino acids (17% coverage)

MASLTVKAYL	LGKEDAAREI	RRFSFCCSPE	PEAEAEAAAG	PGPCERLLSR	VAALFPALRP	GGFQAHYRDE
DGDLVAFSSD	EELTMAMSYV	KDDIFRIYIK	EKKECRRDHR	PPCAQEAPRN	MVHPNVICDG	CNGPVVGTRY
KCSVCPDYDL	CSVCEGKGLH	RGHTKLAFPS	PFGHLSEGFS	HSRWLRKVKH	GHFGWPGWEM	GPPGNWSPRP
PRAGEARPGP	TAESASGPSE	DPSVNFLKNV	GESVAAALSP	LGIEVDIDVE	HGGKRSRLTP	VSPESSSTEE
KSSSQPSSCC	SDPSKPGGNV	EGATQSLAEQ	MRKIALESEG	RPEEQMESDN	CSGGDDDWTH	LSSKEVDPST
GELQSLQMPE	SEGPSSLDPS	QEGPTGLKEA	ALYPHLPPEA	DPRLIESLSQ	MLSMGFSDEG	GWLTRLLQTK
NYDIGAALDT	IQYSKHPPPL					

IPI00023122 (100%), 49,844.4 Da
Isoform 1 of PDZ and LIM domain protein 7
4 unique peptides, 4 unique spectra, 4 total spectra, 45/457 amino acids (10% coverage)

MDSFKVVLEG	PAPWGFRLQG	GKDFNVPLSI	SRLTPGGKAA	QAGVAVGDWV	LSIDGENAGS	LTHIEAQNKI
RACGERLSLG	LSRAQPVQSK	PQKASAPAAD	PPRYTFAPSV	SLNKTARPFG	APPPADSAPQ	QNGQPLRPLV
PDASKQRLME	NTEDWRPRPG	TGQSRSFRIL	AHLTGTEFMQ	DPDEEHLKKS	SQVPRTEAPA	PASSTPQEPW
PGPTAPSPTS	RPPWAVDPAF	AERYAPDKTS	TVLTRHSQPA	TPTPLQSRTS	IVQAAAGGVP	GGGSNNGKTP
VCHQCHKVIR	GRYLVALGHA	YHPEEFVCSQ	CGKVLEEGGF	FEEKGAIFCP	PCYDVRYAPS	CAKCKKKITG
EIMHALKMTW	HVHCFTCAAC	KTPIRNRAFY	MEEGVPYCER	DYEKMFGTKC	HGCDFKIDAG	DRFLEALGFS
WHDTCFVCAI	CQINLEGKTF	YSKKDRPLCK	SHAFSHV			

IPI00218398 (100%), 65,895.8 Da
Matrix metalloproteinase-14
2 unique peptides, 2 unique spectra, 2 total spectra, 17/582 amino acids (3% coverage)

MSPAPRPPRC	LLLPLLTLGT	ALASLGSAQS	SSFSPEAWLQ	QYGYLPPGDL	RTHTQRSPQS	LSAAIAAMQK
FYGLQVTGKA	DADTMKAMRR	PRCGVPDKFG	AEIKANVRRK	RYAIQGLKWQ	HNEITFCIQN	YTPKVGEYAT
YEAIRKAFRV	WESATPLRFR	EVPYAYIREG	HEKQADIMIF	FAEGFHGDST	PFDGEGGFLA	HAYFPGPNIG
GDTHFDSAEP	WTVRNEDLNG	NDIFLVAVHE	LGHALGLEHS	SDPSAIMAPF	YQWMDTENFV	LPDDDRRGIQ
QLYGGESGFP	TKMPPQPRTT	SRPSVPDKPK	NPTYGPNICD	GNFDTVAMLR	GEMFVFKERW	FWRVRNNQVM
DGYPMPIGQF	WRGLPASINT	AYERKDGKFV	FFKGDKHWVF	DEASLEPGYP	KHIKELGRGL	PTDKIDAALF
WMPNGKTYFF	RGNKYYRFNE	ELRAVDSEYP	KNIKVWEGIP	ESPRGSFMGS	DEVFTYFYKG	NKYWKFNNQK
LKVEPGYPKS	ALRDWMGCPS	GGRPDEGTEE	ETEVIIIEVD	EEGGGAVSAA	AVVLPVLLLL	LVLAVGLAVF
FFRRHGTPRR	LLYCQRSLLD	KV				

IPI00021263 (100%), 27,745.9 Da
14-3-3 protein zeta/delta
2 unique peptides, 2 unique spectra, 2 total spectra, 26/245 amino acids (11% coverage)

MDKNELVQKA	KLAEQAERYD	DMAACMKSVT	EQGAELSNEE	RNLLSVAYKN	VVGARRSSWR	VVSSIEQKTE
GAEKKQQMAR	EYREKIETEL	RDICNDVLSL	LEKFLIPNAS	QAESKVFYLK	MKGDYYRYLA	EVAAGDDKKG
IVDQSQQAYQ	EAFEISKKEM	QPTHPIRLGL	ALNFSVFYYE	ILNSPEKACS	LAKTAFDEAI	AELDTLSEES
YKDSTLIMQL	LRDNLTLWTS	DTQGDEAEAG	EGGEN			

IPI00010796 (100%), 57,118.1 Da
Protein disulfide-isomerase
2 unique peptides, 2 unique spectra, 2 total spectra, 19/508 amino acids (4% coverage)

MLRRALLCLA VAALVRADAP EEEDHVLVLR KSNFAEALAA HKYLLVEFYA PWCGHCKALA PEYAKAAGK**L**
KAEGSEIRLA KVDATEESDL AQQYGVRGYP TIKFFRNGDT ASPKEYTAGR **EADDIVNWLK** KRTGPAATTL
PDGAAAESLV ESSEVAVIGF FKDVESDSAK QFLQAAEAID DIPFGITSNS DVFSKYQLDK DGVVLFKKFD
EGRNNFEGEV TKENLLDFIK HNQLPLVIEF TEQTAPKIFG GEIKTHILLF LPKSVSDYDG KLSNFKTAAE
SFKGKILFIF IDSDHTDNQR ILEFFGLKKE ECPAVRLITL EEEMTKYKPE SEELTAERIT EFCHRFLEGK
IKPHLMSQEL PEDWDKQPVK VLVGKNFEDV AFDEKKNVFV EFYAPWCGHC KQLAPIWDKL GETYKDHENI
VIAKMDSTAN EVEAVKVHSF PTLKFFPASA DRTVIDYNGE RTLDGFKKFL ESGGQDGAGD DDDLEDLEEA
EEPDMEEDDD QKAVKDEL
